# Supplementary material for: Uptake of the multi-arm multi-stage (MAMS) adaptive platform approach: a trial-registry review of late-phase randomised clinical trials
Source: BMJ Open. 2022 Mar 10;12(3):e055615. doi: 10.1136/bmjopen-2021-055615 (PMC8915371; doi:10.1136/bmjopen-2021-055615)
Supplement: Supplementary data [file bmjopen-2021-055615supp001.pdf]

Figure S1. Cumulative uptake of MAMS platform protocol registrations for late-phase trials prior to and following the COVID-19 pandemic.

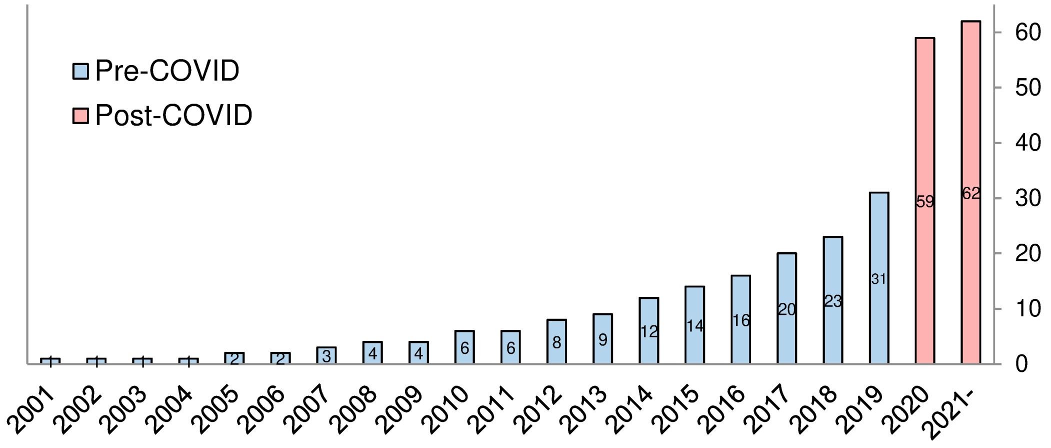

Figure S2. Anticipated maximum sample size at outset of late-phase MAMS platform trials.

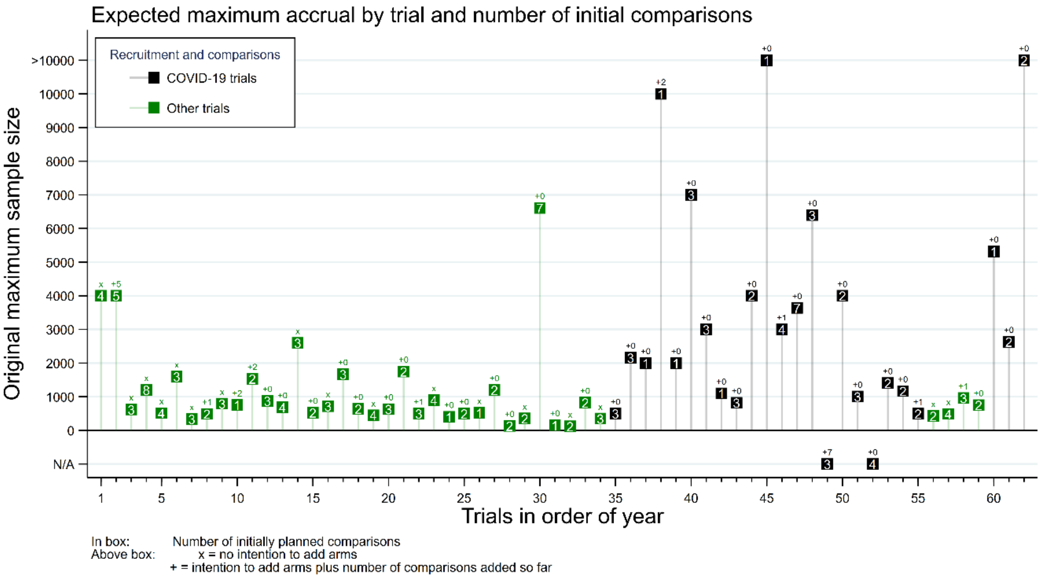

Table S1. Search strategy and string used to identify late-phase MAMS platform trials.

Search strategy and string used to identify late-phase MAMS platform trials.
We examined the uptake of registered, late-phase MAMS platform trials by conducting searches both in individual registries including ClinicalTrials.gov, the EU clinical trials register, which contains all trials registered on the European Union Drug Regulating Authorities Clinical Trials Database (EudraCT), the Australian New Zealand Clinical Trials Registry (ANZCTR), International Standard Randomised Controlled Trial Number (ISRCTN) registry, the Pan African Clinical Trials Registry (PACTR), and the overall World Health Organisation (WHO) International Clinical Trial Registry Platform (ICTRP), which is a summary of worldwide trial databases. We also searched the databases PubMed, Medline, Cochrane Library, Global Health Library, and EMBASE using a structured search strategy for published trials. The final search strategy was completed on 1 April 2021. For published articles, both the titles and abstracts were searched. Publications and/or trial registry results could include abstracts/trial registrations in a language other than English, if supplied by the publisher/entrant, but defaulted to the English abstract where an entry existed. No filters or limits were applied.<br><br>The following search terms used were: “adaptive trial” OR “adaptive clinical trial” OR “platform trial” OR “platform clinical trial” OR “adaptive platform trial” OR “adaptive platform clinical trial”. “multi-arm multi-stage” OR “MAMS”. “master protocol” OR “master trial protocol” OR “trial master protocol”. “late-phase” OR “phase 3” OR “phase 2-3” OR “phase 2/3” OR “phase 2 to 3” OR “seamless phase 2/3” OR “seamless phase 2-3”. “seamless” OR “seamless design” OR “adaptive seamless” OR “adaptive seamless design”, singly or in combination.<br><br>All late-phase MAMS platform trials included in this review were registered on a trial registry by 1 April 2021, which was the final day of identification and data collection. This was the date used to lock the data for this manuscript, including both registration of a trial and information about ongoing trial progress. Single extraction of data took place with subsequent review and verification of findings

following discussion with collaborators and colleagues to ensure no missing or inappropriately included trials were in the final list. Information about trials was obtained from data linked to trial registry numbers, including data from registry entries, publications (if present and linked to trial registry number), trial websites if link was provided on trial registry entry, and review of publicly available documents such as trial protocols and statistical analysis plans if they were linked to the trial registry entry.

Table S2. Characteristics by individual trial for the design, co-ordination and recruitment of late-phase MAMS platform protocols.

Name	MAMS term used	Phase of trial	Organisation leading	Country of leading organisation	Number of recruiting countries
**2001**					
ICON5	Yes	3	Non-commercial	UK	1
**2005**					
STAMPEDE	Yes	Seamless 2/3	Non-commercial	UK	2
**2007**					
ADVENT	No	3	Commercial	USA	1
**2008**					
AWARD-5	No	Seamless 2/3	Commercial	USA	1
**2010**					
Infantile haemangioma	No	Seamless 2/3	Commercial	France	1
MODIFY-I	No	3	Commercial	USA	19
**2012**					
DIAN-TU	No	Seamless 2/3	Non-commercial	USA	9
TAILOR	Yes	Seamless 2/3 option	Non-commercial	UK	1
**2013**					
ESETT	No	3	Commercial	USA	1
**2014**					
FOCUS4	Yes	Seamless 2/3 option	Non-commercial	UK	1
REECUR	Yes	Seamless 2/3	Non-commercial	UK	16
FLAIR	No	3	Non-commercial	UK	1
**2015**					
COMPARE	Yes	3	Non-commercial	UK	1
SEPSIS-ACT	No	Seamless 2/3 option	Commercial	USA	5
**2016**					
CIRCUITS	Yes	3	Non-commercial	UK	1
PARTNER	No	Seamless 2/3	Non-commercial	UK	1
**2017**					
Intergroup LEAP	No	Seamless 2/3	Non-commercial	USA	1
MIDFUT	Yes	Seamless 2/3	Non-commercial	UK	1
PLATO ACT5	Yes	Seamless 2/3	Non-commercial	UK	1
TB-PRACTECAL	Yes	Seamless 2/3	Non-commercial	Netherlands	3
PALM	No	Seamless 2/3	Non-commercial	USA	1
RAMPART	Yes	3	Non-commercial	UK	5
TRUNCATE-TB	Yes	Seamless 2/3	Non-commercial	Singapore	4
**2018**					
CHRONOS-B	Yes	Seamless 2/3	Non-commercial	UK	1
DEMEL	Yes	Seamless 2/3	Non-commercial	France	1
GBM-AGILE	No	Seamless 2/3	Non-commercial	USA	2
MAGMA	Yes	3	Non-commercial	Australia	1
PROSTATEGY	Yes	Seamless 2/3	Non-commercial	Spain	1
ROSSINI2	Yes	3	Non-commercial	UK	1
TACE-3	Yes	Seamless 2/3	Non-commercial	UK	2
**2020**					
CAN-STAMP	Yes	Seamless 2/3	Non-commercial	Canada	1
HEALEY ALS	No	Seamless 2/3	Non-commercial	USA	1
IRISS	Yes	3	Non-commercial	France	1

Name	MAMS term used	Phase of trial	Organisation leading	Country of leading organisation	Number of recruiting countries
MND-SMART	Yes	Seamless 2/3	Non-commercial	UK	1
MODULATE	Yes	Seamless 2/3	Non-commercial	UK	1
OSONE	Yes	3	Non-commercial	France	1
Precision Promise	No	Seamless 2/3	Non-commercial	USA	1
RadIO	No	Seamless 2/3	Non-commercial	UK	1
ACOVACT	No	Seamless 2/3	Non-commercial	Austria	1
ACTIV-1	No	3	Non-commercial	USA	1
ACTIV-2	No	Seamless 2/3	Non-commercial	USA	1
ACTIV-3 TICO	Yes	3	Non-commercial	USA	5
ACTIV-4a	No	3	Non-commercial	USA	2
ACTIV-4b	No	3	Non-commercial	USA	1
ANTICOV	No	3	Non-commercial	Switzerland	13
CCAP	No	3	Non-commercial	Denmark	1
COVERAGE	Yes	Seamless 2/3	Non-commercial	France	1
COVIDMED	No	Seamless 2/3	Commercial	USA	1
CROWN-CORONATION	No	3	Non-commercial	USA	6
PRINCIPLE	No	3	Non-commercial	UK	1
PROTECT	Yes	3	Non-commercial	Pakistan	1
PROTECT-Surg	Yes	3	Non-commercial	UK	9
RECOVERY	Yes	3	Non-commercial	UK	3
RECOVERY-RS	Yes	3	Non-commercial	UK	1
REVOLUTIOn	Yes	Seamless 2/3	Non-commercial	Brazil	1
SOLIDARITY	No	3	Non-commercial	Switzerland	30
TACTIC-E	No	Seamless 2/3	Non-commercial	UK	1
TACTIC-R	No	3	Non-commercial	UK	1
Treatment for COVID	No	Seamless 2/3	Non-commercial	USA	1
**2021**					
ACTIV-4c	No	3	Non-commercial	USA	1
HEAL-COVID	No	3	Non-commercial	UK	1
PROTECT-CH	Yes	3	Non-commercial	UK	1

Table S3. Characteristics for trial sponsors and interventions assessed in late-phase MAMS platform trials.

Name	Type of interventions	Sponsor	Repurposed intervention(s)	Doses of intervention	Durations of intervention
**2001**					
ICON5	Medical	Non-commercial	None	No	No
**2005**					
STAMPEDE	Medical	Non-commercial	Some	No	No
**2007**					
ADVENT	Medical	Commercial	None	Yes	No
**2008**					
AWARD-5	Medical	Commercial	None	Yes	No
**2010**					
Infantile haemangioma	Medical	Commercial	All	Yes	No
MODIFY-I	Medical	Commercial	None	No	No
**2012**					
DIAN-TU	Medical	Non-commercial	None	Yes	No
TAILOR	Medical	Non-commercial	All	Yes	No
**2013**					
ESETT	Medical	Commercial	All	No	No
**2014**					
FOCUS4	Medical	Non-commercial	Some	No	No
REECUR	Medical	Non-commercial	None	No	No
FLAIR	Medical	Non-commercial	None	No	No
**2015**					
COMPARE	Medical and surgical	Non-commercial	None	No	No
SEPSIS-ACT	Medical	Commercial	None	Yes	No
**2016**					
CIRCUITS	Psychiatric	Non-commercial	N/A	Yes	No
PARTNER	Medical	Non-commercial	None	No	Yes
**2017**					
Intergroup LEAP	Medical	Non-commercial	None	No	No
MIDFUT	Medical and surgery	Non-commercial	None	No	No
PLATO ACT5	Medical	Non-commercial	N/A	Yes	No
TB-PRACTECAL	Medical	Non-commercial	None	No	Yes
**2018**					
PALM	Medical	Non-commercial	None	No	No
RAMPART	Medical	Non-commercial	None	No	No
TRUNCATE-TB	Medical	Non-commercial	None	No	No
**2019**					
CHRONOS-B	Medical	Non-commercial	None	No	No
DEMEL	Medical	Non-commercial	Yes	Yes	No
GBM-AGILE	Medical	Non-commercial	None	No	No
MAGMA	Medical	Non-commercial	None	No	Yes
PROSTATEGY	Medical	Non-commercial	None	No	No
ROSSINI2	Surgery	Non-commercial	N/A	No	No
TACE-3	Medical	Non-commercial	None	No	No
**2020**					
CAN-STAMP	Medical	Non-commercial	None	No	No

Name	Type of interventions	Sponsor	Repurposed intervention(s)	Doses of intervention	Durations of intervention
HEALEY ALS	Medical	Non-commercial	None	No	No
IRISS	Medical	Non-commercial	All	Yes	No
MND-SMART	Medical	Non-commercial	Some	No	No
MODULATE	Medical	Non-commercial	All	No	No
OSONE	Medical	Non-commercial	N/A	Yes	No
Precision Promise	Medical	Non-commercial	None	No	No
RadIO	Medical	Non-commercial	None	No	No
ACOVACT	Medical	Non-commercial	Some	No	No
ACTIV-1	Medical	Non-commercial	All	No	No
ACTIV-2	Medical	Non-commercial	None	No	No
ACTIV-3 TICO	Medical	Non-commercial	None	No	No
ACTIV-4a	Medical	Non-commercial	All	Yes	No
ACTIV-4b	Medical	Non-commercial	All	No	No
ANTICOV	Medical	Non-commercial	All	No	No
CCAP	Medical	Non-commercial	None	No	No
COVERAGE	Medical	Non-commercial	All	No	No
COVIDMED	Medical	Commercial	All	No	No
CROWN-CORONATION	Medical	Non-commercial	All	No	No
PRINCIPLE	Medical	Non-commercial	All	No	No
PROTECT	Medical	Non-commercial	All	No	No
PROTECT-Surg	Medical	Non-commercial	All	No	No
RECOVERY	Medical	Non-commercial	Some	No	No
RECOVERY-RS	Medical	Non-commercial	N/A	No	No
REVOLUTIOn	Medical	Non-commercial	All	No	No
SOLIDARITY	Medical	Non-commercial	Some	No	No
TACTIC-E	Medical	Non-commercial	None	No	No
TACTIC-R	Medical	Non-commercial	All	No	No
Treatment for COVID	Medical	Non-commercial	All	No	No
**2021**					
ACTIV-4c	Medical	Non-commercial	All	No	No
HEAL-COVID	Medical	Non-commercial	All	No	No
PROTECT-CH	Medical	Non-commercial	Yes	No	No

Table S4. Characteristics for intervention arms, control group and anticipated initial maximum sample size across late-phase MAMS platform trials.

Name	Number of arms at outset	Number of arms assessed in total	Plan to add arms	Actually added arms	Control group	Planned maximum sample size
**2001**						
ICON5	4	4	No	No	SOC	4000
**2005**						
STAMPEDE	5	10	Yes	Yes	SOC	4000
**2007**						
ADVENT	3	3	No	No	Placebo	624
**2008**						
AWARD-5	8	8	No	No	Placebo and SOC	1202
**2010**						
Infantile haemangioma	4	4	No	No	Placebo	512
MODIFY-I	3	3	No	No	Placebo and SOC	1600
**2012**						
DIAN-TU	2	3	Yes	Yes	Placebo	490
TAILOR	3	3	No	No	SOC	336
**2013**						
ESETT	3	3	No	No	None	795
**2014**						
FOCUS4	2	4	Yes	Yes	Placebo	1536
REECUR	3	3	es	No	SOC	875
FLAIR	1	3	Yes	Yes	SOC	754
**2015**						
COMPARE	4	4	Yes	No	SOC	685
SEPSIS-ACT	3	3	No	No	Placebo	2600
**2016**						
CIRCUITS	3	3	No	No	SOC	720
PARTNER	2	2	Yes	No	SOC	527
**2017**						
Intergroup LEAP	3	3	Yes	No	SOC	1670
MIDFUT	4	4	No	No	SOC	447
PLATO ACT5	2	2	Yes	No	SOC	640
TB-PRACTECAL	3	3	Yes	No	SOC	630
PALM	3	4	Yes	Yes	None	500
RAMPART	2	2	Yes	No	SOC	1750
TRUNCATE-TB	4	4	No	No	SOC	900
**2018**						
CHRONOS-B	2	2	Yes	No	SOC	1200
DEMEL	2	2	No	No	Placebo	355
GBM-AGILE	1	1	Yes	No	SOC	400
MAGMA	2	2	Yes	No	SOC	500
PROSTATEGY	2	2	Yes	No	SOC	135
ROSSINI2	7	7	Yes	No	SOC	6610
TACE-3	1	1	No	No	SOC	522
**2020**						
CAN-STAMP	2	2	No	No	None	120
HEALEY ALS	3	4	Yes	Yes	Placebo	960
IRISS	2	2	No	No	Placebo	429

Name	Number of arms at outset	Number of arms assessed in total	Plan to add arms	Actually added arms	Control group	Planned maximum sample size
MND-SMART	2	2	Yes	No	Placebo	750
MODULATE	4	4	No	No	SOC	491
OSONE	3	3	No	No	SOC	350
Precision Promise	2	2	Yes	No	SOC	825
RadIO	1	1	Yes	No	SOC	159
ACOVACT	3	3	Yes	No	SOC	500
ACTIV-1	3	3	Yes	No	SOC	2160
ACTIV-2	1	1	Yes	No	SOC	2000
ACTIV-3 TICO	1	3	Yes	Yes	Placebo and SOC	10000
ACTIV-4a	1	1	Yes	No	SOC	2000
ACTIV-4b	3	3	Yes	No	Placebo	7000
ANTICOV	3	3	Yes	No	SOC	3000
CCAP	1	1	Yes	No	Placebo	1100
COVERAGE	3	3	Yes	No	Placebo	820
COVIDMED	2	2	Yes	No	Placebo	4000
CROWN-CORONATION	1	1	Yes	Yes	Placebo	30000
PRINCIPLE	4	6	Yes	Yes	SOC	3000
PROTECT	7	7	Yes	No	None	3640
PROTECT-Surg	3	3	Yes	No	SOC	6400
RECOVERY	3	10	Yes	Yes	SOC	N/A
RECOVERY-RS	2	2	Yes	No	SOC	4000
REVOLUTIOn	3	3	Yes	No	Placebo	1005
SOLIDARITY	4	4	Yes	Yes	SOC	N/A
TACTIC-E	2	2	Yes	No	SOC	1407
TACTIC-R	2	2	Yes	No	SOC	1167
Treatment for COVID	2	3	Yes	No	Placebo	495
**2021**						
ACTIV-4c	1	1	Yes	Yes	Placebo	5320
HEAL-COVID	2	2	Yes	Yes	SOC	2631
PROTECT-CH	2	2	Yes	Yes	SOC	12000

Table S5. Name, trial registry entry and trial registration number of late-phase MAMS platform trials.

Name	Trial registration number	Trial registry
ICON5	NCT00011986	Clinicaltrials.gov
STAMPEDE	NCT00268476	Clinicaltrials.gov
ADVENT	NCT00547898 and NCT01374490	Clinicaltrials.gov
AWARD-5	NCT00734474	Clinicaltrials.gov
Infantile haemangioma	NCT01056341	Clinicaltrials.gov
MODIFY-I	NCT01241552	Clinicaltrials.gov
DIAN-TU	NCT01760005	Clinicaltrials.gov
TAILOR	ISRCTN51069819	ISRCTN registry
ESETT	NCT01960075	Clinicaltrials.gov
FOCUS4	ISRCTN90061546	ISRCTN registry
REECUR	ISRCTN36453794	ISRCTN registry
FLAIR	ISRCTN01844152	ISRCTN registry
COMPARE	NCT04116047	Clinicaltrials.gov
SEPSIS-ACT	NCT02508649	Clinicaltrials.gov
CIRCUITS	ISRCTN14678860	ISRCTN registry
PARTNER	NCT03150576	Clinicaltrials.gov
Intergroup LEAP	NCT03092674	Clinicaltrials.gov
MIDFUT	ISRCTN64926597	ISRCTN registry
PLATO ACT5	ISRCTN88455282	ISRCTN registry
TB-PRACTECAL	NCT02589782	Clinicaltrials.gov
PALM	NCT03719586	Clinicaltrials.gov
RAMPART	NCT03288532	Clinicaltrials.gov
TRUNCATE-TB	NCT03474198	Clinicaltrials.gov
CHRONOS-B	NCT04049747	Clinicaltrials.gov
DEMEL	NCT03524937	Clinicaltrials.gov
GBM-AGILE	NCT03970447	Clinicaltrials.gov
MAGMA	ACTRN12618001944224	Australian New Zealand Clinical Trials Registry
PROSTATEGY	NCT03879122	Clinicaltrials.gov
ROSSINI2	NCT03838575	Clinicaltrials.gov
TACE-3	ISRCTN12053408	ISRCTN registry
CAN-STAMP	NCT04159155	Clinicaltrials.gov
HEALEY ALS	NCT04297683	Clinicaltrials.gov
IRISS	NCT04031573	Clinicaltrials.gov
MND-SMART	NCT04302870	Clinicaltrials.gov
MODULATE	ISRCTN16086699	ISRCTN registry
OSONE	NCT03976180	Clinicaltrials.gov
Precision Promise	NCT04229004	Clinicaltrials.gov
RadIO	ISRCTN43698103	ISRCTN registry
ACOVACT	NCT04351724	Clinicaltrials.gov
ACTIV-1	NCT04593940	Clinicaltrials.gov
ACTIV-2	NCT04518410	Clinicaltrials.gov
ACTIV-3 TICO	NCT04501978	Clinicaltrials.gov
ACTIV-4a	NCT04505774	Clinicaltrials.gov
ACTIV-4b	NCT04498273	Clinicaltrials.gov
ANTICOV	PACTR202006537901307	Pan African Clinical Trials Registry
CCAP	NCT04345289	Clinicaltrials.gov
COVERAGE	NCT04356495	Clinicaltrials.gov
COVIDMED	NCT04328012	Clinicaltrials.gov
CROWN-CORONATION	NCT04333732	Clinicaltrials.gov

Name	Trial registration number	Trial registry
PRINCIPLE	ISRCTN86534580	ISRCTN registry
PROTECT	NCT04338698	Clinicaltrials.gov
PROTECT-Surg	NCT04386070	Clinicaltrials.gov
RECOVERY	ISRCTN50189673	ISRCTN registry
RECOVERY-RS	ISRCTN126912075	ISRCTN registry
REVOLUTIOn	NCT04468087	Clinicaltrials.gov
SOLIDARITY	ISRCTN83971151	ISRCTN registry
TACTIC-E	NCT04393246	Clinicaltrials.gov
TACTIC-R	NCT04390464	Clinicaltrials.gov
Treatment for COVID	NCT04354428	Clinicaltrials.gov
ACTIV-4c	NCT04650087	Clinicaltrials.gov
PROTECT-CH	EudraCT2021-000185-15	EU clinical trials register
HEAL-COVID	NCT04801940	Clinicaltrials.gov
