# Supplementary material for: Uptake of the multi-arm multi-stage (MAMS) adaptive platform approach: a trial-registry review of late-phase randomised clinical trials
Source: BMJ Open. 2022 Mar 10;12(3):e055615. doi: 10.1136/bmjopen-2021-055615 (PMC8915371; doi:10.1136/bmjopen-2021-055615)
Supplement: Author's manuscript [file bmjopen-2021-055615.draft_revisions.pdf]

BMJ Open

BMJ Open is committed to open peer review. As part of this commitment we make the peer review history of every article we publish publicly available.

When an article is published we post the peer reviewers' comments and the authors' responses online. We also post the versions of the paper that were used during peer review. These are the versions that the peer review comments apply to.

The versions of the paper that follow are the versions that were submitted during the peer review process. They are not the versions of record or the final published versions. They should not be cited or distributed as the published version of this manuscript.

BMJ Open is an open access journal and the full, final, typeset and author-corrected version of record of the manuscript is available on our site with no access controls, subscription charges or pay-per-view fees (http://bmjopen.bmj.com).

If you have any questions on BMJ Open's open peer review process please email info.bmjopen@bmj.com

BMJ Open

# Uptake of the multi-arm multi-stage (MAMS) adaptive platform approach for late-phase randomised clinical trials: a trial-registry review

| | |
|---|---|
| Journal: | *BMJ Open* |
| Manuscript ID | bmjopen-2021-055615 |
| Article Type: | Original research |
| Date Submitted by the Author: | 20-Jul-2021 |
| Complete List of Authors: | Noor, Nurulamin; MRC Clinical Trials Unit at UCL<br>Love, Sharon; MRC Clinical Trials Unit at UCL<br>Isaacs, Talia; University College London, Institute of Education<br>Kaplan, Richard; MRC Clinical Trials Unit at UCL<br>Parmar, Mahesh; MRC Clinical Trials Unit at UCL<br>Sydes, Matthew; MRC Clinical Trials Unit at UCL |
| Keywords: | Clinical trials < THERAPEUTICS, PUBLIC HEALTH, INFECTIOUS DISEASES, ONCOLOGY, CLINICAL PHARMACOLOGY |
| | |

SCHOLARONE™
Manuscripts

*I, the Submitting Author has the right to grant and does grant on behalf of all authors of the Work (as defined in the below author licence), an exclusive licence and/or a non-exclusive licence for contributions from authors who are: i) UK Crown employees; ii) where BMJ has agreed a CC-BY licence shall apply, and/or iii) in accordance with the terms applicable for US Federal Government officers or employees acting as part of their official duties; on a worldwide, perpetual, irrevocable, royalty-free basis to BMJ Publishing Group Ltd ("BMJ") its licensees and where the relevant Journal is co-owned by BMJ to the co-owners of the Journal, to publish the Work in this journal and any other BMJ products and to exploit all rights, as set out in our licence.*

*The Submitting Author accepts and understands that any supply made under these terms is made by BMJ to the Submitting Author unless you are acting as an employee on behalf of your employer or a postgraduate student of an affiliated institution which is paying any applicable article publishing charge ("APC") for Open Access articles. Where the Submitting Author wishes to make the Work available on an Open Access basis (and intends to pay the relevant APC), the terms of reuse of such Open Access shall be governed by a Creative Commons licence – details of these licences and which Creative Commons licence will apply to this Work are set out in our licence referred to above.*

*Other than as permitted in any relevant BMJ Author's Self Archiving Policies, I confirm this Work has not been accepted for publication elsewhere, is not being considered for publication elsewhere and does not duplicate material already published. I confirm all authors consent to publication of this Work and authorise the granting of this licence.*

For peer review only

# Uptake of the multi-arm multi-stage (MAMS) adaptive platform approach for late-phase randomised clinical trials: a trial-registry review

Nurulamin M. Noor (0000-0003-3426-6408)[1*], Sharon B. Love (0000-0002-6695-5390)[1], Talia Isaacs (0000-0003-4302-3379)[2], Richard Kaplan (0000-0002-0189-8348)[1], Mahesh K.B. Parmar (0000-0003-0166-1700)[1], Matthew R. Sydes (0000-0002-9323-1371)[1].

[1] MRC Clinical Trials Unit at University College London, Institute of Clinical Trials and Methodology, London, United Kingdom.

[2] UCL Institute of Education, University College London, London, United Kingdom.

[*] Corresponding author: Dr Nurulamin Noor, Medical Research Council Clinical Trials Unit at University College London, London, United Kingdom

Email: noorn@doctors.org.uk



For peer review only - http://bmjopen.bmj.com/site/about/guidelines.xhtml

## Abstract

### Background

For medical conditions with numerous interventions worthy of investigation, there are many advantages of using a multi-arm multi-stage (MAMS) platform trial approach. However, there is currently limited knowledge on the uptake of the MAMS platform design, especially in the late-phase setting.

### Design

We examined the uptake of registered, late-phase MAMS platform trials. The review data was frozen on 1 April 2021. MAMS platform trials were defined as requiring two or more comparison arms, with two or more trial stages, with an interim analysis allowing for stopping of recruitment to arms and typically the ability to add in new intervention arms.

### Results

62 late-phase clinical trials using a MAMS approach were included. Overall, the number of trials using the MAMS design in the late-phase setting has been increasing since 2001 and has been accelerated by coronavirus disease 2019 (COVID-19). The majority of current MAMS platform trials were either targeting infectious diseases (52%) or cancers (29%) and all the MAMS platform trials identified were trials of treatment interventions. 89% (55/62) of the MAMS platform trials were focused on evaluation of drug medications, with 45% (28/62) of the MAMS platforms having at least one or more repurposed medication as a comparison intervention arm.

### Conclusions


For peer review only

Historically, late-phase trials have adhered to long-established standard (two-arm) designs. However, the number of late-phase MAMS platform trials is increasing, across a range of different disease areas. This study highlights the potential scope of MAMS platform trials and may assist research teams considering use of this approach in the late-phase RCT setting.

Keywords: multi-arm, multi-stage, MAMS, adaptive, platform, clinical trial, late-phase, master protocol, uptake, trial registry-review

For peer review only

**Strengths and limitations of this study**

- This study builds on previous publications reporting on uptake of adaptive or platform trials in general, by reporting the uptake and characteristics of MAMS platform late-phase RCTs over the past 20 years.
- The search strategy used did not include phase 1 or phase 2-only MAMS platform trials, however this was because of the quite different considerations for trial teams designing and conducting early-phase exploratory compared to late-phase confirmatory RCTs.
- By conducting an extensive trial registry review and including all the most recent trial registrations, this study reports a much wider uptake of the MAMS platform design than previously suggested, across a large number of countries and different disease areas.
- It is important to note that adaptive trials by their nature are fast-moving area and there will have been further trial registrations since the April 2021 coverage in this manuscript. The information presented about included trials was accurate and up-to-date at the time of writing.
- We believe that reporting on the uptake and characteristics across these currently registered MAMS platform trials, enables detailed consideration and better planning for teams considering future use of these efficient designs.



For peer review only - http://bmjopen.bmj.com/site/about/guidelines.xhtml

## Introduction

Randomised clinical trials (RCTs) are the gold standard for investigating healthcare interventions. Late-phase RCTs in particular play an important role in shaping health policy, guidelines and informing clinical practice to ensure better outcomes for patients and the public.[1] Historically, for most medical conditions, there have been few therapeutic options and long lag-times for developing new treatments. Thus, traditional two-arm, parallel-group RCTs were appropriate to perform in many instances.[2] However, nowadays there are multiple potential interventions available for testing in many medical conditions, and with patients, clinicians, and healthcare systems demanding faster answers to clinical questions. It is clearly neither efficient nor expedient to continue with this approach of multiple, separate, individual two-arm RCTs. In addition, there are many more aspects of treatments to evaluate than ever before including: doses, durations, and combinations of interventions.

Despite the rapidly rising number of registered clinical trials being performed around the world,[3] there are growing concerns about the length of time and increasing costs of performing late-phase RCTs.[4] Whilst most late-phase RCTs do reach a conclusion, only 30-40% are able to demonstrate efficacy of an intervention, sometimes referred to as “positive” trials. [5,6] We would caution against the use of use of terms such as “positive” or “negative” and highlight the growing literature suggesting against use of these terms to describe RCT findings.[7,8] Nevertheless, these results do highlight that there is a pressing need to speed up the evaluation process in late-phase RCTs. In line with this, there has been a movement towards “faster, better, more efficient” trials.[9] Indeed, it is now well recognised that for



For peer review only - http://bmjopen.bmj.com/site/about/guidelines.xhtml

conditions for which there are multiple interventions worthy of investigation, consideration should be given to using multi-arm trial designs.[10]

A range of different trial designs have been proposed which have been classified by some under the umbrella term of complex, innovative designs (CIDs).[11] The adaptive platform trial (APT) approach using a multi-arm multi-stage (MAMS) protocol is one such design and offers many solutions to the above-described problems of traditional trial designs.[12] The MAMS platform trial typically uses a single master protocol to allow multiple primary research questions to be answered, compared to the one primary research question that is answered in a traditional two-arm trial.[13] Multiple interventions can be assessed in parallel and the shared control arm allows immediate efficiency saving by reducing the overall recruitment needed. Once participants are enrolled, information accumulates during the course of the trial and adaptations can be made, which use accumulating trial data to modify aspects of the ongoing trial.[14] These adaptations are typically made following interim analysis and according to predefined adaptations,[15] to ensure they preserve validity and integrity of the trial. Crucially these adaptations are made via amendment to a trial protocol rather than having to register, setup and conduct a separate, new trial entirely.[16]

For peer review only

To date, the uptake of adaptive designs has been lower for late-phase RCTs,[17] compared to early-phase RCTs.[18] There are likely many reasons for lower uptake in late-phase settings, including: greater reliance on findings from late-phase confirmatory trials for regulatory purposes including marketing authorisation of medications, as well as more limited knowledge and lack of formal guidance about how best to apply these newer designs to the late-phase setting. However, given the



For peer review only - http://bmjopen.bmj.com/site/about/guidelines.xhtml

high costs of late-phase trial programmes,[4] and the significant commercial impact from failure to demonstrate efficacy,[19] the “early get out” offered by stopping recruitment to interventions showing lack of benefit, or harm, makes the MAMS design potentially applicable across many different disease areas in the late-phase setting.[20]

Previous studies have focused on the uptake of adaptive trials in general,[18,21] across both early and late-phase settings,[22] or platform trials in general,[23] but without specific focus on the MAMS approach. Additionally due to the varied methods used, including restricting of searching to trial publications,[24] many ongoing MAMS platform trials, including recently registered trials have not historically been captured. Despite anecdotal reports for increasing use of the MAMS platform design in late-phase settings, there is currently, no study documenting how extensive this uptake has been. Accordingly, we present a trial registry-based review to examine the worldwide uptake of APTs using MAMS protocols in the late-phase randomised clinical trial setting.



For peer review only - http://bmjopen.bmj.com/site/about/guidelines.xhtml

## Methods

We conducted a systematic search for APTs using MAMS protocols in late-phase trial settings. An initial search was performed by searching the databases PubMed, Medline, Cochrane Library, Global Health Library, and EMBASE using a structured search strategy for published trials. However, given the knowledge and awareness that many MAMS platform trials would be unpublished, and the recognised importance of registering clinical trials, this review predominantly extracted data from global trial registries. Searches were conducted both in individual trial registries including ClinicalTrials.gov, the EU clinical trials register, which contains all trials registered on the European Union Drug Regulating Authorities Clinical Trials Database (EudraCT), the Australian New Zealand Clinical Trials Registry (ANZCTR), International Standard Randomised Controlled Trial Number (ISRCTN) registry, the Pan African Clinical Trials Registry (PACTR), and the overall World Health Organisation (WHO) International Clinical Trial Registry Platform (ICTRP), which is a summary of worldwide trial databases. This enabled us to identify any further registered but unpublished trials.

For published articles, both the titles and abstracts were searched. Publications and trial registry results could include abstracts/trial registrations in a language other than English, if supplied by the publisher/entrant, but defaulted to the English abstract where an entry existed. Combinations for each of the following search terms were used, “multi-arm multi-stage”, “MAMS”, “adaptive”, “platform”, “trial”, “master protocol”, “late-phase”, “seamless”, “phase two”, “phase three”. All the MAMS platform trials initially identified in the published literature reported an international clinical trial registry number and could subsequently be linked to a trial registration. All late-phase



For peer review only - http://bmjopen.bmj.com/site/about/guidelines.xhtml

MAMS platform trials included in this review were registered on a trial registry by 1 April 2021, which was the final day of identification and data collection. This was the date used to lock the data for this manuscript, including both registration of a trial and information about ongoing trial progress. Information about trials was obtained from data linked to trial registry numbers, including registry entries, publications (if present), and review of publicly available documents such as trial protocols and statistical analysis plans.

An operational definition of a late-phase MAMS platform trial guided our search and eligibility criteria (**Table 1**). Notably, given the differences and separate considerations for different APT approaches, we limited the search to the MAMS platform design and excluded other APTs, which did not otherwise meet the definition used in this study (**Table 1**). In addition, we excluded early-phase trials, systematic reviews, commentaries, editorials, statistical methods or economic discussion manuscripts. We recorded key characteristics about MAMS platform trials including the year of registration, disease area under investigation, details about the leading organisation including the country within which they were based, number and location of recruiting countries, research phase (planned seamless phase 2/3, phase 3), trial duration, estimated sample size, number and detail of intervention arms, detail about control arms, as well as nomenclature for trials and use of the MAMS term. We prospectively registered this literature and registry-based review on PROSPERO (CRD42019153910).

Patient and public involvement

No patient involved.

For peer review only



For peer review only - http://bmjopen.bmj.com/site/about/guidelines.xhtml

## Results

Our search strategy (**Figure 1**) retrieved 399 results from which 62 trials were identified which met the definition for a late-phase APT using a MAMS master protocol design (**Table 1**).

50% (31/62) of the trials used the MAMS term specifically (**Table 2**). The 50% of trials which did not specifically use the MAMS term, instead used more generic terms such as adaptive platform, master protocol or platform trial. Of note, for the 50% of trials which used the MAMS term, this was more commonly noted if the trial was being led from the United Kingdom (UK) with 61% (19/31) of the trials using this term or from European countries (19%, 6/31). Additionally, all trials which self-identified as late-phase MAMS platform trials were being co-ordinated by a non-commercial trial organisation (100%, 31/31) (**Table S1**).

There was a pattern of gradual uptake of MAMS platform protocols over time with a rapid acceleration in more recent years, including a greater use of the MAMS approach across different disease areas and different countries (**Figure 2**). After the first registered late-phase MAMS platform trial in 2001, the design was predominantly used in trials targeting oncology and infectious diseases. However, there has been subsequent greater adoption across different disease areas, widening each year until 2020. Indeed, since 2020, there has been a rapid uptake in the use of a MAMS platform design, accelerated by COVID-19, with 28 new trial registrations in 2020 alone. (**Figure S1**). In terms of publication of trial results, most of the identified late-phase MAMS platform trials in this manuscript (76%, 47/62) had not published any primary trial results at the time of data-lock for this manuscript.



For peer review only - http://bmjopen.bmj.com/site/about/guidelines.xhtml

In terms of leading organisation, 89% (55/62) of the late-phase MAMS platform trials identified were co-ordinated by a non-commercial organisation (**Table 2**). In addition, although the MAMS design was found to be increasingly used around the world, 41% (26/62) of these late-phase trials were coordinated from the UK and 32% (20/62) from the United States of America (USA). In terms of type of late-phase design used, 53% (33/62) of late-phase trials used a seamless phase 2/3 approach from the outset, with the remainder being phase 3 clinical trials. It is important to note that some trials were registered as seamless phase 2/3 trials with the possibility for phase 3 expansion - but did not eventually proceed to phase 3.

It is clear that trials using the MAMS design were mostly co-ordinated from the USA and UK initially, but that subsequently there has been greater adoption across an increasingly large number of countries including from high-income countries (HICs) and lower and middle income countries (LMICs). There were 13 countries from which organisations were based and leading late-phase MAMS protocols overall and this had increased from 8 prior to the COVID-19 pandemic (**Figure 3A**). The number of countries recruiting to and contributing to MAMS protocols had also more than doubled from 36 prior to the COVID-19 pandemic to 75. A large number of these recruiting countries included participation of sites from LMICs even prior to the pandemic period, typically to answer research questions for infectious diseases such as tuberculosis and Ebola (**Figure 3B**).

The anticipated initial maximum sample size was estimated and demonstrated that generally late-phase MAMS platforms are large trials, typically expected to recruit



For peer review only - http://bmjopen.bmj.com/site/about/guidelines.xhtml

hundreds or even thousands of participants (**Figure S2**). There was no apparent association of original anticipated sample size with either the number of comparison arms in the initial MAMS platform, or with the number of arms later added on. However, the MAMS platform protocols registered for COVID-19 appeared to have greater estimated maximum sample sizes at the outset compared to non-COVID-19 trials. Of note, two of the MAMS platforms, RECOVERY initially starting in the UK and the WHO SOLIDARITY platform trial taking place across many countries around the world, specified no original sample size – instead stating that they would aim to recruit as many participants as possible to allow research questions to be answered. In both these trials, sample sizes were subsequently estimated based on interim analyses during the course of the trials.

The two most common areas for investigation using a late-phase MAMS protocol were infectious diseases (52%, 32/62) and cancer (29%, 18/62), with the infectious disease MAMS platform trials predominantly driven by those assessing potential therapeutics for COVID-19 (**Table S1**). All the MAMS platform protocols identified were trials of treatment interventions and 93% (58/62) of these focused on medical treatment arms only. Indeed, the vast majority of the trials assessing medical treatments within a MAMS platform were focused on drug medications (95%, 55/58), and comparatively fewer (5%, 3/58) on interventions such as radiotherapy, oxygen or respiratory ventilation strategies (**Table S2**). 5% (3/62) of the total, identified MAMS platform trials contained surgical intervention arms and to date there was only one trial assessing psychiatric interventions. Given the growing academic interest on using repurposed medications for RCTs, we noted that 45% (28/62) of the MAMS platforms had at least



For peer review only - http://bmjopen.bmj.com/site/about/guidelines.xhtml

one or more repurposed medication as a comparison arm, and indeed 35% (22/62) of the trials identified were studying only repurposed medications (**Table 3**).

As highlighted above, the ability to add in new intervention arms is often regarded as a major efficiency advantage of MAMS trials. Despite 90% (56/62) of late-phase MAMS protocols pre-specifying the intention or ability to add in new intervention arms into the platform trial at a later timepoint, to date, only 11% (7/62) of the trials included have actually added new intervention arms into an ongoing protocol (**Table S3**). Although this finding should be considered in the context that most of the late-phase MAMS trials being examined, have been registered in last few years and still waiting for maturity, with regards trial recruitment and conduct (**Figure 2**).



For peer review only - http://bmjopen.bmj.com/site/about/guidelines.xhtml

## Discussion

The MAMS platform protocol approach offers considerable advantages for clinical trial efficiency compared to more traditional designs. This is the first work, to date, to examine uptake of the MAMS approach in the late-phase trial setting and these findings should enable trial teams to better prepare, plan and deliver future MAMS platform protocols.

## Nomenclature of MAMS platform protocols

It has been recognised that APTs including those using a MAMS approach are typically delivered from a single, master protocol.[25] Although there have been attempts to clarify and harmonise terms,[26,27] it is clear that there are many mixed definitions and heterogenous reporting in the clinical trials literature. Given that only 50% (31/62) of the MAMS platform trials identified used the MAMS term specifically, does illustrate that the MAMS terminology has not been universally adopted. The reasons for this are not entirely clear but may suggest a need for more knowledge-exchange and international collaboration from teams leading late-phase MAMS platform protocols. Regardless of the exact terminology used, these trials were all deemed to be of a similar nature of design, by virtue of meeting the definition outlined in **Table 1**. As a future research priority, we support the harmonisation of terms across the clinical trials field and highlight the ongoing and commendable work from initiatives such as the European Union patient centric clinical trial platforms (EU-PEARL) in this regard (www.eu-pearl.eu).

## Delivering late-phase MAMS platform protocols



For peer review only - http://bmjopen.bmj.com/site/about/guidelines.xhtml

There are likely to be multiple factors influencing the pattern of uptake of MAMS platform protocols. One key initial factor is likely to have been previous limited awareness of this trial design. The first practically implemented late-phase MAMS platform trial was the ICON 5 phase 3 trial in ovarian cancer, registered in 2001.[28] Recruitment occurred much more rapidly than anticipated and recruitment to all the intervention arms was stopped at the single, planned, formal interim analysis. The ICON5 trial demonstrated the merits of obtaining faster answers to allow stopping of recruitment to insufficiently interesting intervention arms.[13] However, as this trial did not proceed to a subsequent stage, it was unable to demonstrate some of the many later efficiencies of a MAMS design.[28]

Subsequently, the STAMPEDE seamless phase 2/3 trial in prostate cancer, launched in 2005 and many of the features of this trial have come to define expectations and understanding of the MAMS platform protocol.[29] STAMPEDE initially had five comparison arms compared against standard of care and is currently the longest running MAMS platform trial in the world.[30] Following these two initial applications, uptake of the MAMS design in late-phase trials was initially slow. However, we have shown that there has been a rapid increase in recent years, with an undoubted acceleration in uptake to answer research questions for the COVID-19 pandemic and obtain the fast answers required in a pandemic setting.[31,32] It is important to note though that although the MAMS platform has been accelerated by application to COVID-19, the vast majority of clinical trials conducted for COVID-19 did not use efficient trial designs such as the MAMS approach.[33] In fact, the majority of COVID-19 trials consisted of multiple, separate, individual and competing registrations, often recruiting small numbers of patients, accordingly being underpowered, and ultimately



For peer review only - http://bmjopen.bmj.com/site/about/guidelines.xhtml

failing to answer research questions for the population that they aimed to serve.[34] This illustrates the importance of increasing both knowledge and uptake for more efficient trial designs such as the MAMS platform,[35] in order to provide faster answers for patients and the public.[31]

We have demonstrated that 89% of late-phase MAMS trials have been led from non-commercial organisations such as universities, hospitals and academic clinical trials units. This is likely helped by the fact that organisations which are not bound to any single treatment or intervention, are able to run platforms and engage with collaborators to contribute a number of different interventions into the MAMS platform.

Despite the finding that MAMS platforms are typically led from non-commercial organisations, we believe that it is important to highlight the multiple sources of funding required to setup and deliver a late-phase MAMS platform protocols, often with support from many commercial partners. Indeed, without this collaboration and support, it would perhaps be impossible to deliver late-phase MAMS platform trials in most disease areas. Increasingly it is clear that, whether being co-ordinated and sponsored from commercial or non-commercial organisations, there are important practical considerations for trial teams and researchers, designing and delivering a late-phase MAMS trial.[30,36,37] Indeed some of the many statistical, practical, and regulatory challenges may have contributed to the relatively low number of late-phase MAMS platform trials that have added new intervention comparison arms to date.[38–41] Accordingly, we advise that planning, setup and delivery of such trials should be through collaboration with organisations who have prior experience of delivering large, complex, late-phase RCTs.



For peer review only - http://bmjopen.bmj.com/site/about/guidelines.xhtml

We have demonstrated that the majority of late-phase MAMS protocols have historically been led from HICs. However, with increasing recruitment and participation in MAMS platforms from around the world, there clearly needs to be a focus on widening leadership and co-ordination to LMICs. We also noted that the majority of the early MAMS trials were based in one country for recruitment. This is perhaps not surprising given the often stark differences in regulatory oversight,[42] ethical approvals, as well as research capacity and capability between countries, often resulting in difficulty setting up MAMS platforms in multiple countries.[43] Indeed in a platform trial where regular adaptations are made, this can be even more difficulty as amendments to the protocol have to be implemented at pace across participating sites, following adaptations to the trial protocol.[29,44]

**Strengths and limitations**

This study documents the uptake of the MAMS platform approach in late-phase RCTs over the past 20 years and builds on previous publications which have examined the uptake of adaptive trial designs. Prior publications have examined the uptake of adaptive trials designs in general without specific focus on any design,[18,21] without distinction between early and late-phase trials,[22,23] or by focusing only on published trial results.[24] Although many of these articles do also include a small number of MAMS platform trials - due to the methods used in these historical articles, many ongoing MAMS platform trials, including recently registered trials, were not captured. Accordingly, this study reports a much wider uptake of the MAMS platform design than previously suggested, across a large number of countries and many, different disease areas. By conducting an extensive trial registry review and including all the most recent

For peer review only

trial registrations, we are able to clearly demonstrate how the COVID-19 pandemic has accelerated knowledge, use and familiarity with MAMS platform trial design and across the late-phase RCT setting.

One caveat of this current study is that most of the identified late-phase MAMS platform trials in this manuscript (76%, 47/62) have not published primary trial results to date (**Figure 2**). This is likely to represent a need for time to achieve data maturity, and highlights that comparing across platforms and drawing too many conclusions at this stage may be premature. There may also be further MAMS platform trials globally which have not been entered into a trial registry, however we would highlight the importance and globally accepted good practice of registration of all late-phase RCTs onto trial registries. The search strategy used did not include phase 1 or phase 2-only MAMS platform trials, however given the quite different considerations between early-phase exploratory and late-phase confirmatory RCTs, we felt it was appropriate to focus only on the late-phase trial setting. We have underscored the differences in terms and nomenclature used across the clinical trials field, and that some of the APTs identified may not have labelled themselves as a MAMS platform trial. In addition, other trials that some may consider similar in design, may not have been included as they did not meet the MAMS definition outlined in this manuscript (**Table 1**). It is important to note that this is a fast-moving area and there will have been further trial registrations since the April 2021 coverage in this manuscript. The information presented about included trials was accurate and up-to-date at the time of writing. However, MAMS trials given their adaptive nature, will likely have been further modified, including changes to the number of intervention arms, locations of recruitment and/or information about trial reporting. Nevertheless, we believe that



For peer review only - http://bmjopen.bmj.com/site/about/guidelines.xhtml

reporting on the uptake and characteristics across these currently registered MAMS platform trials, enables detailed consideration and better planning for teams considering future use of these efficient designs.

**Considerations for the future**

We have highlighted many reasons that trial teams may consider using a MAMS platform approach for late-phase RCTs.[16] However, it is equally important to understand that these designs are not appropriate to use in all instances of late-phase RCTs.[45,46] Nevertheless, it is clear that the number of MAMS platform protocols used in the late-phase trial setting will continue to grow. There have been a few examples of narratives for implementation from teams which have conducted late-phase MAMS platform trials.[30,36,37,47] We believe these experiences are very helpful to guide other teams looking to develop and deliver late-phase MAMS platform protocols, as well as providing an understanding for some of the regulatory, legislative and financial challenges that teams might encounter.[43] To date, published reports of experiences from trial teams delivering MAMS platform trials, remain limited. Going forward, we would urge for more experiences of implementation to be shared by each of the respective trial teams. As these designs become more widely deployed, future work could and should explore how best to further maximise efficiencies of a MAMS platform trial.

We are aware of multiple further MAMS trials that are currently being considered or designed for the late-phase setting including, but not limited to, multiple sclerosis,[48] Parkinson’s disease,[49] human immunodeficiency virus.[50] We note that to date, there has been a lack of use for this design in areas such as cardiology. However,



For peer review only - http://bmjopen.bmj.com/site/about/guidelines.xhtml

given the number of potential new interventions, large population sizes, and ongoing medical needs in the field of cardiology, it is likely that there would be many benefits from using a MAMS design in the late-phase setting for cardiovascular conditions. In truth, no matter which disease area is being considered, we would highlight the importance of collaboration and these more efficient trial approaches being championed by key opinions leaders, funders, charities and patient groups in their respective disease areas.

## Conclusions

Since the first practically implemented late-phase MAMS platform trial, there was an initial slow uptake of this design. However, in more recent years there has been a rapid increase with exponential uptake, and the MAMS design is now being used across a range of countries, disease areas and specialities. However, it is important to be aware that one size does not fit all and that each disease area, region and trial protocol will have their own considerations.

The use and knowledge of the MAMS design has undoubtedly been accelerated by applications in the COVID-19 pandemic, where they have been used with great success to get the fast answers needed in a pandemic. It is important to be aware of practical challenges to design, initiate and undertake these large-scale protocols and the need for collaboration across many partners as well as highlighting the need for more practical guidance. Once initiated though, it is clear that practical issues of late-phase MAMS protocols can be overcome and help drive substantial improvements in patient care. This analysis highlights the potential scope of MAMS platform trials

For peer review only



For peer review only - http://bmjopen.bmj.com/site/about/guidelines.xhtml

across medicine and drug development programs and may assist research teams considering use of this approach.



For peer review only - http://bmjopen.bmj.com/site/about/guidelines.xhtml

### Acknowledgements

None.

### Funding

NMN is supported by a Medical Research Council PhD Studentship [MC_UU_171339]. The funders had no role in study design, data collection and analysis, decision to publish, or preparation of the manuscript.

### Conflicts of Interest

NMN, SBL, TI, RK, and MKBP declare no conflicts of interests relevant to this work. MRS reports grants and non-financial support from Astellas, grants from Clovis, grants and non-financial support from Janssen, grants and non-financial support from Novartis, grants and non-financial support from Pfizer, grants and non-financial support from Sanofi, during the conduct of the study; personal fees from Lilly Oncology, personal fees from Janssen, all outside of the submitted work.

### Ethics approval

Not applicable. This manuscript reports findings from a trial-registry review performed with no research performed on human participants or animal subjects as a result of this trial-registry review.

### Author Contributions

NMN, SBL, TI, RK, MKBP, and MRS, wrote and approved the manuscript. NMN, SBL, RK, and MRS, designed the research. NMN performed the research search and extraction. NMN and MRS analysed the data.



For peer review only - http://bmjopen.bmj.com/site/about/guidelines.xhtml

## References

1. Collins R, Bowman L, Landray M, Peto R. The magic of randomization versus the myth of real-world evidence. N Engl J Med. 2020;382: 674–678. doi:10.1056/NEJMsb1901642
2. Bothwell LE, Greene JA, Podolsky SH, Jones DS, Malina D. Assessing the gold standard - Lessons from the history of RCTs. N Engl J Med. 2016;374: 2175–2181. doi:10.1056/NEJMms1604593
3. Zwierzyna M, Davies M, Hingorani AD, Hunter J. Clinical trial design and dissemination: Comprehensive analysis of clinicaltrials.gov and PubMed data since 2005. BMJ. 2018;361: k2130. doi:10.1136/bmj.k2130
4. DiMasi JA, Grabowski HG, Hansen RW. The Cost of Drug Development. N Engl J Med. 2015;372: 1972. doi:10.1056/nejmc1504317
5. Djulbegovic B, Kumar A, Soares HP, Hozo I, Bepler G, Clarke M, et al. Treatment success in cancer: New cancer treatment successes identified in phase 3 randomized controlled trials conducted by the National Cancer Institute-Sponsored Cooperative Oncology Groups, 1955 to 2006. Arch Intern Med. 2008;168: 632–642. doi:10.1001/archinte.168.6.632
6. Djulbegovic B, Kumar A, Glasziou P, Miladinovic B, Chalmers I. Medical research: Trial unpredictability yields predictable therapy gains. Nature. 2013;500: 395–396. doi:10.1038/500395a
7. Pocock SJ, Stone GW. The Primary Outcome Is Positive — Is That Good Enough? N Engl J Med. 2016;375: 971–979. doi:10.1056/nejmra1601511
8. Pocock SJ, Stone GW. The Primary Outcome Fails — What Next? N Engl J Med. 2016;375: 861–870. doi:10.1056/nejmra1510064
9. Nass SJ, Balogh E, Mendelsohn J. A national cancer clinical trials network:

For peer review only

Recommendations from the Institute of Medicine. Am J Ther. 2011;18: 382–391. doi:10.1097/MJT.0b013e3181ff7e23

10. Parmar MKB, Carpenter J, Sydes MR. More multiarm randomised trials of superiority are needed. Lancet. 2014;384: 283–284. doi:10.1016/S0140-6736(14)61122-3

11. Blagden SP, Billingham L, Brown LC, Buckland SW, Cooper AM, Ellis S, et al. Effective delivery of Complex Innovative Design (CID) cancer trials—A consensus statement. Br J Cancer. 2020;122: 473–482. doi:10.1038/s41416-019-0653-9

12. Royston P, Parmar MKB, Qian W. Novel designs for multi-arm clinical trials with survival outcomes with an application in ovarian cancer. Stat Med. 2003;22: 2239–2256. doi:10.1002/sim.1430

13. Parmar MKB, Barthel FMS, Sydes M, Langley R, Kaplan R, Eisenhauer E, et al. Speeding up the evaluation of new agents in cancer. J Natl Cancer Inst. 2008;100: 1204–1214. doi:10.1093/jnci/djn267

14. Bhatt DL, Mehta C. Adaptive Designs for Clinical Trials. N Engl J Med. 2016;375: 65–74. doi:10.1056/NEJMra1510061

15. Burnett T, Mozgunov P, Pallmann P, Villar SS, Wheeler GM, Jaki T. Adding flexibility to clinical trial designs: an example-based guide to the practical use of adaptive designs. BMC Med. 2020;18: 352. doi:10.1186/s12916-020-01808-2

16. Pallmann P, Bedding AW, Choodari-Oskooei B, Dimairo M, Flight L, Hampson L V., et al. Adaptive designs in clinical trials: Why use them, and how to run and report them. BMC Med. 2018;16: 29. doi:10.1186/s12916-018-1017-7

17. Dimairo M, Julious SA, Todd S, Nicholl JP, Boote J. Cross-sector surveys

For peer review only

assessing perceptions of key stakeholders towards barriers, concerns and facilitators to the appropriate use of adaptive designs in confirmatory trials. Trials. 2015;16. doi:10.1186/s13063-015-1119-x

18. Hatfield I, Allison A, Flight L, Julious SA, Dimairo M. Adaptive designs undertaken in clinical research: A review of registered clinical trials. Trials. 2016;17: 150. doi:10.1186/s13063-016-1273-9

19. Bewtra M, Lichtenstein GR. Mongersen and Smad-7 inhibition, not a Lucky 7 for patients with IBD: When trial design is as important as disease therapy. Am J Gastroenterol. 2020;115: 687–688. doi:10.14309/ajg.0000000000000564

20. Fogel DB. Factors associated with clinical trials that fail and opportunities for improving the likelihood of success: A review. Contemp Clin Trials Commun. 2018;11: 156–164.

21. Bothwell LE, Avorn J, Khan NF, Kesselheim AS. Adaptive design clinical trials: A review of the literature and ClinicalTrials.gov. BMJ Open. 2018;8: e018320. doi:10.1136/bmjopen-2017-018320

22. Collignon O, Koenig F, Koch A, Hemmings RJ, Pétavy F, Saint-Raymond A, et al. Adaptive designs in clinical trials: From scientific advice to marketing authorisation to the European Medicine Agency. Trials. 2018;19: 642. doi:10.1186/s13063-018-3012-x

23. Park JJH, Siden E, Zoratti MJ, Dron L, Harari O, Singer J, et al. Systematic review of basket trials, umbrella trials, and platform trials: A landscape analysis of master protocols. Trials. 2019;20: 572. doi:10.1186/s13063-019-3664-1

24. Sato A, Shimura M, Gosho M. Practical characteristics of adaptive design in phase 2 and 3 clinical trials. J Clin Pharm Ther. 2018;43: 170–180. doi:10.1111/jcpt.12617

For peer review only

25. Redman MW, Allegra CJ. The master protocol concept. Semin Oncol. 2016;42: 724–730. doi:10.1053/j.seminoncol.2015.07.009

26. Woodcock J, LaVange LM. Master protocols to study multiple therapies, multiple diseases, or both. N Engl J Med. 2017;377: 62–70. doi:10.1056/NEJMra1510062

27. Berry DA. The Brave New World of clinical cancer research: Adaptive biomarker-driven trials integrating clinical practice with clinical research. Mol Oncol. 2015;9: 951–959. doi:10.1016/j.molonc.2015.02.011

28. Bookman MA, Brady MF, McGuire WP, Harper PG, Alberts DS, Friedlander M, et al. Evaluation of new platinum-based treatment regimens in advanced-stage ovarian cancer: A phase III trial of the gynecologic cancer intergroup. J Clin Oncol. 2009;27: 1419–1425. doi:10.1200/JCO.2008.19.1684

29. Sydes MR, Parmar MKB, Mason MD, Clarke NW, Amos C, Anderson J, et al. Flexible trial design in practice - stopping arms for lack-of-benefit and adding research arms mid-trial in STAMPEDE: a multi-arm multi-stage randomized controlled trial. Trials. 2012;13: 168. doi:10.1186/1745-6215-13-168

30. Morrell L, Hordern J, Brown L, Sydes MR, Amos CL, Kaplan RS, et al. Mind the gap? the platform trial as a working environment. Trials. 2019;20: 297. doi:10.1186/s13063-019-3377-5

31. Noor NM, Pett SL, Esmail H, Crook AM, Vale CL, Sydes MR, et al. Adaptive platform trials using multi-arm, multi-stage protocols: getting fast answers in pandemic settings. F1000Research. 2020;9: 1109. doi:10.12688/f1000research.26253.2

32. Collignon O, Burman CF, Posch M, Schiel A. Collaborative Platform Trials to Fight COVID-19: Methodological and Regulatory Considerations for a Better



For peer review only - http://bmjopen.bmj.com/site/about/guidelines.xhtml

Societal Outcome. Clin Pharmacol Ther. 2021. doi:10.1002/cpt.2183

33. Dillman A, Zoratti MJ, Park JJH, Hsu G, Dron L, Smith G, et al. The landscape of emerging randomized clinical trial evidence for COVID-19 disease stages: A systematic review of global trial registries. Infect Drug Resist. 2020;13: 4577–4587. doi:10.2147/IDR.S288399

34. Bugin K, Woodcock J. Trends in COVID-19 therapeutic clinical trials. Nat Rev Drug Discov. 2021;20: 254–255. doi:10.1038/d41573-021-00037-3

35. Park JJ, Mogg R, Smith GE, Nakimuli-Mpungu E, Jehan F, Rayner CR, et al. How COVID-19 has fundamentally changed clinical research in global health. Lancet Glob Heal. 2021;9: e711-720.

36. Hague D, Townsend S, Masters L, Rauchenberger M, Van Looy N, Diaz-Montana C, et al. Changing platforms without stopping the train: Experiences of data management and data management systems when adapting platform protocols by adding and closing comparisons. Trials. 2019;20: 294. doi:10.1186/s13063-019-3322-7

37. Schiavone F, Bathia R, Letchemanan K, Masters L, Amos C, Bara A, et al. This is a platform alteration: A trial management perspective on the operational aspects of adaptive and platform and umbrella protocols. Trials. 2019;20: 264. doi:10.1186/s13063-019-3216-8

38. Lee KM, Brown LC, Jaki T, Stallard N, Wason J. Statistical consideration when adding new arms to ongoing clinical trials: the potentials and the caveats. Trials. 2021;22: 203. doi:10.1186/s13063-021-05150-7

39. Lee KM, Wason J, Stallard N. To add or not to add a new treatment arm to a multiarm study: A decision-theoretic framework. Stat Med. 2019;38: 3305–3321. doi:10.1002/sim.8194



For peer review only - http://bmjopen.bmj.com/site/about/guidelines.xhtml

40. Choodari-Oskooei B, Bratton DJ, Gannon MR, Meade AM, Sydes MR, Parmar MKB. Adding new experimental arms to randomised clinical trials: Impact on error rates. Clin Trials. 2020;17: 273–284. doi:10.1177/1740774520904346

41. Bennett M, Mander AP. Designs for adding a treatment arm to an ongoing clinical trial. Trials. 2020;21: 251. doi:10.1186/s13063-020-4073-1

42. Collignon O, Gartner C, Haidich AB, James Hemmings R, Hofner B, Pétavy F, et al. Current Statistical Considerations and Regulatory Perspectives on the Planning of Confirmatory Basket, Umbrella, and Platform Trials. Clin Pharmacol Ther. 2020;107: 1059–1067. doi:10.1002/cpt.1804

43. Sydes MR, Egawa S, Sanders K, Amos C, Clarke N, Kimura T, et al. Reflections on attempted Anglo-Japanese collaboration on STAMPEDE: A randomized controlled trial for men with prostate cancer. Int J Urol. 2011;18: 553–554. doi:10.1111/j.1442-2042.2011.02783.x

44. Parmar MKB, Sydes MR, Cafferty FH, Choodari-Oskooei B, Langley RE, Brown L, et al. Testing many treatments within a single protocol over 10 years at MRC Clinical Trials Unit at UCL: Multi-arm, multi-stage platform, umbrella and basket protocols. Clin Trials. 2017;14: 451–461. doi:10.1177/1740774517725697

45. Wason JMS, Brocklehurst P, Yap C. When to keep it simple - Adaptive designs are not always useful. BMC Med. 2019;17: 152. doi:10.1186/s12916-019-1391-9

46. Bretz F, Gallo P, Maurer W. Adaptive designs: The Swiss Army knife among clinical trial designs? Clin Trials. 2017;14: 417–424. doi:10.1177/1740774517699406

47. Mills SM, Mallmann J, Santacruz AM, Fuqua A, Carril M, Aisen PS, et al.

For peer review only

Preclinical trials in autosomal dominant AD: Implementation of the DIAN-TU trial. Rev Neurol (Paris). 2013;169: 737–743. doi:10.1016/j.neurol.2013.07.017

48. Fox RJ, Chataway J. Advancing trial design in progressive multiple sclerosis. Mult Scler. 2017;23: 1573–1578. doi:10.1177/1352458517729768

49. Zeissler ML, Li V, Parmar MKB, Carroll CB. Is It Possible to Conduct a Multi-Arm Multi-Stage Platform Trial in Parkinson's Disease: Lessons Learned from Other Neurodegenerative Disorders and Cancer. J Parkinsons Dis. 2020;10: 413–428. doi:10.3233/JPD-191856

50. Moore CL, Stöhr W, Crook AM, Richert L, Leliévre JD, Pantaleo G, et al. Multi-arm, multi-stage randomised controlled trials for evaluating therapeutic HIV cure interventions. Lancet HIV. 2019;6: e334-340. doi:10.1016/S2352-3018(19)30082-7

For peer review only



For peer review only - http://bmjopen.bmj.com/site/about/guidelines.xhtml

# Figure Legends

**Figure 1 – Search strategy and inclusion of late-phase MAMS platform trials.**

This flow diagram represents the search criteria and results for late-phase MAMS platform trials identified and eligible for inclusion in this review. The date of lock for new trial registrations was 1 April 2021.

**Figure 2 – Increasing uptake of late-phase trials using the MAMS platform approach over time.**

Hosepipe plot demonstrating increasing uptake of MAMS platform for late-phase clinical trials over time, accelerated by uptake during the COVID-19 pandemic. The date of lock for new trial registrations was 1 April 2021.

**Figure 3 – Countries involved in late-phase APTs using the MAMS protocol approach.**

**(A)** Country where organisations based around the world which were leading late-phase MAMS platform trials in the pre-COVID-19 era and since the COVID-19 pandemic. **(B)** Countries around the world recruiting to late-phase MAMS platform trials in the pre-COVID-19 era and since the COVID-19 pandemic.

**Table 1 – Definition for late-phase adaptive platform trial using a MAMS protocol approach.**

Given the wide-ranging definitions and terms used in the clinical trials field, this was the definition used for eligibility and inclusion in this registry-based review.



For peer review only - http://bmjopen.bmj.com/site/about/guidelines.xhtml

**Table 2 – Characteristics of included trials using a MAMS protocol approach.**

Presentation of key characteristics regarding conduct of the trial including: review of disease areas, use of the MAMS term, type of late-phase trial, type of organisation leading the trial and the country of origin of the leading organisation.

**Table 3 – Characteristics of interventions and control groups for late-phase adaptive platform trial using a MAMS protocol approach.**

Presentation of key characteristics regarding intervention and control arms in the trial including: type of intervention, repurposing of treatments, addition of new intervention arms, and type of control arm used in the MAMS platform.

For peer review only

# Supplementary information

**Figure S1. Uptake of MAMS platform protocol registrations for late-phase trials prior to and following the COVID-19 pandemic.**

**Figure S2. Anticipated maximum sample size at outset of late-phase MAMS platform trials.**

**Table S1. Characteristics by individual trial for the design, co-ordination and recruitment of late-phase MAMS platform protocols.**

**Table S2. Characteristics for trial sponsors and interventions assessed in late-phase MAMS platform trials.**

**Table S3. Characteristics for intervention arms, control group and anticipated initial maximum sample size across late-phase MAMS platform trials.**

For peer review only



For peer review only - http://bmjopen.bmj.com/site/about/guidelines.xhtml

**Table 1 – Definition for late-phase adaptive platform trial using a MAMS protocol approach.**

| Late-phase, randomised-controlled trial using multi-arm, multi-stage (MAMS) protocol definition |
| --- |
| • Late-phase defined as a phase three, seamless phase two/three, or a phase two trial which intended for phase three expansion at the outset even if this expansion does not ultimately occur.<br>• Randomisation of participants.<br>• Multi-arm defined as two or more actual or intended comparison interventions. There is typically the intention to add in new intervention arms into the platform, but this would not be considered mandatory and the addition of new intervention arms may not ultimately take place.<br>• Multi-stage defined as two or more actual or intended stages with an interim analysis in between stages, with the ability to stop recruitment to intervention arms following interim analysis. |



For peer review only - http://bmjopen.bmj.com/site/about/guidelines.xhtml

**Table 2 – Characteristics of included trials using a MAMS protocol approach.**

| Category | Number of MAMS trials | Percentage of MAMS trials |
|---|---|---|
| **Disease area** | | |
| Infection | 32 | 52% |
| Cancer | 18 | 29% |
| Neurology | 4 | 6% |
| Mental Health | 2 | 3% |
| Diabetes | 2 | 3% |
| Dermatology | 1 | 2% |
| Haematology | 1 | 2% |
| Inflammation | 1 | 2% |
| Surgery | 1 | 2% |
| **MAMS term used** | | |
| Yes | 31 | 50% |
| No | 31 | 50% |
| **Phase of trial** | | |
| Seamless phase 2/3 | 33 | 53% |
| Phase 3 | 29 | 47% |
| **Organisation leading trial** | | |
| Non-commercial | 55 | 89% |
| Commercial | 7 | 11% |
| **Country of organisation leading trial** | | |
| UK | 26 | 41% |
| USA | 20 | 32% |
| France | 5 | 8% |
| Switzerland | 2 | 3% |
| Australia | 1 | 2% |
| Austria | 1 | 2% |
| Brazil | 1 | 2% |
| Canada | 1 | 2% |
| Denmark | 1 | 2% |
| Netherlands | 1 | 2% |
| Pakistan | 1 | 2% |
| Singapore | 1 | 2% |
| Spain | 1 | 2% |

For peer review only

**Table 3 – Characteristics of interventions and control groups for late-phase adaptive platform trial using a MAMS protocol approach.**

| Category | Number of MAMS trials | Percentage of MAMS trials |
|---|---|---|
| **Type of interventions** | | |
| Medical treatments only | 58 | 93% |
| Surgical treatments only | 1 | 2% |
| Medical and surgical treatments | 2 | 3% |
| Psychiatric treatments only | 1 | 2% |
| **Repurposed medications** | | |
| None | 29 | 47% |
| All repurposed medications | 22 | 35% |
| Some repurposed medications | 6 | 10% |
| Not applicable | 5 | 8% |
| **Doses of intervention being compared** | | |
| No | 57 | 92% |
| Yes | 5 | 8% |
| **Durations of intervention(s) being compared** | | |
| No | 59 | 95% |
| Yes | 3 | 5% |
| **Plan to add in intervention arm(s)** | | |
| Yes | 56 | 90% |
| No | 6 | 10% |
| **Added in intervention arm(s)** | | |
| No | 55 | 89% |
| Yes | 7 | 11% |
| **Number of intervention comparison arms at outset** | | |
| 1 | 10 | 16% |
| 2 | 20 | 32% |
| 3 | 20 | 32% |
| 4 | 8 | 13% |
| 5 | 1 | 2% |
| 6 | 0 | 0% |
| 7 | 2 | 3% |
| 8 | 1 | 2% |
| **Number of intervention comparison arms assessed** | | |
| 1 | 8 | 13% |
| 2 | 17 | 28% |
| 3 | 21 | 34% |
| 4 | 10 | 16% |
| 5 | 1 | 2% |
| 6 | 0 | 0% |
| 7 | 1 | 2% |
| 8 | 1 | 2% |
| 9 | 0 | 0% |
| 10 | 2 | 3% |

For peer review only

| Category | Number of MAMS trials | Percentage of MAMS trials |
|---|---|---|
| **Control arm(s) in the platform** | | |
| Standard of care arm only | 38 | 61% |
| Placebo arm only | 17 | 27% |
| No control group arm | 4 | 6% |
| Both placebo and standard of care arms | 3 | 5% |

For peer review only

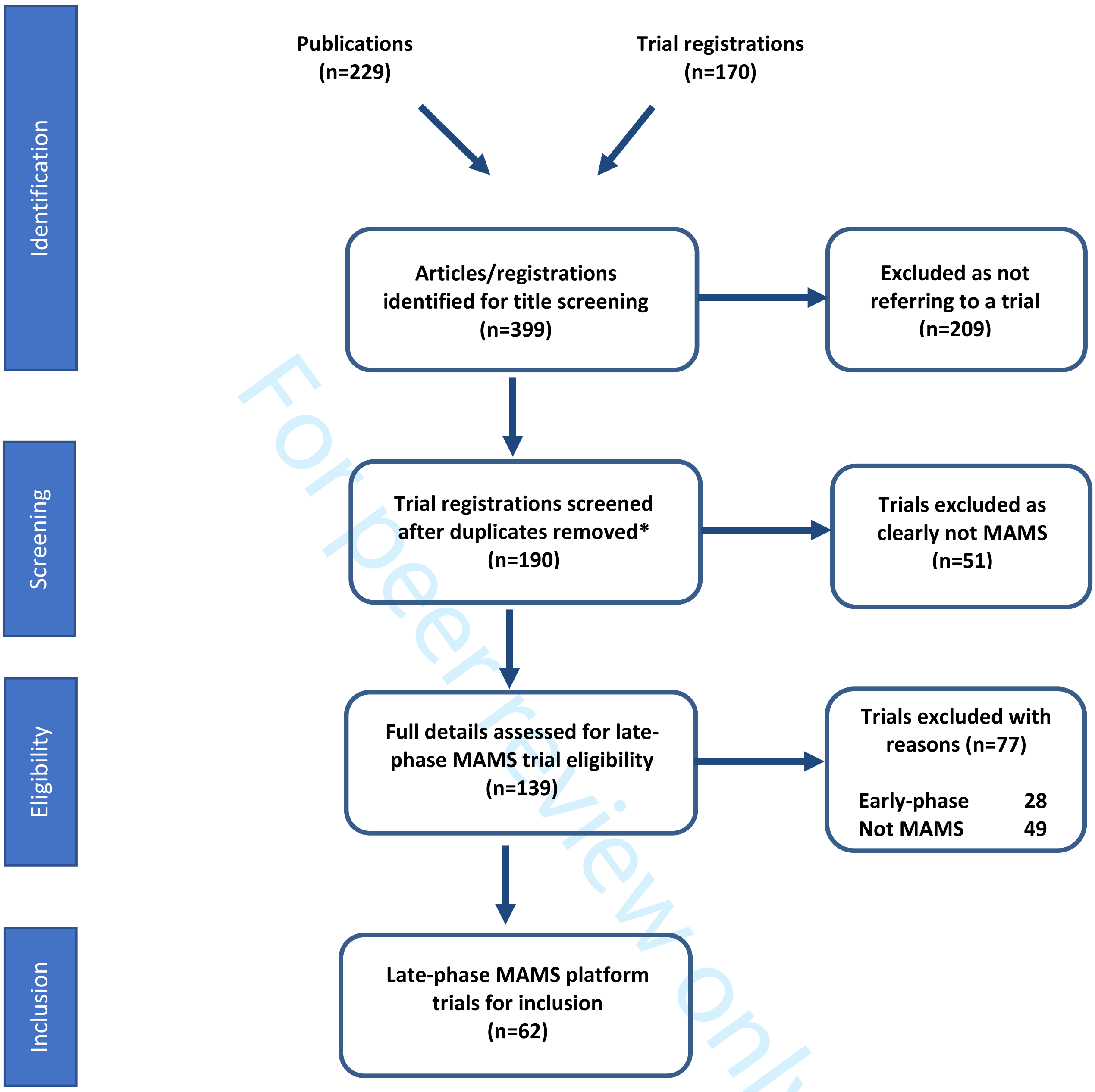


*all the identified MAMS publications provided a trial registration number in the manuscript. All the collated trial registrations then underwent screening.

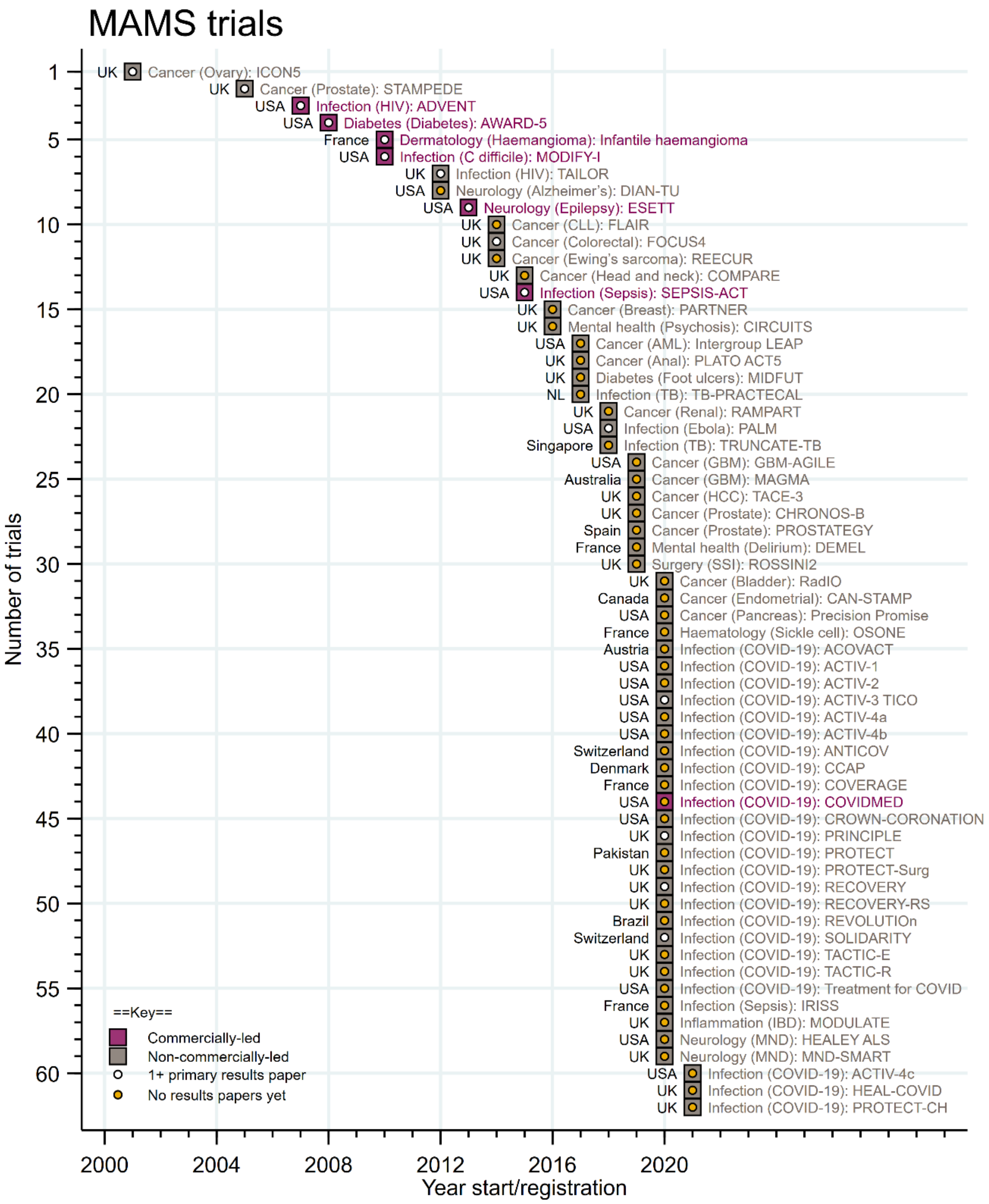
MAMS trials
Number of trials
Year start/registration
2000
2004
2008
2012
2016
2020
1
5
10
15
20
25
30
35
40
45
50
55
60
UK Cancer (Ovary): ICON5
UK Cancer (Prostate): STAMPEDE
USA Infection (HIV): ADVENT
USA Diabetes (Diabetes): AWARD-5
France Dermatology (Haemangioma): Infantile haemangioma
USA Infection (C difficile): MODIFY-I
UK Infection (HIV): TAILOR
USA Neurology (Alzheimer's): DIAN-TU
USA Neurology (Epilepsy): ESETT
UK Cancer (CLL): FLAIR
UK Cancer (Colorectal): FOCUS4
UK Cancer (Ewing's sarcoma): REECUR
UK Cancer (Head and neck): COMPARE
USA Infection (Sepsis): SEPSIS-ACT
UK Cancer (Breast): PARTNER
UK Mental health (Psychosis): CIRCUITS
USA Cancer (AML): Intergroup LEAP
UK Cancer (Anal): PLATO ACT5
UK Diabetes (Foot ulcers): MIDFUT
NL Infection (TB): TB-PRACTECAL
UK Cancer (Renal): RAMPART
USA Infection (Ebola): PALM
Singapore Infection (TB): TRUNCATE-TB
USA Cancer (GBM): GBM-AGILE
Australia Cancer (GBM): MAGMA
UK Cancer (HCC): TACE-3
UK Cancer (Prostate): CHRONOS-B
Spain Cancer (Prostate): PROSTATEGY
France Mental health (Delirium): DEMEL
UK Surgery (SSI): ROSSINI2
UK Cancer (Bladder): RadIO
Canada Cancer (Endometrial): CAN-STAMP
USA Cancer (Pancreas): Precision Promise
France Haematology (Sickle cell): OSONE
Austria Infection (COVID-19): ACOVACT
USA Infection (COVID-19): ACTIV-1
USA Infection (COVID-19): ACTIV-2
USA Infection (COVID-19): ACTIV-3 TICO
USA Infection (COVID-19): ACTIV-4a
USA Infection (COVID-19): ACTIV-4b
Switzerland Infection (COVID-19): ANTICOV
Denmark Infection (COVID-19): CCAP
France Infection (COVID-19): COVERAGE
USA Infection (COVID-19): COVIDMED
USA Infection (COVID-19): CROWN-CORONATION
UK Infection (COVID-19): PRINCIPLE
Pakistan Infection (COVID-19): PROTECT
UK Infection (COVID-19): PROTECT-Surg
UK Infection (COVID-19): RECOVERY
UK Infection (COVID-19): RECOVERY-RS
Brazil Infection (COVID-19): REVOLUTIOn
Switzerland Infection (COVID-19): SOLIDARITY
UK Infection (COVID-19): TACTIC-E
UK Infection (COVID-19): TACTIC-R
USA Infection (COVID-19): Treatment for COVID
France Infection (Sepsis): IRISS
UK Inflammation (IBD): MODULATE
USA Neurology (MND): HEALEY ALS
UK Neurology (MND): MND-SMART
USA Infection (COVID-19): ACTIV-4c
UK Infection (COVID-19): HEAL-COVID
UK Infection (COVID-19): PROTECT-CH
==Key==
Commercially-led
Non-commercially-led
1+ primary results paper
No results papers yet

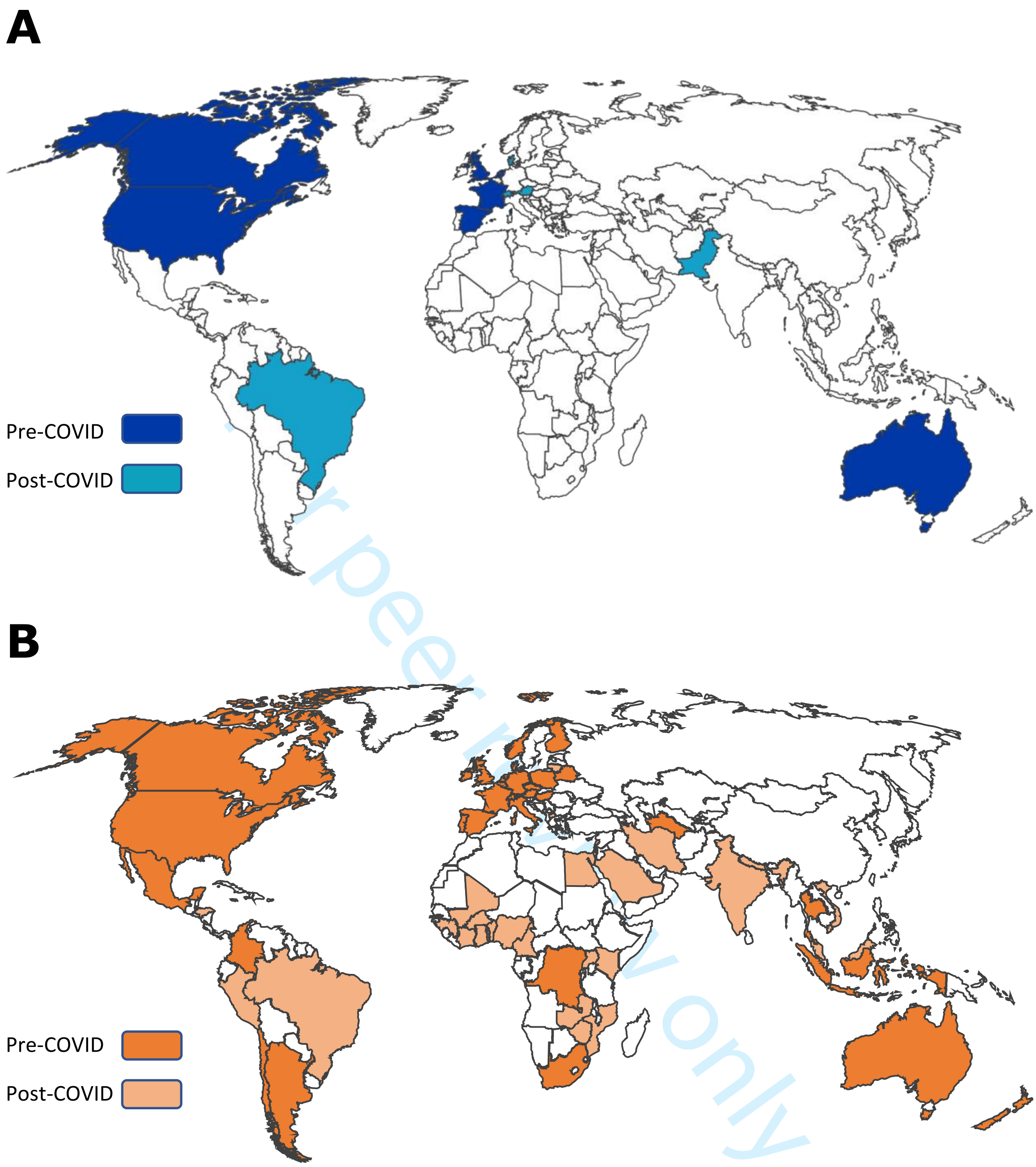
A
Pre-COVID
Post-COVID
B
Pre-COVID
Post-COVID

**Figure S1. Uptake of MAMS platform protocol registrations for late-phase trials prior to and following the COVID-19 pandemic.**

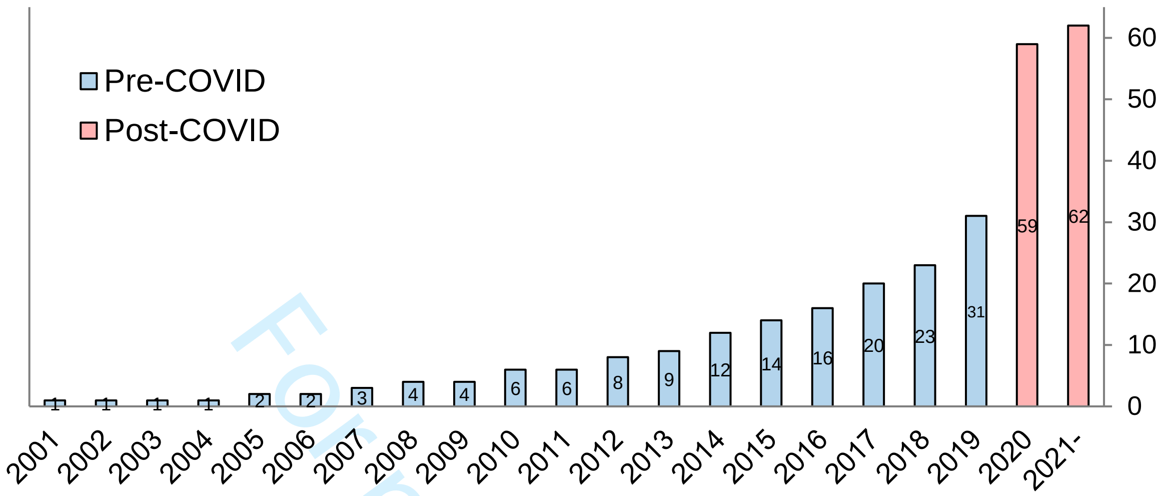


For peer review only

**Figure S2. Anticipated maximum sample size at outset of late-phase MAMS platform trials.**

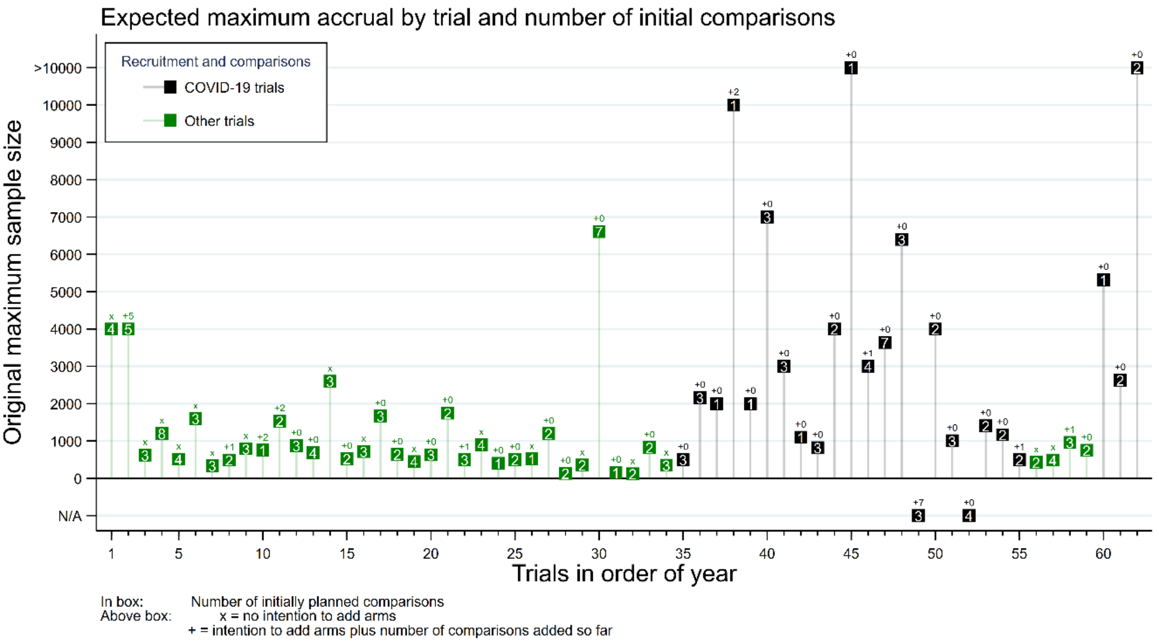


review only

**Table S1. Characteristics by individual trial for the design, co-ordination and recruitment of late-phase MAMS platform protocols.**

| Name | MAMS term used | Phase of trial | Organisation leading | Country of leading organisation | Number of recruiting countries |
|---|---|---|---|---|---|
| **2001** | | | | | |
| ICON5 | Yes | 3 | Non-commercial | UK | 1 |
| **2005** | | | | | |
| STAMPEDE | Yes | Seamless 2/3 | Non-commercial | UK | 2 |
| **2007** | | | | | |
| ADVENT | No | 3 | Commercial | USA | 1 |
| **2008** | | | | | |
| AWARD-5 | No | Seamless 2/3 | Commercial | USA | 1 |
| **2010** | | | | | |
| Infantile haemangioma | No | Seamless 2/3 | Commercial | France | 1 |
| MODIFY-I | No | 3 | Commercial | USA | 19 |
| **2012** | | | | | |
| DIAN-TU | No | Seamless 2/3 | Non-commercial | USA | 9 |
| TAILOR | Yes | Seamless 2/3 option | Non-commercial | UK | 1 |
| **2013** | | | | | |
| ESETT | No | 3 | Commercial | USA | 1 |
| **2014** | | | | | |
| FOCUS4 | Yes | Seamless 2/3 option | Non-commercial | UK | 1 |
| REECUR | Yes | Seamless 2/3 | Non-commercial | UK | 16 |
| FLAIR | No | 3 | Non-commercial | UK | 1 |
| **2015** | | | | | |
| COMPARE | Yes | 3 | Non-commercial | UK | 1 |
| SEPSIS-ACT | No | Seamless 2/3 option | Commercial | USA | 5 |
| **2016** | | | | | |
| CIRCUITS | Yes | 3 | Non-commercial | UK | 1 |
| PARTNER | No | Seamless 2/3 | Non-commercial | UK | 1 |
| **2017** | | | | | |
| Intergroup LEAP | No | Seamless 2/3 | Non-commercial | USA | 1 |
| MIDFUT | Yes | Seamless 2/3 | Non-commercial | UK | 1 |
| PLATO ACT5 | Yes | Seamless 2/3 | Non-commercial | UK | 1 |
| TB-PRACTECAL | Yes | Seamless 2/3 | Non-commercial | Netherlands | 3 |
| PALM | No | Seamless 2/3 | Non-commercial | USA | 1 |
| RAMPART | Yes | 3 | Non-commercial | UK | 5 |
| TRUNCATE-TB | Yes | Seamless 2/3 | Non-commercial | Singapore | 4 |
| **2018** | | | | | |
| CHRONOS-B | Yes | Seamless 2/3 | Non-commercial | UK | 1 |
| DEMEL | Yes | Seamless 2/3 | Non-commercial | France | 1 |
| GBM-AGILE | No | Seamless 2/3 | Non-commercial | USA | 2 |
| MAGMA | Yes | 3 | Non-commercial | Australia | 1 |
| PROSTATEGY | Yes | Seamless 2/3 | Non-commercial | Spain | 1 |
| ROSSINI2 | Yes | 3 | Non-commercial | UK | 1 |
| TACE-3 | Yes | Seamless 2/3 | Non-commercial | UK | 2 |
| **2020** | | | | | |
| CAN-STAMP | Yes | Seamless 2/3 | Non-commercial | Canada | 1 |
| HEALEY ALS | No | Seamless 2/3 | Non-commercial | USA | 1 |
| IRISS | Yes | 3 | Non-commercial | France | 1 |

| Name | MAMS term used | Phase of trial | Organisation leading | Country of leading organisation | Number of recruiting countries |
|---|---|---|---|---|---|
| MND-SMART | Yes | Seamless 2/3 | Non-commercial | UK | 1 |
| MODULATE | Yes | Seamless 2/3 | Non-commercial | UK | 1 |
| OSONE | Yes | 3 | Non-commercial | France | 1 |
| Precision Promise | No | Seamless 2/3 | Non-commercial | USA | 1 |
| RadIO | No | Seamless 2/3 | Non-commercial | UK | 1 |
| ACOVACT | No | Seamless 2/3 | Non-commercial | Austria | 1 |
| ACTIV-1 | No | 3 | Non-commercial | USA | 1 |
| ACTIV-2 | No | Seamless 2/3 | Non-commercial | USA | 1 |
| ACTIV-3 TICO | Yes | 3 | Non-commercial | USA | 5 |
| ACTIV-4a | No | 3 | Non-commercial | USA | 2 |
| ACTIV-4b | No | 3 | Non-commercial | USA | 1 |
| ANTICOV | No | 3 | Non-commercial | Switzerland | 13 |
| CCAP | No | 3 | Non-commercial | Denmark | 1 |
| COVERAGE | Yes | Seamless 2/3 | Non-commercial | France | 1 |
| COVIDMED | No | Seamless 2/3 | Commercial | USA | 1 |
| CROWN-CORONATION | No | 3 | Non-commercial | USA | 6 |
| PRINCIPLE | No | 3 | Non-commercial | UK | 1 |
| PROTECT | Yes | 3 | Non-commercial | Pakistan | 1 |
| PROTECT-Surg | Yes | 3 | Non-commercial | UK | 9 |
| RECOVERY | Yes | 3 | Non-commercial | UK | 3 |
| RECOVERY-RS | Yes | 3 | Non-commercial | UK | 1 |
| REVOLUTIOn | Yes | Seamless 2/3 | Non-commercial | Brazil | 1 |
| SOLIDARITY | No | 3 | Non-commercial | Switzerland | 30 |
| TACTIC-E | No | Seamless 2/3 | Non-commercial | UK | 1 |
| TACTIC-R | No | 3 | Non-commercial | UK | 1 |
| Treatment for COVID | No | Seamless 2/3 | Non-commercial | USA | 1 |
| **2021** | | | | | |
| ACTIV-4c | No | 3 | Non-commercial | USA | 1 |
| HEAL-COVID | No | 3 | Non-commercial | UK | 1 |
| PROTECT-CH | Yes | 3 | Non-commercial | UK | 1 |

For peer review only

**Table S2. Characteristics for trial sponsors and interventions assessed in late-phase MAMS platform trials.**

| Name | Type of interventions | Sponsor | Repurposed intervention(s) | Doses of intervention | Durations of intervention |
|---|---|---|---|---|---|
| **2001** | | | | | |
| ICON5 | Medical | Non-commercial | None | No | No |
| **2005** | | | | | |
| STAMPEDE | Medical | Non-commercial | Some | No | No |
| **2007** | | | | | |
| ADVENT | Medical | Commercial | None | Yes | No |
| **2008** | | | | | |
| AWARD-5 | Medical | Commercial | None | Yes | No |
| **2010** | | | | | |
| Infantile haemangioma | Medical | Commercial | All | Yes | No |
| MODIFY-I | Medical | Commercial | None | No | No |
| **2012** | | | | | |
| DIAN-TU | Medical | Non-commercial | None | Yes | No |
| TAILOR | Medical | Non-commercial | All | Yes | No |
| **2013** | | | | | |
| ESETT | Medical | Commercial | All | No | No |
| **2014** | | | | | |
| FOCUS4 | Medical | Non-commercial | Some | No | No |
| REECUR | Medical | Non-commercial | None | No | No |
| FLAIR | Medical | Non-commercial | None | No | No |
| **2015** | | | | | |
| COMPARE | Medical and surgical | Non-commercial | None | No | No |
| SEPSIS-ACT | Medical | Commercial | None | Yes | No |
| **2016** | | | | | |
| CIRCUITS | Psychiatric | Non-commercial | N/A | Yes | No |
| PARTNER | Medical | Non-commercial | None | No | Yes |
| **2017** | | | | | |
| Intergroup LEAP | Medical | Non-commercial | None | No | No |
| MIDFUT | Medical and surgery | Non-commercial | None | No | No |
| PLATO ACT5 | Medical | Non-commercial | N/A | Yes | No |
| TB-PRACTECAL | Medical | Non-commercial | None | No | Yes |
| **2018** | | | | | |
| PALM | Medical | Non-commercial | None | No | No |
| RAMPART | Medical | Non-commercial | None | No | No |
| TRUNCATE-TB | Medical | Non-commercial | None | No | No |
| **2019** | | | | | |
| CHRONOS-B | Medical | Non-commercial | None | No | No |
| DEMEL | Medical | Non-commercial | Yes | Yes | No |
| GBM-AGILE | Medical | Non-commercial | None | No | No |
| MAGMA | Medical | Non-commercial | None | No | Yes |
| PROSTATEGY | Medical | Non-commercial | None | No | No |
| ROSSINI2 | Surgery | Non-commercial | N/A | No | No |
| TACE-3 | Medical | Non-commercial | None | No | No |
| **2020** | | | | | |
| CAN-STAMP | Medical | Non-commercial | None | No | No |

For peer review only

| Name | Type of interventions | Sponsor | Repurposed intervention(s) | Doses of intervention | Durations of intervention |
|---|---|---|---|---|---|
| HEALEY ALS | Medical | Non-commercial | None | No | No |
| IRISS | Medical | Non-commercial | All | Yes | No |
| MND-SMART | Medical | Non-commercial | Some | No | No |
| MODULATE | Medical | Non-commercial | All | No | No |
| OSONE | Medical | Non-commercial | N/A | Yes | No |
| Precision Promise | Medical | Non-commercial | None | No | No |
| RadIO | Medical | Non-commercial | None | No | No |
| ACOVACT | Medical | Non-commercial | Some | No | No |
| ACTIV-1 | Medical | Non-commercial | All | No | No |
| ACTIV-2 | Medical | Non-commercial | None | No | No |
| ACTIV-3 TICO | Medical | Non-commercial | None | No | No |
| ACTIV-4a | Medical | Non-commercial | All | Yes | No |
| ACTIV-4b | Medical | Non-commercial | All | No | No |
| ANTICOV | Medical | Non-commercial | All | No | No |
| CCAP | Medical | Non-commercial | None | No | No |
| COVERAGE | Medical | Non-commercial | All | No | No |
| COVIDMED | Medical | Commercial | All | No | No |
| CROWN-CORONATION | Medical | Non-commercial | All | No | No |
| PRINCIPLE | Medical | Non-commercial | All | No | No |
| PROTECT | Medical | Non-commercial | All | No | No |
| PROTECT-Surg | Medical | Non-commercial | All | No | No |
| RECOVERY | Medical | Non-commercial | Some | No | No |
| RECOVERY-RS | Medical | Non-commercial | N/A | No | No |
| REVOLUTIOn | Medical | Non-commercial | All | No | No |
| SOLIDARITY | Medical | Non-commercial | Some | No | No |
| TACTIC-E | Medical | Non-commercial | None | No | No |
| TACTIC-R | Medical | Non-commercial | All | No | No |
| Treatment for COVID | Medical | Non-commercial | All | No | No |
| **2021** | | | | | |
| ACTIV-4c | Medical | Non-commercial | All | No | No |
| HEAL-COVID | Medical | Non-commercial | All | No | No |
| PROTECT-CH | Medical | Non-commercial | Yes | No | No |

For peer review only

**Table S3. Characteristics for intervention arms, control group and anticipated initial maximum sample size across late-phase MAMS platform trials.**

| Name | Number of arms at outset | Number of arms assessed in total | Plan to add arms | Actually added arms | Control group | Planned maximum sample size |
|---|---|---|---|---|---|---|
| **2001** | | | | | | |
| ICON5 | 4 | 4 | No | No | SOC | 4000 |
| **2005** | | | | | | |
| STAMPEDE | 5 | 10 | Yes | Yes | SOC | 4000 |
| **2007** | | | | | | |
| ADVENT | 3 | 3 | No | No | Placebo | 624 |
| **2008** | | | | | | |
| AWARD-5 | 8 | 8 | No | No | Placebo and SOC | 1202 |
| **2010** | | | | | | |
| Infantile haemangioma | 4 | 4 | No | No | Placebo | 512 |
| MODIFY-I | 3 | 3 | No | No | Placebo and SOC | 1600 |
| **2012** | | | | | | |
| DIAN-TU | 2 | 3 | Yes | Yes | Placebo | 490 |
| TAILOR | 3 | 3 | No | No | SOC | 336 |
| **2013** | | | | | | |
| ESETT | 3 | 3 | No | No | None | 795 |
| **2014** | | | | | | |
| FOCUS4 | 2 | 4 | Yes | Yes | Placebo | 1536 |
| REECUR | 3 | 3 | es | No | SOC | 875 |
| FLAIR | 1 | 3 | Yes | Yes | SOC | 754 |
| **2015** | | | | | | |
| COMPARE | 4 | 4 | Yes | No | SOC | 685 |
| SEPSIS-ACT | 3 | 3 | No | No | Placebo | 2600 |
| **2016** | | | | | | |
| CIRCUITS | 3 | 3 | No | No | SOC | 720 |
| PARTNER | 2 | 2 | Yes | No | SOC | 527 |
| **2017** | | | | | | |
| Intergroup LEAP | 3 | 3 | Yes | No | SOC | 1670 |
| MIDFUT | 4 | 4 | No | No | SOC | 447 |
| PLATO ACT5 | 2 | 2 | Yes | No | SOC | 640 |
| TB-PRACTECAL | 3 | 3 | Yes | No | SOC | 630 |
| PALM | 3 | 4 | Yes | Yes | None | 500 |
| RAMPART | 2 | 2 | Yes | No | SOC | 1750 |
| TRUNCATE-TB | 4 | 4 | No | No | SOC | 900 |
| **2018** | | | | | | |
| CHRONOS-B | 2 | 2 | Yes | No | SOC | 1200 |
| DEMEL | 2 | 2 | No | No | Placebo | 355 |
| GBM-AGILE | 1 | 1 | Yes | No | SOC | 400 |
| MAGMA | 2 | 2 | Yes | No | SOC | 500 |
| PROSTATEGY | 2 | 2 | Yes | No | SOC | 135 |
| ROSSINI2 | 7 | 7 | Yes | No | SOC | 6610 |
| TACE-3 | 1 | 1 | No | No | SOC | 522 |
| **2020** | | | | | | |
| CAN-STAMP | 2 | 2 | No | No | None | 120 |
| HEALEY ALS | 3 | 4 | Yes | Yes | Placebo | 960 |
| IRISS | 2 | 2 | No | No | Placebo | 429 |

| Name | Number of arms at outset | Number of arms assessed in total | Plan to add arms | Actually added arms | Control group | Planned maximum sample size |
|---|---|---|---|---|---|---|
| MND-SMART | 2 | 2 | Yes | No | Placebo | 750 |
| MODULATE | 4 | 4 | No | No | SOC | 491 |
| OSONE | 3 | 3 | No | No | SOC | 350 |
| Precision Promise | 2 | 2 | Yes | No | SOC | 825 |
| RadIO | 1 | 1 | Yes | No | SOC | 159 |
| ACOVACT | 3 | 3 | Yes | No | SOC | 500 |
| ACTIV-1 | 3 | 3 | Yes | No | SOC | 2160 |
| ACTIV-2 | 1 | 1 | Yes | No | SOC | 2000 |
| ACTIV-3 TICO | 1 | 3 | Yes | Yes | Placebo and SOC | 10000 |
| ACTIV-4a | 1 | 1 | Yes | No | SOC | 2000 |
| ACTIV-4b | 3 | 3 | Yes | No | Placebo | 7000 |
| ANTICOV | 3 | 3 | Yes | No | SOC | 3000 |
| CCAP | 1 | 1 | Yes | No | Placebo | 1100 |
| COVERAGE | 3 | 3 | Yes | No | Placebo | 820 |
| COVIDMED | 2 | 2 | Yes | No | Placebo | 4000 |
| CROWN-CORONATION | 1 | 1 | Yes | Yes | Placebo | 30000 |
| PRINCIPLE | 4 | 6 | Yes | Yes | SOC | 3000 |
| PROTECT | 7 | 7 | Yes | No | None | 3640 |
| PROTECT-Surg | 3 | 3 | Yes | No | SOC | 6400 |
| RECOVERY | 3 | 10 | Yes | Yes | SOC | N/A |
| RECOVERY-RS | 2 | 2 | Yes | No | SOC | 4000 |
| REVOLUTIOn | 3 | 3 | Yes | No | Placebo | 1005 |
| SOLIDARITY | 4 | 4 | Yes | Yes | SOC | N/A |
| TACTIC-E | 2 | 2 | Yes | No | SOC | 1407 |
| TACTIC-R | 2 | 2 | Yes | No | SOC | 1167 |
| Treatment for COVID | 2 | 3 | Yes | No | Placebo | 495 |
| **2021** | | | | | | |
| ACTIV-4c | 1 | 1 | Yes | Yes | Placebo | 5320 |
| HEAL-COVID | 2 | 2 | Yes | Yes | SOC | 2631 |
| PROTECT-CH | 2 | 2 | Yes | Yes | SOC | 12000 |

For peer review only

# PRISMA 2020 Checklist

| Section and Topic | Item # | Checklist item | Location where item is reported |
|---|---|---|---|
| **TITLE** | | | |
| Title | 1 | Identify the report as a systematic review. | 1, trial-registry review and not a formal systematic review. |
| **ABSTRACT** | | | |
| Abstract | 2 | See the PRISMA 2020 for Abstracts checklist. | 2 |
| **INTRODUCTION** | | | |
| Rationale | 3 | Describe the rationale for the review in the context of existing knowledge. | 2,5-7 |
| Objectives | 4 | Provide an explicit statement of the objective(s) or question(s) the review addresses. | 7 |
| **METHODS** | | | |
| Eligibility criteria | 5 | Specify the inclusion and exclusion criteria for the review and how studies were grouped for the syntheses. | 8-9 |
| Information sources | 6 | Specify all databases, registers, websites, organisations, reference lists and other sources searched or consulted to identify studies. Specify the date when each source was last searched or consulted. | 8 |
| Search strategy | 7 | Present the full search strategies for all databases, registers and websites, including any filters and limits used. | 8 |
| Selection process | 8 | Specify the methods used to decide whether a study met the inclusion criteria of the review, including how many reviewers screened each record and each report retrieved, whether they worked independently, and if applicable, details of automation tools used in the process. | 8-9 |
| Data collection process | 9 | Specify the methods used to collect data from reports, including how many reviewers collected data from each report, whether they worked independently, any processes for obtaining or confirming data from study investigators, and if applicable, details of automation tools used in the process. | 8-9 |
| Data items | 10a | List and define all outcomes for which data were sought. Specify whether all results that were compatible with each outcome domain in each study were sought (e.g. for all measures, time points, analyses), and if not, the methods used to decide which results to collect. | 9 |
| | 10b | List and define all other variables for which data were sought (e.g. participant and intervention characteristics, funding sources). Describe any assumptions made about any missing or unclear information. | 9 |
| Study risk of bias assessment | 11 | Specify the methods used to assess risk of bias in the included studies, including details of the tool(s) used, how many reviewers assessed each study and whether they worked independently, and if applicable, details of automation tools used in the process. | 9 |
| Effect measures | 12 | Specify for each outcome the effect measure(s) (e.g. risk ratio, mean difference) used in the synthesis or presentation of results. | Not applicable. Not formal systematic review. |
| Synthesis methods | 13a | Describe the processes used to decide which studies were eligible for each synthesis (e.g. tabulating the study intervention characteristics and comparing against the planned groups for each synthesis (item #5)). | 9 |
| | 13b | Describe any methods required to prepare the data for presentation or synthesis, such as handling of missing summary statistics, or data conversions. | 9 |
| | 13c | Describe any methods used to tabulate or visually display results of individual studies and syntheses. | 9 |
| | 13d | Describe any methods used to synthesize results and provide a rationale for the choice(s). If meta-analysis was performed, describe the | 9 |

For peer review only

# PRISMA 2020 Checklist

| Section and Topic | Item # | Checklist item | Location where item is reported |
|---|---|---|---|
| | | model(s), method(s) to identify the presence and extent of statistical heterogeneity, and software package(s) used. | |
| | 13e | Describe any methods used to explore possible causes of heterogeneity among study results (e.g. subgroup analysis, meta-regression). | Not applicable. Not formal systematic review. |
| | 13f | Describe any sensitivity analyses conducted to assess robustness of the synthesized results. | Not applicable. Not formal systematic review. |
| Reporting bias assessment | 14 | Describe any methods used to assess risk of bias due to missing results in a synthesis (arising from reporting biases). | Not applicable. Not formal systematic review. |
| Certainty assessment | 15 | Describe any methods used to assess certainty (or confidence) in the body of evidence for an outcome. | Not applicable. Not formal systematic review. |
| **RESULTS** | | | |
| Study selection | 16a | Describe the results of the search and selection process, from the number of records identified in the search to the number of studies included in the review, ideally using a flow diagram. | 10 |
| | 16b | Cite studies that might appear to meet the inclusion criteria, but which were excluded, and explain why they were excluded. | Figure 1 |
| Study characteristics | 17 | Cite each included study and present its characteristics. | Figure 2 |
| Risk of bias in studies | 18 | Present assessments of risk of bias for each included study. | Not applicable. Not formal systematic review. |
| Results of individual studies | 19 | For all outcomes, present, for each study: (a) summary statistics for each group (where appropriate) and (b) an effect estimate and its precision (e.g. confidence/credible interval), ideally using structured tables or plots. | Table 1-3 |
| Results of syntheses | 20a | For each synthesis, briefly summarise the characteristics and risk of bias among contributing studies. | Not applicable. Not formal systematic review. |
| | 20b | Present results of all statistical syntheses conducted. If meta-analysis was done, present for each the summary estimate and its precision (e.g. confidence/credible interval) and measures of statistical heterogeneity. If comparing groups, describe the direction of the effect. | Not applicable. |

For peer review only

# PRISMA 2020 Checklist

| Section and Topic | Item # | Checklist item | Location where item is reported |
|---|---|---|---|
| | | | Not formal systematic review. |
| | 20c | Present results of all investigations of possible causes of heterogeneity among study results. | Not applicable. Not formal systematic review. |
| | 20d | Present results of all sensitivity analyses conducted to assess the robustness of the synthesized results. | Not applicable. Not formal systematic review. |
| Reporting biases | 21 | Present assessments of risk of bias due to missing results (arising from reporting biases) for each synthesis assessed. | Not applicable. Not formal systematic review. |
| Certainty of evidence | 22 | Present assessments of certainty (or confidence) in the body of evidence for each outcome assessed. | Not applicable. Not formal systematic review. |
| **DISCUSSION** | | | |
| Discussion | 23a | Provide a general interpretation of the results in the context of other evidence. | 14 |
| | 23b | Discuss any limitations of the evidence included in the review. | 17-18 |
| | 23c | Discuss any limitations of the review processes used. | 17-18 |
| | 23d | Discuss implications of the results for practice, policy, and future research. | 19-20 |
| **OTHER INFORMATION** | | | |
| Registration and protocol | 24a | Provide registration information for the review, including register name and registration number, or state that the review was not registered. | 9 |
| | 24b | Indicate where the review protocol can be accessed, or state that a protocol was not prepared. | 9 |
| | 24c | Describe and explain any amendments to information provided at registration or in the protocol. | 9 |
| Support | 25 | Describe sources of financial or non-financial support for the review, and the role of the funders or sponsors in the review. | 22 |
| Competing interests | 26 | Declare any competing interests of review authors. | 22 |
| Availability of data, code and other materials | 27 | Report which of the following are publicly available and where they can be found: template data collection forms; data extracted from included studies; data used for all analyses; analytic code; any other materials used in the review. | Not applicable. All data made available as |

For peer review only

# PRISMA 2020 Checklist

| Section and Topic | Item # | Checklist item | Location where item is reported |
| --- | --- | --- | --- |
| | | | supplementary information in this manuscript submission. |

*From:* Page MJ, McKenzie JE, Bossuyt PM, Boutron I, Hoffmann TC, Mulrow CD, et al. The PRISMA 2020 statement: an updated guideline for reporting systematic reviews. BMJ 2021;372:n71. doi: 10.1136/bmj.n71

For more information, visit: http://www.prisma-statement.org/

For peer review only

BMJ Open

# Uptake of the multi-arm multi-stage (MAMS) adaptive platform approach: a trial-registry review of late-phase randomised clinical trials

| | |
|---|---|
| Journal: | *BMJ Open* |
| Manuscript ID | bmjopen-2021-055615.R1 |
| Article Type: | Original research |
| Date Submitted by the Author: | 10-Jan-2022 |
| Complete List of Authors: | Noor, Nurulamin; MRC Clinical Trials Unit at UCL<br>Love, Sharon; MRC Clinical Trials Unit at UCL<br>Isaacs, Talia; University College London, Institute of Education<br>Kaplan, Richard; MRC Clinical Trials Unit at UCL<br>Parmar, Mahesh; MRC Clinical Trials Unit at UCL<br>Sydes, Matthew; MRC Clinical Trials Unit at UCL |
| <b>Primary Subject Heading</b>: | Pharmacology and therapeutics |
| Secondary Subject Heading: | Global health, Infectious diseases, Oncology, Research methods, Public health |
| Keywords: | Clinical trials < THERAPEUTICS, PUBLIC HEALTH, INFECTIOUS DISEASES, ONCOLOGY, CLINICAL PHARMACOLOGY |
| | |

SCHOLARONE™
Manuscripts

*I, the Submitting Author has the right to grant and does grant on behalf of all authors of the Work (as defined in the below author licence), an exclusive licence and/or a non-exclusive licence for contributions from authors who are: i) UK Crown employees; ii) where BMJ has agreed a CC-BY licence shall apply, and/or iii) in accordance with the terms applicable for US Federal Government officers or employees acting as part of their official duties; on a worldwide, perpetual, irrevocable, royalty-free basis to BMJ Publishing Group Ltd ("BMJ") its licensees and where the relevant Journal is co-owned by BMJ to the co-owners of the Journal, to publish the Work in this journal and any other BMJ products and to exploit all rights, as set out in our licence.*

*The Submitting Author accepts and understands that any supply made under these terms is made by BMJ to the Submitting Author unless you are acting as an employee on behalf of your employer or a postgraduate student of an affiliated institution which is paying any applicable article publishing charge ("APC") for Open Access articles. Where the Submitting Author wishes to make the Work available on an Open Access basis (and intends to pay the relevant APC), the terms of reuse of such Open Access shall be governed by a Creative Commons licence – details of these licences and which Creative Commons licence will apply to this Work are set out in our licence referred to above.*

*Other than as permitted in any relevant BMJ Author's Self Archiving Policies, I confirm this Work has not been accepted for publication elsewhere, is not being considered for publication elsewhere and does not duplicate material already published. I confirm all authors consent to publication of this Work and authorise the granting of this licence.*

For peer review only

# Uptake of the multi-arm multi-stage (MAMS) adaptive platform approach: a trial-registry review of late-phase randomised clinical trials

Nurulamin M. Noor (0000-0003-3426-6408)[1*], Sharon B. Love (0000-0002-6695-5390)[1], Talia Isaacs (0000-0003-4302-3379)[2], Richard Kaplan (0000-0002-0189-8348)[1], Mahesh K.B. Parmar (0000-0003-0166-1700)[1], Matthew R. Sydes (0000-0002-9323-1371)[1].

[1] MRC Clinical Trials Unit at University College London, Institute of Clinical Trials and Methodology, London, United Kingdom.

[2] UCL Institute of Education, University College London, London, United Kingdom.

[*] Corresponding author: Dr Nurulamin Noor, Medical Research Council Clinical Trials Unit at University College London, London, United Kingdom

Email: noorn@doctors.org.uk

For peer review only



For peer review only - http://bmjopen.bmj.com/site/about/guidelines.xhtml

## Abstract

### Background

For medical conditions with numerous interventions worthy of investigation, there are many advantages of a multi-arm multi-stage (MAMS) platform trial approach. However, there is currently limited knowledge on uptake of the MAMS design, especially in the late-phase setting. We sought to examine uptake and characteristics of late-phase MAMS platform trials, to enable better planning for teams considering future use of this approach.

### Design

We examined uptake of registered, late-phase MAMS platforms in the EU clinical trials register, Australian New Zealand Clinical Trials Registry, International Standard Randomised Controlled Trial Number registry, Pan African Clinical Trials Registry, World Health Organisation International Clinical Trial Registry Platform, and databases: PubMed, Medline, Cochrane Library, Global Health Library, and EMBASE. Searching was performed and review data frozen on 1 April 2021. MAMS platforms were defined as requiring two or more comparison arms, with two or more trial stages, with an interim analysis allowing for stopping of recruitment to arms and typically the ability to add new intervention arms.

### Results

62 late-phase clinical trials using a MAMS approach were included. Overall, the number of late-phase trials using the MAMS design has been increasing since 2001 and been accelerated by coronavirus disease 2019 (COVID-19). The majority of current MAMS platforms were either targeting infectious diseases (52%) or cancers

For peer review only

(29%) and all identified trials were for treatment interventions. 89% (55/62) of MAMS platforms were evaluating medications, with 45% (28/62) of the MAMS platforms having at least one or more repurposed medication as a comparison arm.

**Conclusions**

Historically, late-phase trials have adhered to long-established standard (two-arm) designs. However, the number of late-phase MAMS platform trials is increasing, across a range of different disease areas. This study highlights the potential scope of MAMS platform trials and may assist research teams considering use of this approach in the late-phase RCT setting.

Keywords: multi-arm, multi-stage, MAMS, adaptive, platform, clinical trial, late-phase, master protocol, uptake, trial registry-review

For peer review only

## Strengths and limitations of this study

- This study builds on previous publications reporting on uptake of adaptive or platform trials in general, by reporting the uptake and characteristics of MAMS platform late-phase RCTs over the past 20 years.
- The search strategy used did not include phase 1 or phase 2-only MAMS platform trials, however this was because of the quite different considerations for trial teams designing and conducting early-phase exploratory compared to late-phase confirmatory RCTs.
- By conducting an extensive trial registry review and including all the most recent trial registrations, this study reports a much wider uptake of the MAMS platform design than previously suggested, across a large number of countries and different disease areas.
- It is important to note that adaptive trials by their nature are fast-moving area and there will have been further trial registrations since the April 2021 coverage in this manuscript. The information presented about included trials was accurate and up-to-date at the time of writing.
- We believe that reporting on the uptake and characteristics across these currently registered MAMS platform trials, enables detailed consideration and better planning for teams considering future use of these efficient designs.

For peer review only

## Introduction

Randomised clinical trials (RCTs) are the gold standard for investigating healthcare interventions. Late-phase RCTs in particular play an important role in shaping health policy, guidelines and informing clinical practice to ensure better outcomes for patients and the public.[1] Historically, for most medical conditions, there have been few therapeutic options and long lag-times for developing new treatments. Thus, traditional two-arm, parallel-group RCTs were appropriate to perform in many instances.[2] However, nowadays there are multiple potential interventions available for testing in many medical conditions, and with patients, clinicians, and healthcare systems demanding faster answers to clinical questions. It is clearly neither efficient nor expedient to continue with this approach of multiple, separate, individual two-arm RCTs. In addition, there are many more aspects of treatments to evaluate than ever before including: doses, durations, and combinations of interventions.

Despite the rapidly rising number of registered clinical trials being performed around the world,[3] there are growing concerns about the length of time and increasing costs of performing late-phase RCTs.[4] Whilst most late-phase RCTs do reach a conclusion, only 30-40% are able to demonstrate efficacy of an intervention, sometimes referred to as “positive” trials. [5,6] We would caution against the use of use of terms such as “positive” or “negative” and highlight the growing literature suggesting against use of these terms to describe RCT findings.[7,8] Nevertheless, these results do highlight that there is a pressing need to speed up the evaluation process in late-phase RCTs. In line with this, there has been a movement towards “faster, better, more efficient” trials.[9] Indeed, it is now well recognised that for

For peer review only

conditions for which there are multiple interventions worthy of investigation, consideration should be given to using multi-arm trial designs.[10]

A range of different trial designs have been proposed which have been classified by some under the umbrella term of complex, innovative designs (CIDs).[11] The adaptive platform trial (APT) approach using a multi-arm multi-stage (MAMS) protocol is one such design and offers many solutions to the above-described problems of traditional trial designs.[12] The MAMS platform trial typically uses a single master protocol to allow multiple primary research questions to be answered.[13] Multiple interventions can be assessed in parallel and the shared control arm allows immediate efficiency saving by reducing the overall recruitment needed. Once participants are enrolled, information accumulates during the course of the trial and adaptations can be made including whether to add in a new intervention arm or stop recruitment to an ongoing intervention arm.[14] These adaptations are typically made following interim analysis and according to predefined criteria,[15] to ensure they preserve validity and integrity of the trial. Of note, many of the MAMS-type adaptations could also be achieved using a Bayesian framework.[16] Crucially these adaptations are made via amendment to a trial protocol rather than having to register, setup and conduct a separate, new trial entirely.[17]

For peer review only

To date, the uptake of adaptive designs has been lower for late-phase RCTs,[18] compared to early-phase RCTs – where they have been used with great success.[19] There are likely many reasons for lower uptake in late-phase settings, including: greater reliance on findings from late-phase confirmatory trials for regulatory purposes including marketing authorisation of medications, as well as more limited knowledge



For peer review only - http://bmjopen.bmj.com/site/about/guidelines.xhtml

and lack of formal guidance about how best to apply these newer designs to the late-phase setting. However, given the high costs of late-phase trial programmes,[4] and the significant commercial impact from failure to demonstrate efficacy,[20] the "early get out" offered by stopping recruitment to interventions showing lack of benefit, or harm, makes the MAMS design potentially applicable across many different disease areas in the late-phase setting.[21]

Previous studies have focused on the uptake of adaptive trials in general,[19,22] across both early and late-phase settings,[23] or platform trials in general,[24] but without specific focus on the MAMS approach. Additionally due to the varied methods used, including restricting searches to trial publications,[25] many ongoing MAMS platform trials, including recently registered trials have not historically been captured. Despite anecdotal reports for increasing use of the MAMS platform design in late-phase settings, there is currently, no study documenting how extensive this uptake has been. Accordingly, we present a trial registry-based review to examine the worldwide uptake of APTs using MAMS protocols in the late-phase RCT setting.

For peer review only

## Methods

We conducted a systematic search for APTs using MAMS protocols in late-phase trial settings (**Table S1**). In particular, given the knowledge and awareness that many MAMS platform trials would be unpublished, and the recognised importance of registering clinical trials, this review focused beyond trial publications only and sought to extract data from global trial registries (**Table S1**). This enabled us to identify many further registered but unpublished trials.

All the MAMS platform trials initially identified in the published literature reported an international clinical trial registry number and could subsequently be linked to a trial registration. All late-phase MAMS platform trials included in this review were registered on a trial registry by 1 April 2021, which was the final day of identification and data collection. This was the date used to lock the data for this manuscript, including both registration of a trial and information about ongoing trial progress. Information about trials was obtained from data linked to trial registry numbers, including registry entries, publications (if present), and review of publicly available documents such as trial protocols and statistical analysis plans.

An operational definition of a late-phase MAMS platform trial guided our search and eligibility criteria (**Table 1**). Notably, given the differences and separate considerations for different APT approaches, we limited the search to the MAMS platform design and excluded other APTs, which did not otherwise meet the definition used in this study (**Table 1**). In addition, we excluded early-phase trials, systematic reviews, commentaries, editorials, statistical methods or economic discussion manuscripts. We recorded key characteristics about MAMS platform trials including the year of

For peer review only

registration, disease area under investigation, details about the leading organisation including the country within which they were based, number and location of recruiting countries, research phase (planned seamless phase 2/3, phase 3), trial duration, estimated sample size, number and detail of intervention arms, detail about control arms, as well as nomenclature for trials and use of the MAMS term. We used the PRISMA checklist as a guide for reporting of this study and prospectively registered this literature and registry-based review on PROSPERO (CRD42019153910).

Patient and public involvement

No patient involved.

## Results

Our search strategy (**Figure 1**) retrieved 399 results from which 62 trials were identified which met the definition for a late-phase APT using a MAMS master protocol design (**Table 1**).

50% (31/62) of the trials used the MAMS term specifically (**Table 2**). The 50% of trials which did not specifically use the MAMS term, instead used more generic terms such as adaptive platform, master protocol or platform trial. Of note, for the 50% of trials which used the MAMS term, this was more commonly noted if the trial was being led from the United Kingdom (UK) with 61% (19/31) of the trials using this term, or if being led from European countries (19%, 6/31). Additionally, all trials which self-identified as late-phase MAMS platform trials were being co-ordinated by a non-commercial trial organisation (100%, 31/31) (**Table S2**).



For peer review only - http://bmjopen.bmj.com/site/about/guidelines.xhtml

There was a pattern of gradual uptake of MAMS platform protocols over time with a rapid acceleration in more recent years, including a greater use of the MAMS approach across different disease areas and different countries (**Figure 2**). After the first registered late-phase MAMS platform trial in 2001, the design was predominantly used in trials targeting oncology and infectious diseases. However, there has been subsequent greater adoption across different disease areas, widening each year until 2020. Indeed, since 2020, there has been a rapid uptake in the use of a MAMS platform design, accelerated by COVID-19, with 28 new trial registrations in 2020 alone. (**Figure S1**). In terms of publication of trial results, most of the identified late-phase MAMS platform trials in this manuscript (76%, 47/62) had not published any primary trial results at the time of data-lock for this manuscript.

In terms of leading organisation, 89% (55/62) of the late-phase MAMS platform trials identified were co-ordinated by a non-commercial organisation (**Table 2**). In addition, although the MAMS design was found to be increasingly used around the world, 41% (26/62) of these late-phase trials were coordinated from the UK and 32% (20/62) from the United States of America (USA). In terms of type of late-phase design used, 53% (33/62) of late-phase trials used a seamless phase 2/3 approach from the outset, with the remainder being phase 3 clinical trials. It is important to note that some trials were registered as seamless phase 2/3 trials with the possibility for phase 3 expansion - but did not eventually proceed to phase 3.

It is clear that trials using the MAMS design were mostly co-ordinated from the USA and UK initially, but that subsequently there has been greater adoption across an increasingly large number of countries including from high-income countries (HICs)



For peer review only - http://bmjopen.bmj.com/site/about/guidelines.xhtml

and lower and middle income countries (LMICs). There were 13 countries from which organisations were based and leading late-phase MAMS protocols overall and this had increased from 8 prior to the COVID-19 pandemic (**Figure 3A**). The number of countries recruiting to and contributing to MAMS protocols had also more than doubled from 36 prior to the COVID-19 pandemic to 75. A large number of these recruiting countries included participation of sites from LMICs even prior to the pandemic period, typically to answer research questions for infectious diseases such as tuberculosis and Ebola (**Figure 3B**).

The anticipated initial maximum sample size was estimated and demonstrated that generally late-phase MAMS platforms are large trials, typically expected to recruit hundreds or even thousands of participants (**Figure S2**). There was no apparent association of original anticipated sample size with either the number of comparison arms in the initial MAMS platform, or with the number of arms later added on. However, the MAMS platform protocols registered for COVID-19 appeared to have greater estimated maximum sample sizes at the outset compared to non-COVID-19 trials. Of note, two of the MAMS platforms, RECOVERY initially starting in the UK and the WHO SOLIDARITY platform trial taking place across many countries around the world, specified no original sample size – instead stating that they would aim to recruit as many participants as possible to allow research questions to be answered. In both these trials, sample sizes were subsequently estimated based on interim analyses during the course of the trials.

The two most common areas for investigation using a late-phase MAMS protocol were infectious diseases (52%, 32/62) and cancer (29%, 18/62), with the infectious disease



For peer review only - http://bmjopen.bmj.com/site/about/guidelines.xhtml

MAMS platform trials predominantly driven by those assessing potential therapeutics for COVID-19 (**Table S2**). All the MAMS platform protocols identified were trials of treatment interventions and 93% (58/62) of these focused on medical treatment arms only. Indeed, the vast majority of the trials assessing medical treatments within a MAMS platform were focused on drug medications (95%, 55/58), and comparatively fewer (5%, 3/58) on interventions such as radiotherapy, oxygen or respiratory ventilation strategies (**Table S3**). 5% (3/62) of the total, identified MAMS platform trials contained surgical intervention arms and to date there was only one trial assessing psychiatric interventions. Given the growing academic interest on using repurposed medications for RCTs, we noted that 45% (28/62) of the MAMS platforms had at least one or more repurposed medication as a comparison arm, and indeed 35% (22/62) of the trials identified were studying only repurposed medications (**Table 3**).

As highlighted above, the ability to add in new intervention arms is often regarded as a major efficiency advantage of MAMS trials. Despite 90% (56/62) of late-phase MAMS protocols pre-specifying the intention or ability to add in new intervention arms into the platform trial at a later timepoint, to date, only 11% (7/62) of the trials included have actually added new intervention arms into an ongoing protocol (**Table S4**). Although this finding should be considered in the context that most of the late-phase MAMS trials being examined (**Table S5**), have been registered in last few years and still waiting for maturity, with regards trial recruitment and conduct (**Figure 2**).



For peer review only - http://bmjopen.bmj.com/site/about/guidelines.xhtml

## Discussion

The MAMS platform protocol approach offers considerable advantages for clinical trial efficiency compared to more traditional designs. This is the first work, to date, examining uptake of the MAMS approach in the late-phase trial setting and these findings should enable trial teams to better prepare, plan and deliver future MAMS platform protocols.

### Nomenclature of MAMS platform protocols

It has been recognised that APTs including those using a MAMS approach are typically delivered from a single, master protocol.[26] Although there have been attempts to clarify and harmonise terms,[27,28] it is clear that there are many mixed definitions and heterogenous reporting in the clinical trials literature. Given that only 50% (31/62) of the MAMS platform trials identified used the MAMS term specifically, does illustrate that the MAMS terminology has not been universally adopted. The reasons for this are not entirely clear but may suggest a need for more knowledge-exchange and international collaboration from teams leading late-phase MAMS platform protocols. Regardless of the exact terminology used, these trials were all deemed to be of a similar nature of design, by virtue of meeting the definition outlined in **Table 1**. As a future research priority, we support the harmonisation of terms across the clinical trials field and highlight the ongoing and commendable work from initiatives such as the European Union patient centric clinical trial platforms (EU-PEARL) in this regard (www.eu-pearl.eu).

### Delivering late-phase MAMS platform protocols


For peer review only - http://bmjopen.bmj.com/site/about/guidelines.xhtml

There are likely to be multiple factors influencing the pattern of uptake of MAMS platform protocols. One key initial factor is likely to have been previous limited awareness of this trial design. The first practically implemented late-phase MAMS platform trial was the ICON 5 phase 3 trial in ovarian cancer, registered in 2001.[29] Recruitment occurred much more rapidly than anticipated and recruitment to all the intervention arms was stopped at the single, planned, formal interim analysis. The ICON5 trial demonstrated the merits of obtaining faster answers to allow stopping of recruitment to insufficiently interesting intervention arms.[13] However, as this trial did not proceed to a subsequent stage, it was unable to demonstrate some of the many later efficiencies of a MAMS design.[29]

Subsequently, the STAMPEDE seamless phase 2/3 trial in prostate cancer, launched in 2005 and many of the features of this trial have come to define expectations and understanding of the MAMS platform protocol.[30] STAMPEDE initially had five comparison arms compared against standard of care and is currently the longest running MAMS platform trial in the world.[31] Following these two initial applications, uptake of the MAMS design in late-phase trials was initially slow. However, we have shown that there has been a rapid increase in recent years, with an undoubted acceleration in uptake to answer research questions for the COVID-19 pandemic and obtain the fast answers required in a pandemic setting.[32,33] It is important to note though that although the MAMS platform has been accelerated by application to COVID-19, the vast majority of clinical trials conducted for COVID-19 did not use efficient trial designs such as the MAMS approach.[34] In fact, the majority of COVID-19 trials consisted of multiple, separate, individual and competing registrations, often recruiting small numbers of patients, accordingly being underpowered, and ultimately



For peer review only - http://bmjopen.bmj.com/site/about/guidelines.xhtml

failing to answer research questions for the population that they aimed to serve.[35] This illustrates the importance of increasing both knowledge and uptake for more efficient trial designs such as the MAMS platform,[36] in order to provide faster answers for patients and the public.[32]

We have demonstrated that 89% of late-phase MAMS trials have been led from non-commercial organisations such as universities, hospitals and academic clinical trials units. This is likely helped by the fact that organisations which are not bound to any single treatment or intervention, are able to run platforms and engage with collaborators to contribute a number of different interventions into the MAMS platform.

Despite the finding that MAMS platforms are typically led from non-commercial organisations, we believe that it is important to highlight the multiple sources of funding required to setup and deliver a late-phase MAMS platform protocols, often with support from many commercial partners. Indeed, without this collaboration and support, it would perhaps be impossible to deliver late-phase MAMS platform trials in most disease areas. Increasingly it is clear that, whether being co-ordinated and sponsored from commercial or non-commercial organisations, there are important practical considerations for trial teams and researchers, designing and delivering a late-phase MAMS trial.[31,37,38] Indeed some of the many statistical, practical, and regulatory challenges may have contributed to the relatively low number of late-phase MAMS platform trials that have added new intervention comparison arms to date.[39–42] – With regards statistical considerations, the question of whether type one error control needs to be strongly controlled or not, for multiple treatment comparisons in a MAMS platform – remains an ongoing debate and has been well covered in the recent trials



For peer review only - http://bmjopen.bmj.com/site/about/guidelines.xhtml

literature.[43–46] Accordingly, we advise that planning, setup and delivery of such trials should be through collaboration with organisations who have prior experience of delivering large, complex, late-phase RCTs.

We have demonstrated that the majority of late-phase MAMS protocols have historically been led from HICs. However, with increasing recruitment and participation in MAMS platforms from around the world, there clearly needs to be a focus on widening leadership and co-ordination to LMICs. We also noted that the majority of the early MAMS trials were based in one country for recruitment. This is perhaps not surprising given the often stark differences in regulatory oversight,[47] ethical approvals, as well as research capacity and capability between countries, often resulting in difficulty setting up MAMS platforms in multiple countries.[48] Indeed in a platform trial where regular adaptations are made, this can be more challenging as amendments to the protocol have to be implemented at pace across participating sites.[30,49]

**Strengths and limitations**

This study documents the uptake of the MAMS platform approach in late-phase RCTs over the past 20 years and builds on previous publications which have examined the uptake of adaptive trial designs. Prior publications have examined the uptake of adaptive trials designs in general without specific focus on any design,[19,22] without distinction between early and late-phase trials,[23,24] or by focusing only on published trial results.[25] Although many of these articles do also include a small number of MAMS platform trials - due to the methods used in these historical articles, many ongoing MAMS platform trials, including recently registered trials, were not captured.



For peer review only - http://bmjopen.bmj.com/site/about/guidelines.xhtml

Accordingly, this study reports a much wider uptake of the MAMS platform design than previously suggested, across a large number of countries and many, different disease areas. By conducting an extensive trial registry review and including all the most recent trial registrations, we are able to clearly demonstrate how the COVID-19 pandemic has accelerated knowledge, use and familiarity with MAMS platform trial design and across the late-phase RCT setting.

One caveat of this current study is that most of the identified late-phase MAMS platform trials in this manuscript (76%, 47/62) have not published primary trial results to date (**Figure 2**). This is likely to represent a need for time to achieve data maturity, and highlights that comparing across platforms and drawing too many conclusions at this stage may be premature. There may also be further MAMS platform trials globally which have not been entered into a trial registry, however we would highlight the importance and globally accepted good practice of registration of all late-phase RCTs onto trial registries. The search strategy used did not include phase 1 or phase 2-only MAMS platform trials, however given the quite different considerations between early-phase exploratory and late-phase confirmatory RCTs, we felt it was appropriate to focus only on the late-phase trial setting. We have underscored the differences in terms and nomenclature used across the clinical trials field, and that some of the APTs identified may not have labelled themselves as a MAMS platform trial. In addition, other trials that some may consider similar in design, may not have been included as they did not meet the MAMS definition outlined in this manuscript (**Table 1**). It is important to note that this is a fast-moving area and there will have been further trial registrations since the April 2021 coverage in this manuscript. The information presented about included trials was accurate and up-to-date at the time of writing.



For peer review only - http://bmjopen.bmj.com/site/about/guidelines.xhtml

However, MAMS trials given their adaptive nature, will likely have been further modified, including changes to the number of intervention arms, locations of recruitment and/or information about trial reporting. Nevertheless, we believe that reporting on the uptake and characteristics across these currently registered MAMS platform trials, enables detailed consideration and better planning for teams considering future use of these efficient designs.

For peer review only

## Considerations for the future

We have highlighted many reasons that trial teams may consider using a MAMS platform approach for late-phase RCTs.[17] However, it is equally important to understand that these designs are not appropriate to use in all instances of late-phase RCTs.[50,51] Nevertheless, it is clear that the number of MAMS platform protocols used in the late-phase trial setting will continue to grow. There have been a few examples of narratives for implementation from teams which have conducted late-phase MAMS platform trials. In particular, there have been recent manuscripts, focusing on the many additional operational considerations, including; data management,[37] trial co-ordination,[38] as well as the overall experiences from teams developing and delivering large, late-phase MAMS platform trials.[31] We believe these experiences are very helpful to guide other teams looking to develop and deliver late-phase MAMS platform protocols, as well as providing an understanding for some of the regulatory, legislative and financial challenges that teams might encounter.[48] To date, published reports of experiences from trial teams delivering MAMS platform trials, remain limited. Going forward, we would urge for more experiences of implementation to be shared by each of the respective trial teams. As these designs



For peer review only - http://bmjopen.bmj.com/site/about/guidelines.xhtml

become more widely deployed, future work could and should explore how best to further maximise efficiencies of a MAMS platform trial.

We are aware of multiple further MAMS trials that are currently being considered or designed for the late-phase setting including, but not limited to, multiple sclerosis,[52] Parkinson's disease,[53] human immunodeficiency virus.[54] We note that to date, there has been a lack of use for this design in areas such as cardiology. However, given the number of potential new interventions, large population sizes, and ongoing medical needs in the field of cardiology, it is likely that there would be many benefits from using a MAMS design in the late-phase setting for cardiovascular conditions. In truth, no matter which disease area is being considered, we would highlight the importance of collaboration and these more efficient trial approaches being championed by key opinions leaders, funders, charities and patient groups in their respective disease areas.

## Conclusions

Since the first practically implemented late-phase MAMS platform trial, there was an initial slow uptake of this design. However, in more recent years there has been a rapid increase with exponential uptake, and the MAMS design is now being used across a range of countries, disease areas and specialities. However, it is important to be aware that one size does not fit all and that each disease area, region and trial protocol will have their own considerations.

The use and knowledge of the MAMS design has undoubtedly been accelerated by applications in the COVID-19 pandemic, where they have been used with great

For peer review only

success to get the fast answers needed in a pandemic. It is important to be aware of practical challenges to design, initiate and undertake these large-scale protocols and the need for collaboration across many partners as well as highlighting the need for more practical guidance. Once initiated though, it is clear that practical issues of late-phase MAMS protocols can be overcome and help drive substantial improvements in patient care. This analysis highlights the potential scope of MAMS platform trials across medicine and drug development programs and may assist research teams considering use of this approach.

For peer review only



For peer review only - http://bmjopen.bmj.com/site/about/guidelines.xhtml

**Acknowledgements**

None.

**Funding**

NMN is supported by a Medical Research Council PhD Studentship [MC_UU_171339]. The funders had no role in study design, data collection and analysis, decision to publish, or preparation of the manuscript.

**Conflicts of Interest**

NMN, SBL, TI, RK, and MKBP declare no conflicts of interests relevant to this work. MRS reports grants and non-financial support from Astellas, grants from Clovis, grants and non-financial support from Janssen, grants and non-financial support from Novartis, grants and non-financial support from Pfizer, grants and non-financial support from Sanofi, during the conduct of the study; personal fees from Lilly Oncology, personal fees from Janssen, all outside of the submitted work.

**Data sharing**

All data relevant to the study are included in the article or uploaded as supplementary information.

**Ethics approval**

Not applicable. This manuscript reports findings from a trial-registry review performed with no research performed on human participants or animal subjects as a result of this trial-registry review.



For peer review only - http://bmjopen.bmj.com/site/about/guidelines.xhtml

**Author Contributions**

NMN, SBL, TI, RK, MKBP, and MRS, wrote and approved the manuscript. NMN, SBL, RK, and MRS, designed the research. NMN performed the research search and extraction. NMN and MRS analysed the data.

For peer review only

## References

1. Collins R, Bowman L, Landray M, Peto R. The magic of randomization versus the myth of real-world evidence. N Engl J Med. 2020;382: 674–678. doi:10.1056/NEJMsb1901642

2. Bothwell LE, Greene JA, Podolsky SH, Jones DS, Malina D. Assessing the gold standard - Lessons from the history of RCTs. N Engl J Med. 2016;374: 2175–2181. doi:10.1056/NEJMms1604593

3. Zwierzyna M, Davies M, Hingorani AD, Hunter J. Clinical trial design and dissemination: Comprehensive analysis of clinicaltrials.gov and PubMed data since 2005. BMJ. 2018;361: k2130. doi:10.1136/bmj.k2130

4. DiMasi JA, Grabowski HG, Hansen RW. The Cost of Drug Development. N Engl J Med. 2015;372: 1972. doi:10.1056/nejmc1504317

5. Djulbegovic B, Kumar A, Soares HP, Hozo I, Bepler G, Clarke M, et al. Treatment success in cancer: New cancer treatment successes identified in phase 3 randomized controlled trials conducted by the National Cancer Institute-Sponsored Cooperative Oncology Groups, 1955 to 2006. Arch Intern Med. 2008;168: 632–642. doi:10.1001/archinte.168.6.632

6. Djulbegovic B, Kumar A, Glasziou P, Miladinovic B, Chalmers I. Medical research: Trial unpredictability yields predictable therapy gains. Nature. 2013;500: 395–396. doi:10.1038/500395a

7. Pocock SJ, Stone GW. The Primary Outcome Is Positive — Is That Good Enough? N Engl J Med. 2016;375: 971–979. doi:10.1056/nejmra1601511

8. Pocock SJ, Stone GW. The Primary Outcome Fails — What Next? N Engl J Med. 2016;375: 861–870. doi:10.1056/nejmra1510064

9. Nass SJ, Balogh E, Mendelsohn J. A national cancer clinical trials network:

For peer review only

Recommendations from the Institute of Medicine. Am J Ther. 2011;18: 382–391. doi:10.1097/MJT.0b013e3181ff7e23

10. Parmar MKB, Carpenter J, Sydes MR. More multiarm randomised trials of superiority are needed. Lancet. 2014;384: 283–284. doi:10.1016/S0140-6736(14)61122-3

11. Blagden SP, Billingham L, Brown LC, Buckland SW, Cooper AM, Ellis S, et al. Effective delivery of Complex Innovative Design (CID) cancer trials—A consensus statement. Br J Cancer. 2020;122: 473–482. doi:10.1038/s41416-019-0653-9

12. Royston P, Parmar MKB, Qian W. Novel designs for multi-arm clinical trials with survival outcomes with an application in ovarian cancer. Stat Med. 2003;22: 2239–2256. doi:10.1002/sim.1430

13. Parmar MKB, Barthel FMS, Sydes M, Langley R, Kaplan R, Eisenhauer E, et al. Speeding up the evaluation of new agents in cancer. J Natl Cancer Inst. 2008;100: 1204–1214. doi:10.1093/jnci/djn267

14. Bhatt DL, Mehta C. Adaptive Designs for Clinical Trials. N Engl J Med. 2016;375: 65–74. doi:10.1056/NEJMra1510061

15. Burnett T, Mozgunov P, Pallmann P, Villar SS, Wheeler GM, Jaki T. Adding flexibility to clinical trial designs: an example-based guide to the practical use of adaptive designs. BMC Med. 2020;18: 352. doi:10.1186/s12916-020-01808-2

16. Lin J, Bunn V. Comparison of multi-arm multi-stage design and adaptive randomization in platform clinical trials. Contemp Clin Trials. 2017;54: 48–59. doi:10.1016/j.cct.2017.01.003

17. Pallmann P, Bedding AW, Choodari-Oskooei B, Dimairo M, Flight L, Hampson



For peer review only - http://bmjopen.bmj.com/site/about/guidelines.xhtml

L V., et al. Adaptive designs in clinical trials: Why use them, and how to run and report them. BMC Med. 2018;16: 29. doi:10.1186/s12916-018-1017-7

18. Dimairo M, Julious SA, Todd S, Nicholl JP, Boote J. Cross-sector surveys assessing perceptions of key stakeholders towards barriers, concerns and facilitators to the appropriate use of adaptive designs in confirmatory trials. Trials. 2015;16. doi:10.1186/s13063-015-1119-x
19. Hatfield I, Allison A, Flight L, Julious SA, Dimairo M. Adaptive designs undertaken in clinical research: A review of registered clinical trials. Trials. 2016;17: 150. doi:10.1186/s13063-016-1273-9
20. Bewtra M, Lichtenstein GR. Mongersen and Smad-7 inhibition, not a Lucky 7 for patients with IBD: When trial design is as important as disease therapy. Am J Gastroenterol. 2020;115: 687–688. doi:10.14309/ajg.0000000000000564
21. Fogel DB. Factors associated with clinical trials that fail and opportunities for improving the likelihood of success: A review. Contemp Clin Trials Commun. 2018;11: 156–164.
22. Bothwell LE, Avorn J, Khan NF, Kesselheim AS. Adaptive design clinical trials: A review of the literature and ClinicalTrials.gov. BMJ Open. 2018;8: e018320. doi:10.1136/bmjopen-2017-018320
23. Collignon O, Koenig F, Koch A, Hemmings RJ, Pétavy F, Saint-Raymond A, et al. Adaptive designs in clinical trials: From scientific advice to marketing authorisation to the European Medicine Agency. Trials. 2018;19: 642. doi:10.1186/s13063-018-3012-x
24. Park JJH, Siden E, Zoratti MJ, Dron L, Harari O, Singer J, et al. Systematic review of basket trials, umbrella trials, and platform trials: A landscape analysis of master protocols. Trials. 2019;20: 572. doi:10.1186/s13063-019-3664-1



For peer review only - http://bmjopen.bmj.com/site/about/guidelines.xhtml

25. Sato A, Shimura M, Gosho M. Practical characteristics of adaptive design in phase 2 and 3 clinical trials. J Clin Pharm Ther. 2018;43: 170–180. doi:10.1111/jcpt.12617

26. Redman MW, Allegra CJ. The master protocol concept. Semin Oncol. 2016;42: 724–730. doi:10.1053/j.seminoncol.2015.07.009

27. Woodcock J, LaVange LM. Master protocols to study multiple therapies, multiple diseases, or both. N Engl J Med. 2017;377: 62–70. doi:10.1056/NEJMra1510062

28. Berry DA. The Brave New World of clinical cancer research: Adaptive biomarker-driven trials integrating clinical practice with clinical research. Mol Oncol. 2015;9: 951–959. doi:10.1016/j.molonc.2015.02.011

29. Bookman MA, Brady MF, McGuire WP, Harper PG, Alberts DS, Friedlander M, et al. Evaluation of new platinum-based treatment regimens in advanced-stage ovarian cancer: A phase III trial of the gynecologic cancer intergroup. J Clin Oncol. 2009;27: 1419–1425. doi:10.1200/JCO.2008.19.1684

30. Sydes MR, Parmar MKB, Mason MD, Clarke NW, Amos C, Anderson J, et al. Flexible trial design in practice - stopping arms for lack-of-benefit and adding research arms mid-trial in STAMPEDE: a multi-arm multi-stage randomized controlled trial. Trials. 2012;13: 168. doi:10.1186/1745-6215-13-168

31. Morrell L, Hordern J, Brown L, Sydes MR, Amos CL, Kaplan RS, et al. Mind the gap? the platform trial as a working environment. Trials. 2019;20: 297. doi:10.1186/s13063-019-3377-5

32. Noor NM, Pett SL, Esmail H, Crook AM, Vale CL, Sydes MR, et al. Adaptive platform trials using multi-arm, multi-stage protocols: getting fast answers in pandemic settings. F1000Research. 2020;9: 1109.

For peer review only

doi:10.12688/f1000research.26253.2

33. Collignon O, Burman CF, Posch M, Schiel A. Collaborative Platform Trials to Fight COVID-19: Methodological and Regulatory Considerations for a Better Societal Outcome. Clin Pharmacol Ther. 2021. doi:10.1002/cpt.2183

34. Dillman A, Zoratti MJ, Park JJH, Hsu G, Dron L, Smith G, et al. The landscape of emerging randomized clinical trial evidence for COVID-19 disease stages: A systematic review of global trial registries. Infect Drug Resist. 2020;13: 4577–4587. doi:10.2147/IDR.S288399

35. Bugin K, Woodcock J. Trends in COVID-19 therapeutic clinical trials. Nat Rev Drug Discov. 2021;20: 254–255. doi:10.1038/d41573-021-00037-3

36. Park JJ, Mogg R, Smith GE, Nakimuli-Mpungu E, Jehan F, Rayner CR, et al. How COVID-19 has fundamentally changed clinical research in global health. Lancet Glob Heal. 2021;9: e711-720.

37. Hague D, Townsend S, Masters L, Rauchenberger M, Van Looy N, Diaz-Montana C, et al. Changing platforms without stopping the train: Experiences of data management and data management systems when adapting platform protocols by adding and closing comparisons. Trials. 2019;20: 294. doi:10.1186/s13063-019-3322-7

38. Schiavone F, Bathia R, Letchemanan K, Masters L, Amos C, Bara A, et al. This is a platform alteration: A trial management perspective on the operational aspects of adaptive and platform and umbrella protocols. Trials. 2019;20: 264. doi:10.1186/s13063-019-3216-8

39. Lee KM, Brown LC, Jaki T, Stallard N, Wason J. Statistical consideration when adding new arms to ongoing clinical trials: the potentials and the caveats. Trials. 2021;22: 203. doi:10.1186/s13063-021-05150-7



For peer review only - http://bmjopen.bmj.com/site/about/guidelines.xhtml

40. Lee KM, Wason J, Stallard N. To add or not to add a new treatment arm to a multiarm study: A decision-theoretic framework. Stat Med. 2019;38: 3305–3321. doi:10.1002/sim.8194

41. Choodari-Oskooei B, Bratton DJ, Gannon MR, Meade AM, Sydes MR, Parmar MKB. Adding new experimental arms to randomised clinical trials: Impact on error rates. Clin Trials. 2020;17: 273–284. doi:10.1177/1740774520904346

42. Bennett M, Mander AP. Designs for adding a treatment arm to an ongoing clinical trial. Trials. 2020;21: 251. doi:10.1186/s13063-020-4073-1

43. Bratton DJ, Parmar MKB, Phillips PPJ, Choodari-Oskooei B. Type I error rates of multi-arm multi-stage clinical trials: Strong control and impact of intermediate outcomes. Trials. 2016;17: 309. doi:10.1186/s13063-016-1382-5

44. Proschan MA, Brittain EH. A primer on strong vs weak control of familywise error rate. Stat Med. 2020;39: 1407–1413. doi:10.1002/sim.8463

45. Parker RA, Weir CJ. Non-adjustment for multiple testing in multi-arm trials of distinct treatments: Rationale and justification. Clin Trials. 2020;17: 562–566. doi:10.1177/1740774520941419

46. Wason JMS, Robertson DS. Controlling type I error rates in multi-arm clinical trials: A case for the false discovery rate. Pharm Stat. 2021;20: 109–116. doi:10.1002/pst.2059

47. Collignon O, Gartner C, Haidich AB, James Hemmings R, Hofner B, Pétavy F, et al. Current Statistical Considerations and Regulatory Perspectives on the Planning of Confirmatory Basket, Umbrella, and Platform Trials. Clin Pharmacol Ther. 2020;107: 1059–1067. doi:10.1002/cpt.1804

48. Sydes MR, Egawa S, Sanders K, Amos C, Clarke N, Kimura T, et al. Reflections on attempted Anglo-Japanese collaboration on STAMPEDE: A

For peer review only

randomized controlled trial for men with prostate cancer. Int J Urol. 2011;18: 553–554. doi:10.1111/j.1442-2042.2011.02783.x

49. Parmar MKB, Sydes MR, Cafferty FH, Choodari-Oskooei B, Langley RE, Brown L, et al. Testing many treatments within a single protocol over 10 years at MRC Clinical Trials Unit at UCL: Multi-arm, multi-stage platform, umbrella and basket protocols. Clin Trials. 2017;14: 451–461. doi:10.1177/1740774517725697

50. Wason JMS, Brocklehurst P, Yap C. When to keep it simple - Adaptive designs are not always useful. BMC Med. 2019;17: 152. doi:10.1186/s12916-019-1391-9

51. Bretz F, Gallo P, Maurer W. Adaptive designs: The Swiss Army knife among clinical trial designs? Clin Trials. 2017;14: 417–424. doi:10.1177/1740774517699406

52. Fox RJ, Chataway J. Advancing trial design in progressive multiple sclerosis. Mult Scler. 2017;23: 1573–1578. doi:10.1177/1352458517729768

53. Zeissler ML, Li V, Parmar MKB, Carroll CB. Is It Possible to Conduct a Multi-Arm Multi-Stage Platform Trial in Parkinson’s Disease: Lessons Learned from Other Neurodegenerative Disorders and Cancer. J Parkinsons Dis. 2020;10: 413–428. doi:10.3233/JPD-191856

54. Moore CL, Stöhr W, Crook AM, Richert L, Leliévre JD, Pantaleo G, et al. Multi-arm, multi-stage randomised controlled trials for evaluating therapeutic HIV cure interventions. Lancet HIV. 2019;6: e334-340. doi:10.1016/S2352-3018(19)30082-7

For peer review only

# Figure Legends

**Figure 1 – Search strategy and inclusion of late-phase MAMS platform trials.**

This flow diagram represents the search criteria and results for late-phase MAMS platform trials identified and eligible for inclusion in this review. The date of lock for new trial registrations was 1 April 2021.

**Figure 2 – Increasing uptake of late-phase trials using the MAMS platform approach over time.**

Hosepipe plot demonstrating increasing uptake of MAMS platform for late-phase clinical trials over time, accelerated by uptake during the COVID-19 pandemic. The date of lock for new trial registrations was 1 April 2021.

**Figure 3 – Countries involved in late-phase APTs using the MAMS protocol approach.**

**(A)** Country where organisations based around the world which were leading late-phase MAMS platform trials in the pre-COVID-19 era and since the COVID-19 pandemic. **(B)** Countries around the world recruiting to late-phase MAMS platform trials in the pre-COVID-19 era and since the COVID-19 pandemic.

**Table 1 – Definition for late-phase adaptive platform trial using a MAMS protocol approach.**

Given the wide-ranging definitions and terms used in the clinical trials field, this was the definition used for eligibility and inclusion in this registry-based review.



For peer review only - http://bmjopen.bmj.com/site/about/guidelines.xhtml

**Table 2 – Characteristics of included trials using a MAMS protocol approach.**

Presentation of key characteristics regarding conduct of the trial including: review of disease areas, use of the MAMS term, type of late-phase trial, type of organisation leading the trial and the country of origin of the leading organisation.

**Table 3 – Characteristics of interventions and control groups for late-phase adaptive platform trial using a MAMS protocol approach.**

Presentation of key characteristics regarding comparison intervention arms and control arms in the trial including: type of intervention, repurposing of treatments, addition of new intervention arms, and type of control arm used in the MAMS platform.

For peer review only

# Supplementary information

**Figure S1. Cumulative uptake of MAMS platform protocol registrations for late-phase trials prior to and following the COVID-19 pandemic.**

**Figure S2. Anticipated maximum sample size at outset of late-phase MAMS platform trials.**

**Table S1. Search strategy and string used to identify late-phase MAMS platform trials.**

**Table S2. Characteristics by individual trial for the design, co-ordination and recruitment of late-phase MAMS platform protocols.**

**Table S3. Characteristics for trial sponsors and interventions assessed in late-phase MAMS platform trials.**

**Table S4. Characteristics for intervention arms, control group and anticipated initial maximum sample size across late-phase MAMS platform trials.**

**Table S5. Name, trial registry entry and trial registration number of late-phase MAMS platform trials.**



For peer review only

For peer review only - http://bmjopen.bmj.com/site/about/guidelines.xhtml

**Table 1 – Definition for late-phase adaptive platform trial using a MAMS protocol approach.**

| Late-phase, randomised-controlled trial using multi-arm, multi-stage (MAMS) protocol definition |
|---|
| • Late-phase defined as a phase three, seamless phase two/three, or a phase two trial which intended for phase three expansion at the outset even if this expansion does not ultimately occur.<br>• Randomisation of participants.<br>• Multi-arm defined as two or more actual or intended comparison interventions. There is typically the intention to add in new intervention arms into the platform, but this would not be considered mandatory and the addition of new intervention arms may not ultimately take place.<br>• Multi-stage defined as two or more actual or intended stages with an interim analysis in between stages, with the ability to stop recruitment to intervention arms following interim analysis. |



For peer review only - http://bmjopen.bmj.com/site/about/guidelines.xhtml

**Table 2 – Characteristics of included trials using a MAMS protocol approach.**

| Category | Number of MAMS trials | Percentage of MAMS trials |
|---|---|---|
| **Disease area** | | |
| Infection | 32 | 52% |
| Cancer | 18 | 29% |
| Neurology | 4 | 6% |
| Mental Health | 2 | 3% |
| Diabetes | 2 | 3% |
| Dermatology | 1 | 2% |
| Haematology | 1 | 2% |
| Inflammation | 1 | 2% |
| Surgery | 1 | 2% |
| **MAMS term used** | | |
| Yes | 31 | 50% |
| No | 31 | 50% |
| **Phase of trial** | | |
| Seamless phase 2/3 | 33 | 53% |
| Phase 3 | 29 | 47% |
| **Organisation leading trial** | | |
| Non-commercial | 55 | 89% |
| Commercial | 7 | 11% |
| **Country of organisation leading trial** | | |
| UK | 26 | 41% |
| USA | 20 | 32% |
| France | 5 | 8% |
| Switzerland | 2 | 3% |
| Australia | 1 | 2% |
| Austria | 1 | 2% |
| Brazil | 1 | 2% |
| Canada | 1 | 2% |
| Denmark | 1 | 2% |
| Netherlands | 1 | 2% |
| Pakistan | 1 | 2% |
| Singapore | 1 | 2% |
| Spain | 1 | 2% |

For peer review only



For peer review only - http://bmjopen.bmj.com/site/about/guidelines.xhtml

**Table 3 – Characteristics of interventions and control groups for late-phase adaptive platform trial using a MAMS protocol approach.**

| Category | Number of MAMS trials | Percentage of MAMS trials |
|---|---|---|
| **Type of interventions** | | |
| Medical treatments only | 58 | 93% |
| Surgical treatments only | 1 | 2% |
| Medical and surgical treatments | 2 | 3% |
| Psychiatric treatments only | 1 | 2% |
| **Repurposed medications** | | |
| None | 29 | 47% |
| All repurposed medications | 22 | 35% |
| Some repurposed medications | 6 | 10% |
| Not applicable | 5 | 8% |
| **Doses of intervention being compared** | | |
| No | 57 | 92% |
| Yes | 5 | 8% |
| **Durations of intervention(s) being compared** | | |
| No | 59 | 95% |
| Yes | 3 | 5% |
| **Plan to add in intervention arm(s)** | | |
| Yes | 56 | 90% |
| No | 6 | 10% |
| **Added in intervention arm(s)** | | |
| No | 55 | 89% |
| Yes | 7 | 11% |
| **Number of intervention comparison arms at outset** | | |
| 1 | 10 | 16% |
| 2 | 20 | 32% |
| 3 | 20 | 32% |
| 4 | 8 | 13% |
| 5 | 1 | 2% |
| 6 | 0 | 0% |
| 7 | 2 | 3% |
| 8 | 1 | 2% |
| **Number of intervention comparison arms assessed to date** | | |
| 1 | 8 | 13% |
| 2 | 17 | 28% |
| 3 | 21 | 34% |
| 4 | 10 | 16% |
| 5 | 1 | 2% |
| 6 | 0 | 0% |
| 7 | 1 | 2% |
| 8 | 1 | 2% |
| 9 | 0 | 0% |
| 10 | 2 | 3% |



For peer review only - http://bmjopen.bmj.com/site/about/guidelines.xhtml

| Category | Number of MAMS trials | Percentage of MAMS trials |
|---|---|---|
| **Control arm(s) in the platform** | | |
| No control group arm | 4 | 6% |
| 1 control arm - standard of care arm only | 38 | 61% |
| 1 control arm - placebo arm only | 17 | 27% |
| 2 control arms - Both placebo and standard of care arms | 3 | 5% |

For peer review only



For peer review only - http://bmjopen.bmj.com/site/about/guidelines.xhtml

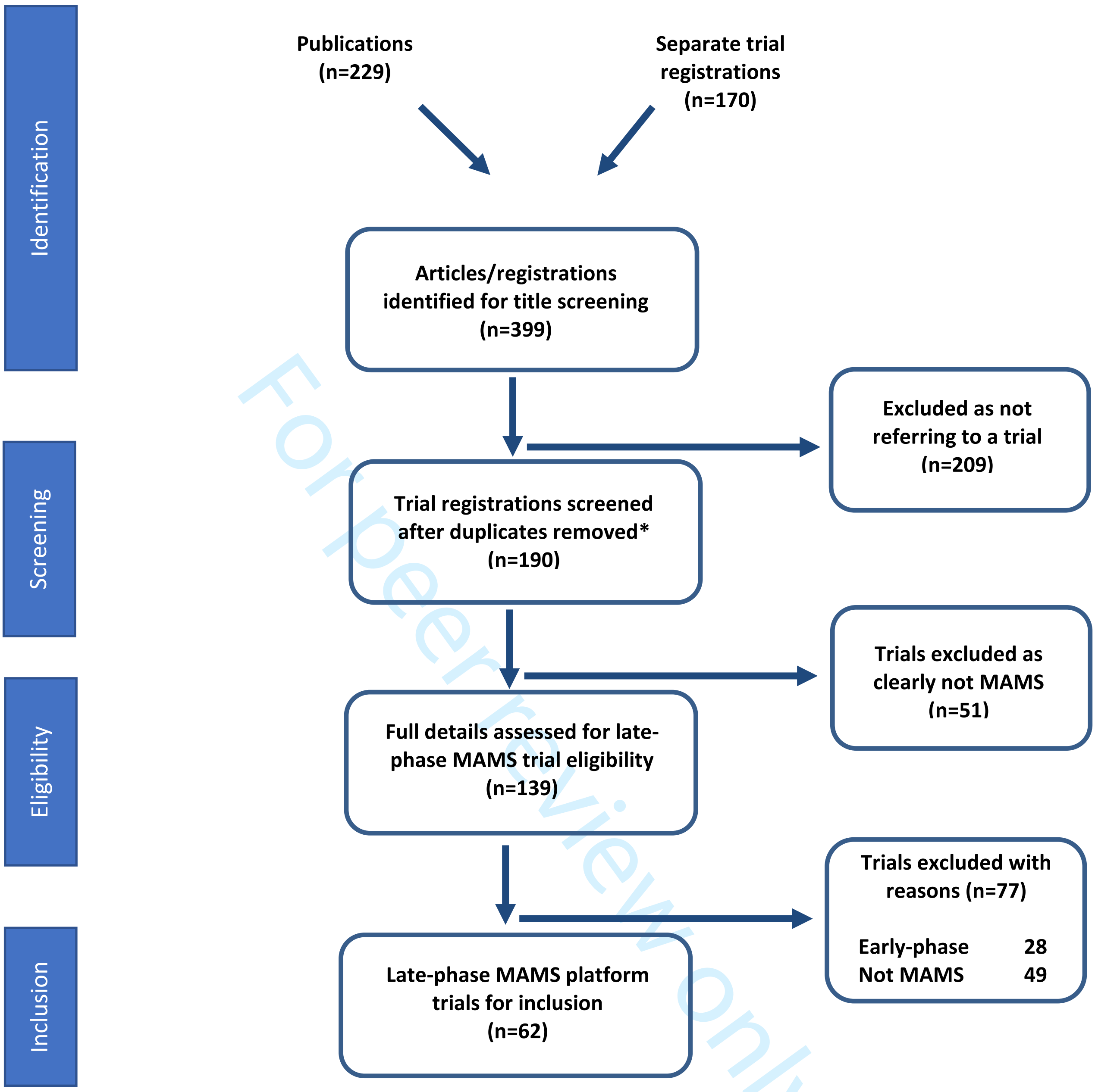


*all the identified MAMS publications provided a trial registration number in the manuscript. All the collated trial registrations then underwent screening.

For peer review only

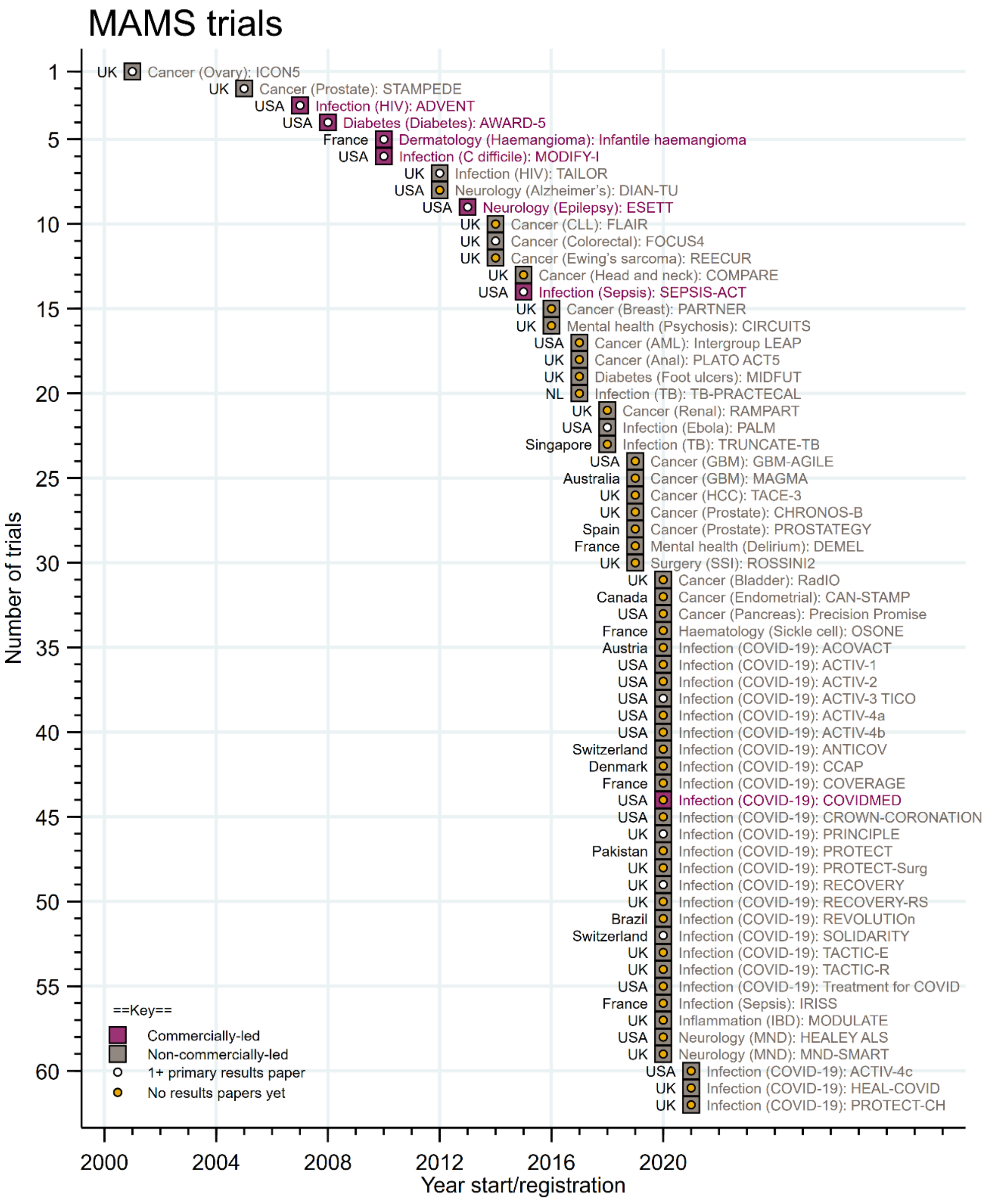
MAMS trials
Number of trials
Year start/registration
2000
2004
2008
2012
2016
2020
UK Cancer (Ovary): ICON5
UK Cancer (Prostate): STAMPEDE
USA Infection (HIV): ADVENT
USA Diabetes (Diabetes): AWARD-5
France Dermatology (Haemangioma): Infantile haemangioma
USA Infection (C difficile): MODIFY-I
UK Infection (HIV): TAILOR
USA Neurology (Alzheimer's): DIAN-TU
USA Neurology (Epilepsy): ESETT
UK Cancer (CLL): FLAIR
UK Cancer (Colorectal): FOCUS4
UK Cancer (Ewing's sarcoma): REECUR
UK Cancer (Head and neck): COMPARE
USA Infection (Sepsis): SEPSIS-ACT
UK Cancer (Breast): PARTNER
UK Mental health (Psychosis): CIRCUITS
USA Cancer (AML): Intergroup LEAP
UK Cancer (Anal): PLATO ACT5
UK Diabetes (Foot ulcers): MIDFUT
NL Infection (TB): TB-PRACTECAL
UK Cancer (Renal): RAMPART
USA Infection (Ebola): PALM
Singapore Infection (TB): TRUNCATE-TB
USA Cancer (GBM): GBM-AGILE
Australia Cancer (GBM): MAGMA
UK Cancer (HCC): TACE-3
UK Cancer (Prostate): CHRONOS-B
Spain Cancer (Prostate): PROSTATEGY
France Mental health (Delirium): DEMEL
UK Surgery (SSI): ROSSINI2
UK Cancer (Bladder): RadIO
Canada Cancer (Endometrial): CAN-STAMP
USA Cancer (Pancreas): Precision Promise
France Haematology (Sickle cell): OSONE
Austria Infection (COVID-19): ACOVACT
USA Infection (COVID-19): ACTIV-1
USA Infection (COVID-19): ACTIV-2
USA Infection (COVID-19): ACTIV-3 TICO
USA Infection (COVID-19): ACTIV-4a
USA Infection (COVID-19): ACTIV-4b
Switzerland Infection (COVID-19): ANTICOV
Denmark Infection (COVID-19): CCAP
France Infection (COVID-19): COVERAGE
USA Infection (COVID-19): COVIDMED
USA Infection (COVID-19): CROWN-CORONATION
UK Infection (COVID-19): PRINCIPLE
Pakistan Infection (COVID-19): PROTECT
UK Infection (COVID-19): PROTECT-Surg
UK Infection (COVID-19): RECOVERY
UK Infection (COVID-19): RECOVERY-RS
Brazil Infection (COVID-19): REVOLUTIOn
Switzerland Infection (COVID-19): SOLIDARITY
UK Infection (COVID-19): TACTIC-E
UK Infection (COVID-19): TACTIC-R
USA Infection (COVID-19): Treatment for COVID
France Infection (Sepsis): IRISS
UK Inflammation (IBD): MODULATE
USA Neurology (MND): HEALEY ALS
UK Neurology (MND): MND-SMART
USA Infection (COVID-19): ACTIV-4c
UK Infection (COVID-19): HEAL-COVID
UK Infection (COVID-19): PROTECT-CH
==Key==
Commercially-led
Non-commercially-led
1+ primary results paper
No results papers yet

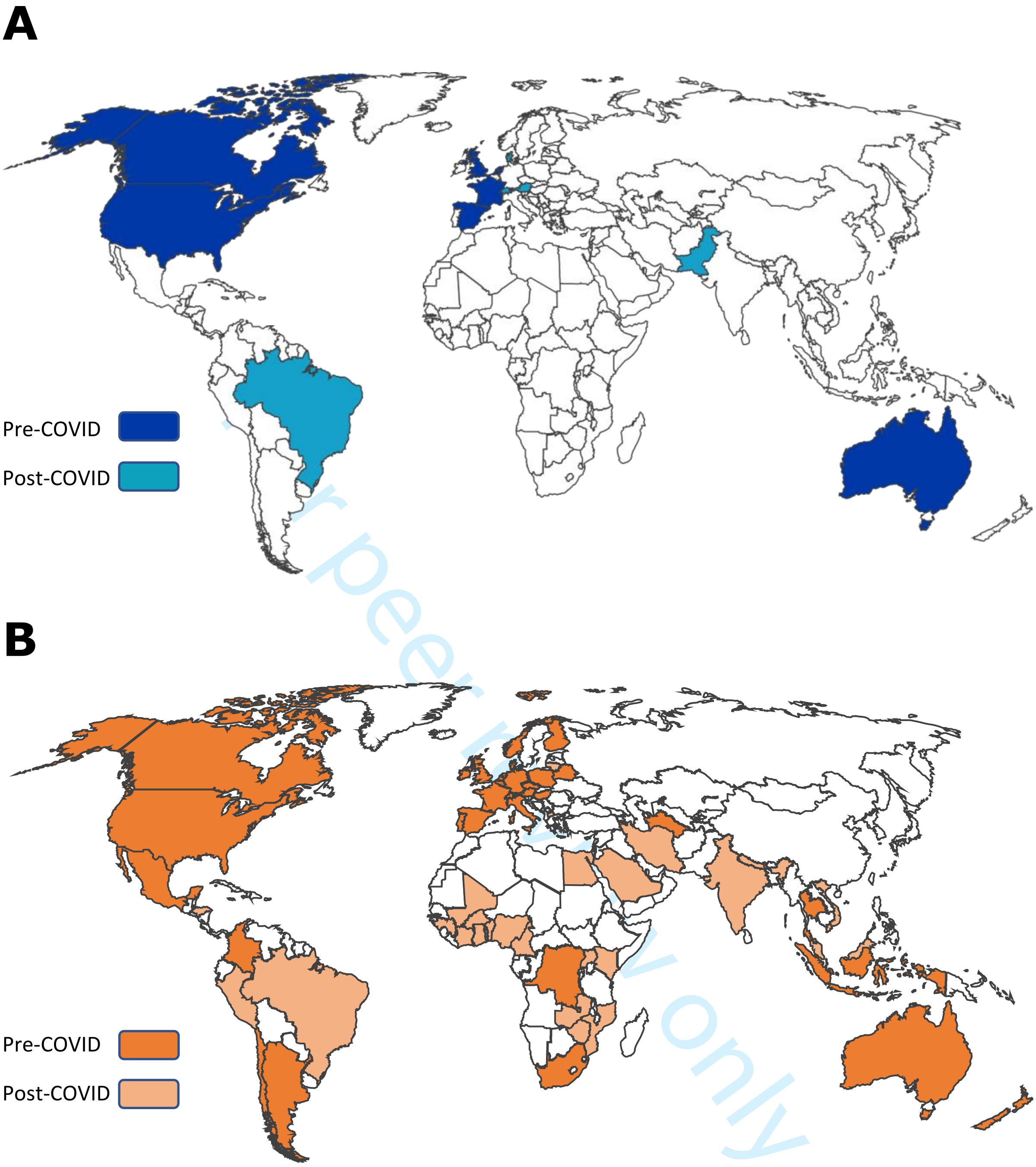
A
Pre-COVID
Post-COVID
B
Pre-COVID
Post-COVID

**Figure S1. Cumulative uptake of MAMS platform protocol registrations for late-phase trials prior to and following the COVID-19 pandemic.**

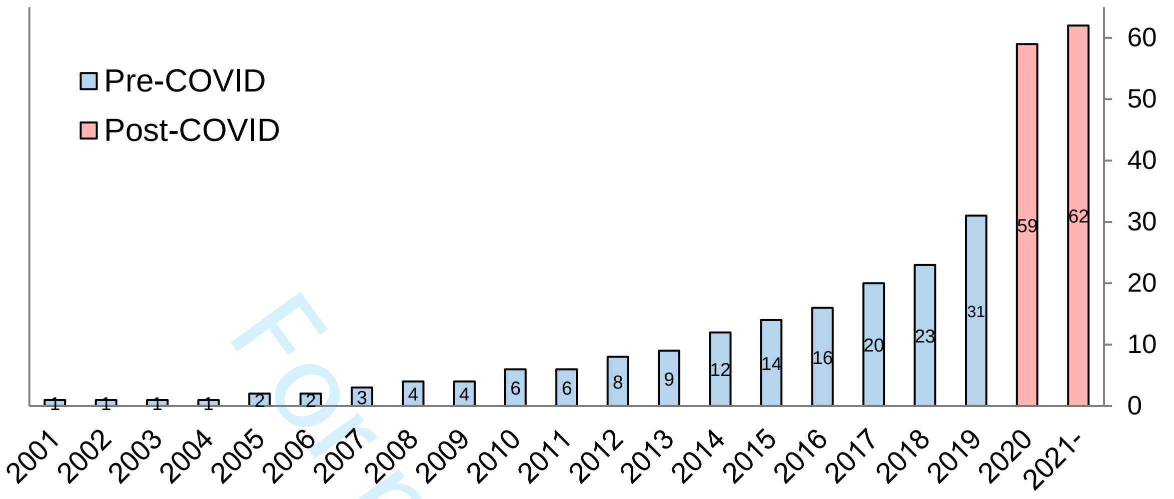

**Figure S2. Anticipated maximum sample size at outset of late-phase MAMS platform trials.**

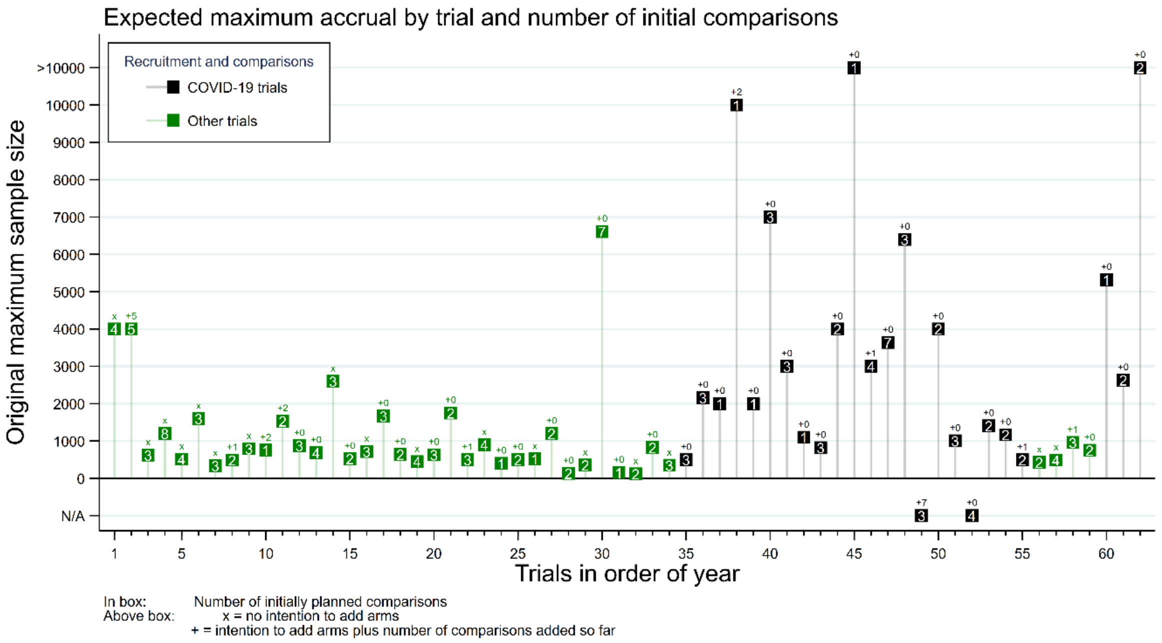


review only

**Table S1. Search strategy and string used to identify late-phase MAMS platform trials.**

| Search strategy and string used to identify late-phase MAMS platform trials. |
|---|
| We examined the uptake of registered, late-phase MAMS platform trials by conducting searches both in individual registries including ClinicalTrials.gov, the EU clinical trials register, which contains all trials registered on the European Union Drug Regulating Authorities Clinical Trials Database (EudraCT), the Australian New Zealand Clinical Trials Registry (ANZCTR), International Standard Randomised Controlled Trial Number (ISRCTN) registry, the Pan African Clinical Trials Registry (PACTR), and the overall World Health Organisation (WHO) International Clinical Trial Registry Platform (ICTRP), which is a summary of worldwide trial databases. We also searched the databases PubMed, Medline, Cochrane Library, Global Health Library, and EMBASE using a structured search strategy for published trials. The final search strategy was completed on 1 April 2021. For published articles, both the titles and abstracts were searched. Publications and/or trial registry results could include abstracts/trial registrations in a language other than English, if supplied by the publisher/entrant, but defaulted to the English abstract where an entry existed. No filters or limits were applied.<br><br>The following search terms used were: “adaptive trial” OR “adaptive clinical trial” OR “platform trial” OR “platform clinical trial” OR “adaptive platform trial” OR “adaptive platform clinical trial”. “multi-arm multi-stage” OR “MAMS”. “master protocol” OR “master trial protocol” OR “trial master protocol”. “late-phase” OR “phase 3” OR “phase 2-3” OR “phase 2/3” OR “phase 2 to 3” OR “seamless phase 2/3” OR “seamless phase 2-3”. “seamless” OR “seamless design” OR “adaptive seamless” OR “adaptive seamless design”, singly or in combination.<br><br>All late-phase MAMS platform trials included in this review were registered on a trial registry by 1 April 2021, which was the final day of identification and data collection. This was the date used to lock the data for this manuscript, including both registration of a trial and information about ongoing trial progress. Single extraction of data took place with subsequent review and verification of findings |

following discussion with collaborators and colleagues to ensure no missing or inappropriately included trials were in the final list. Information about trials was obtained from data linked to trial registry numbers, including data from registry entries, publications (if present and linked to trial registry number), trial websites if link was provided on trial registry entry, and review of publicly available documents such as trial protocols and statistical analysis plans if they were linked to the trial registry entry.

For peer review only

**Table S2. Characteristics by individual trial for the design, co-ordination and recruitment of late-phase MAMS platform protocols.**

| Name | MAMS term used | Phase of trial | Organisation leading | Country of leading organisation | Number of recruiting countries |
|---|---|---|---|---|---|
| **2001** | | | | | |
| ICON5 | Yes | 3 | Non-commercial | UK | 1 |
| **2005** | | | | | |
| STAMPEDE | Yes | Seamless 2/3 | Non-commercial | UK | 2 |
| **2007** | | | | | |
| ADVENT | No | 3 | Commercial | USA | 1 |
| **2008** | | | | | |
| AWARD-5 | No | Seamless 2/3 | Commercial | USA | 1 |
| **2010** | | | | | |
| Infantile haemangioma | No | Seamless 2/3 | Commercial | France | 1 |
| MODIFY-I | No | 3 | Commercial | USA | 19 |
| **2012** | | | | | |
| DIAN-TU | No | Seamless 2/3 | Non-commercial | USA | 9 |
| TAILOR | Yes | Seamless 2/3 option | Non-commercial | UK | 1 |
| **2013** | | | | | |
| ESETT | No | 3 | Commercial | USA | 1 |
| **2014** | | | | | |
| FOCUS4 | Yes | Seamless 2/3 option | Non-commercial | UK | 1 |
| REECUR | Yes | Seamless 2/3 | Non-commercial | UK | 16 |
| FLAIR | No | 3 | Non-commercial | UK | 1 |
| **2015** | | | | | |
| COMPARE | Yes | 3 | Non-commercial | UK | 1 |
| SEPSIS-ACT | No | Seamless 2/3 option | Commercial | USA | 5 |
| **2016** | | | | | |
| CIRCUITS | Yes | 3 | Non-commercial | UK | 1 |
| PARTNER | No | Seamless 2/3 | Non-commercial | UK | 1 |
| **2017** | | | | | |
| Intergroup LEAP | No | Seamless 2/3 | Non-commercial | USA | 1 |
| MIDFUT | Yes | Seamless 2/3 | Non-commercial | UK | 1 |
| PLATO ACT5 | Yes | Seamless 2/3 | Non-commercial | UK | 1 |
| TB-PRACTECAL | Yes | Seamless 2/3 | Non-commercial | Netherlands | 3 |
| PALM | No | Seamless 2/3 | Non-commercial | USA | 1 |
| RAMPART | Yes | 3 | Non-commercial | UK | 5 |
| TRUNCATE-TB | Yes | Seamless 2/3 | Non-commercial | Singapore | 4 |
| **2018** | | | | | |
| CHRONOS-B | Yes | Seamless 2/3 | Non-commercial | UK | 1 |
| DEMEL | Yes | Seamless 2/3 | Non-commercial | France | 1 |
| GBM-AGILE | No | Seamless 2/3 | Non-commercial | USA | 2 |
| MAGMA | Yes | 3 | Non-commercial | Australia | 1 |
| PROSTATEGY | Yes | Seamless 2/3 | Non-commercial | Spain | 1 |
| ROSSINI2 | Yes | 3 | Non-commercial | UK | 1 |
| TACE-3 | Yes | Seamless 2/3 | Non-commercial | UK | 2 |
| **2020** | | | | | |
| CAN-STAMP | Yes | Seamless 2/3 | Non-commercial | Canada | 1 |
| HEALEY ALS | No | Seamless 2/3 | Non-commercial | USA | 1 |
| IRISS | Yes | 3 | Non-commercial | France | 1 |

For peer review only

| Name | MAMS term used | Phase of trial | Organisation leading | Country of leading organisation | Number of recruiting countries |
|---|---|---|---|---|---|
| MND-SMART | Yes | Seamless 2/3 | Non-commercial | UK | 1 |
| MODULATE | Yes | Seamless 2/3 | Non-commercial | UK | 1 |
| OSONE | Yes | 3 | Non-commercial | France | 1 |
| Precision Promise | No | Seamless 2/3 | Non-commercial | USA | 1 |
| RadIO | No | Seamless 2/3 | Non-commercial | UK | 1 |
| ACOVACT | No | Seamless 2/3 | Non-commercial | Austria | 1 |
| ACTIV-1 | No | 3 | Non-commercial | USA | 1 |
| ACTIV-2 | No | Seamless 2/3 | Non-commercial | USA | 1 |
| ACTIV-3 TICO | Yes | 3 | Non-commercial | USA | 5 |
| ACTIV-4a | No | 3 | Non-commercial | USA | 2 |
| ACTIV-4b | No | 3 | Non-commercial | USA | 1 |
| ANTICOV | No | 3 | Non-commercial | Switzerland | 13 |
| CCAP | No | 3 | Non-commercial | Denmark | 1 |
| COVERAGE | Yes | Seamless 2/3 | Non-commercial | France | 1 |
| COVIDMED | No | Seamless 2/3 | Commercial | USA | 1 |
| CROWN-CORONATION | No | 3 | Non-commercial | USA | 6 |
| PRINCIPLE | No | 3 | Non-commercial | UK | 1 |
| PROTECT | Yes | 3 | Non-commercial | Pakistan | 1 |
| PROTECT-Surg | Yes | 3 | Non-commercial | UK | 9 |
| RECOVERY | Yes | 3 | Non-commercial | UK | 3 |
| RECOVERY-RS | Yes | 3 | Non-commercial | UK | 1 |
| REVOLUTIOn | Yes | Seamless 2/3 | Non-commercial | Brazil | 1 |
| SOLIDARITY | No | 3 | Non-commercial | Switzerland | 30 |
| TACTIC-E | No | Seamless 2/3 | Non-commercial | UK | 1 |
| TACTIC-R | No | 3 | Non-commercial | UK | 1 |
| Treatment for COVID | No | Seamless 2/3 | Non-commercial | USA | 1 |
| **2021** | | | | | |
| ACTIV-4c | No | 3 | Non-commercial | USA | 1 |
| HEAL-COVID | No | 3 | Non-commercial | UK | 1 |
| PROTECT-CH | Yes | 3 | Non-commercial | UK | 1 |

For peer review only

**Table S3. Characteristics for trial sponsors and interventions assessed in late-phase MAMS platform trials.**

| Name | Type of interventions | Sponsor | Repurposed intervention(s) | Doses of intervention | Durations of intervention |
|---|---|---|---|---|---|
| **2001** | | | | | |
| ICON5 | Medical | Non-commercial | None | No | No |
| **2005** | | | | | |
| STAMPEDE | Medical | Non-commercial | Some | No | No |
| **2007** | | | | | |
| ADVENT | Medical | Commercial | None | Yes | No |
| **2008** | | | | | |
| AWARD-5 | Medical | Commercial | None | Yes | No |
| **2010** | | | | | |
| Infantile haemangioma | Medical | Commercial | All | Yes | No |
| MODIFY-I | Medical | Commercial | None | No | No |
| **2012** | | | | | |
| DIAN-TU | Medical | Non-commercial | None | Yes | No |
| TAILOR | Medical | Non-commercial | All | Yes | No |
| **2013** | | | | | |
| ESETT | Medical | Commercial | All | No | No |
| **2014** | | | | | |
| FOCUS4 | Medical | Non-commercial | Some | No | No |
| REECUR | Medical | Non-commercial | None | No | No |
| FLAIR | Medical | Non-commercial | None | No | No |
| **2015** | | | | | |
| COMPARE | Medical and surgical | Non-commercial | None | No | No |
| SEPSIS-ACT | Medical | Commercial | None | Yes | No |
| **2016** | | | | | |
| CIRCUITS | Psychiatric | Non-commercial | N/A | Yes | No |
| PARTNER | Medical | Non-commercial | None | No | Yes |
| **2017** | | | | | |
| Intergroup LEAP | Medical | Non-commercial | None | No | No |
| MIDFUT | Medical and surgery | Non-commercial | None | No | No |
| PLATO ACT5 | Medical | Non-commercial | N/A | Yes | No |
| TB-PRACTECAL | Medical | Non-commercial | None | No | Yes |
| **2018** | | | | | |
| PALM | Medical | Non-commercial | None | No | No |
| RAMPART | Medical | Non-commercial | None | No | No |
| TRUNCATE-TB | Medical | Non-commercial | None | No | No |
| **2019** | | | | | |
| CHRONOS-B | Medical | Non-commercial | None | No | No |
| DEMEL | Medical | Non-commercial | Yes | Yes | No |
| GBM-AGILE | Medical | Non-commercial | None | No | No |
| MAGMA | Medical | Non-commercial | None | No | Yes |
| PROSTATEGY | Medical | Non-commercial | None | No | No |
| ROSSINI2 | Surgery | Non-commercial | N/A | No | No |
| TACE-3 | Medical | Non-commercial | None | No | No |
| **2020** | | | | | |
| CAN-STAMP | Medical | Non-commercial | None | No | No |

| Name | Type of interventions | Sponsor | Repurposed intervention(s) | Doses of intervention | Durations of intervention |
|---|---|---|---|---|---|
| HEALEY ALS | Medical | Non-commercial | None | No | No |
| IRISS | Medical | Non-commercial | All | Yes | No |
| MND-SMART | Medical | Non-commercial | Some | No | No |
| MODULATE | Medical | Non-commercial | All | No | No |
| OSONE | Medical | Non-commercial | N/A | Yes | No |
| Precision Promise | Medical | Non-commercial | None | No | No |
| RadIO | Medical | Non-commercial | None | No | No |
| ACOVACT | Medical | Non-commercial | Some | No | No |
| ACTIV-1 | Medical | Non-commercial | All | No | No |
| ACTIV-2 | Medical | Non-commercial | None | No | No |
| ACTIV-3 TICO | Medical | Non-commercial | None | No | No |
| ACTIV-4a | Medical | Non-commercial | All | Yes | No |
| ACTIV-4b | Medical | Non-commercial | All | No | No |
| ANTICOV | Medical | Non-commercial | All | No | No |
| CCAP | Medical | Non-commercial | None | No | No |
| COVERAGE | Medical | Non-commercial | All | No | No |
| COVIDMED | Medical | Commercial | All | No | No |
| CROWN-CORONATION | Medical | Non-commercial | All | No | No |
| PRINCIPLE | Medical | Non-commercial | All | No | No |
| PROTECT | Medical | Non-commercial | All | No | No |
| PROTECT-Surg | Medical | Non-commercial | All | No | No |
| RECOVERY | Medical | Non-commercial | Some | No | No |
| RECOVERY-RS | Medical | Non-commercial | N/A | No | No |
| REVOLUTIOn | Medical | Non-commercial | All | No | No |
| SOLIDARITY | Medical | Non-commercial | Some | No | No |
| TACTIC-E | Medical | Non-commercial | None | No | No |
| TACTIC-R | Medical | Non-commercial | All | No | No |
| Treatment for COVID | Medical | Non-commercial | All | No | No |
| **2021** | | | | | |
| ACTIV-4c | Medical | Non-commercial | All | No | No |
| HEAL-COVID | Medical | Non-commercial | All | No | No |
| PROTECT-CH | Medical | Non-commercial | Yes | No | No |

For peer review only

**Table S4. Characteristics for intervention arms, control group and anticipated initial maximum sample size across late-phase MAMS platform trials.**

| Name | Number of arms at outset | Number of arms assessed in total | Plan to add arms | Actually added arms | Control group | Planned maximum sample size |
|---|---|---|---|---|---|---|
| **2001** | | | | | | |
| ICON5 | 4 | 4 | No | No | SOC | 4000 |
| **2005** | | | | | | |
| STAMPEDE | 5 | 10 | Yes | Yes | SOC | 4000 |
| **2007** | | | | | | |
| ADVENT | 3 | 3 | No | No | Placebo | 624 |
| **2008** | | | | | | |
| AWARD-5 | 8 | 8 | No | No | Placebo and SOC | 1202 |
| **2010** | | | | | | |
| Infantile haemangioma | 4 | 4 | No | No | Placebo | 512 |
| MODIFY-I | 3 | 3 | No | No | Placebo and SOC | 1600 |
| **2012** | | | | | | |
| DIAN-TU | 2 | 3 | Yes | Yes | Placebo | 490 |
| TAILOR | 3 | 3 | No | No | SOC | 336 |
| **2013** | | | | | | |
| ESETT | 3 | 3 | No | No | None | 795 |
| **2014** | | | | | | |
| FOCUS4 | 2 | 4 | Yes | Yes | Placebo | 1536 |
| REECUR | 3 | 3 | es | No | SOC | 875 |
| FLAIR | 1 | 3 | Yes | Yes | SOC | 754 |
| **2015** | | | | | | |
| COMPARE | 4 | 4 | Yes | No | SOC | 685 |
| SEPSIS-ACT | 3 | 3 | No | No | Placebo | 2600 |
| **2016** | | | | | | |
| CIRCUITS | 3 | 3 | No | No | SOC | 720 |
| PARTNER | 2 | 2 | Yes | No | SOC | 527 |
| **2017** | | | | | | |
| Intergroup LEAP | 3 | 3 | Yes | No | SOC | 1670 |
| MIDFUT | 4 | 4 | No | No | SOC | 447 |
| PLATO ACT5 | 2 | 2 | Yes | No | SOC | 640 |
| TB-PRACTECAL | 3 | 3 | Yes | No | SOC | 630 |
| PALM | 3 | 4 | Yes | Yes | None | 500 |
| RAMPART | 2 | 2 | Yes | No | SOC | 1750 |
| TRUNCATE-TB | 4 | 4 | No | No | SOC | 900 |
| **2018** | | | | | | |
| CHRONOS-B | 2 | 2 | Yes | No | SOC | 1200 |
| DEMEL | 2 | 2 | No | No | Placebo | 355 |
| GBM-AGILE | 1 | 1 | Yes | No | SOC | 400 |
| MAGMA | 2 | 2 | Yes | No | SOC | 500 |
| PROSTATEGY | 2 | 2 | Yes | No | SOC | 135 |
| ROSSINI2 | 7 | 7 | Yes | No | SOC | 6610 |
| TACE-3 | 1 | 1 | No | No | SOC | 522 |
| **2020** | | | | | | |
| CAN-STAMP | 2 | 2 | No | No | None | 120 |
| HEALEY ALS | 3 | 4 | Yes | Yes | Placebo | 960 |
| IRISS | 2 | 2 | No | No | Placebo | 429 |

For peer review only - http://bmjopen.bmj.com/site/about/guidelines.xhtml

| Name | Number of arms at outset | Number of arms assessed in total | Plan to add arms | Actually added arms | Control group | Planned maximum sample size |
|---|---|---|---|---|---|---|
| MND-SMART | 2 | 2 | Yes | No | Placebo | 750 |
| MODULATE | 4 | 4 | No | No | SOC | 491 |
| OSONE | 3 | 3 | No | No | SOC | 350 |
| Precision Promise | 2 | 2 | Yes | No | SOC | 825 |
| RadIO | 1 | 1 | Yes | No | SOC | 159 |
| ACOVACT | 3 | 3 | Yes | No | SOC | 500 |
| ACTIV-1 | 3 | 3 | Yes | No | SOC | 2160 |
| ACTIV-2 | 1 | 1 | Yes | No | SOC | 2000 |
| ACTIV-3 TICO | 1 | 3 | Yes | Yes | Placebo and SOC | 10000 |
| ACTIV-4a | 1 | 1 | Yes | No | SOC | 2000 |
| ACTIV-4b | 3 | 3 | Yes | No | Placebo | 7000 |
| ANTICOV | 3 | 3 | Yes | No | SOC | 3000 |
| CCAP | 1 | 1 | Yes | No | Placebo | 1100 |
| COVERAGE | 3 | 3 | Yes | No | Placebo | 820 |
| COVIDMED | 2 | 2 | Yes | No | Placebo | 4000 |
| CROWN-CORONATION | 1 | 1 | Yes | Yes | Placebo | 30000 |
| PRINCIPLE | 4 | 6 | Yes | Yes | SOC | 3000 |
| PROTECT | 7 | 7 | Yes | No | None | 3640 |
| PROTECT-Surg | 3 | 3 | Yes | No | SOC | 6400 |
| RECOVERY | 3 | 10 | Yes | Yes | SOC | N/A |
| RECOVERY-RS | 2 | 2 | Yes | No | SOC | 4000 |
| REVOLUTIOn | 3 | 3 | Yes | No | Placebo | 1005 |
| SOLIDARITY | 4 | 4 | Yes | Yes | SOC | N/A |
| TACTIC-E | 2 | 2 | Yes | No | SOC | 1407 |
| TACTIC-R | 2 | 2 | Yes | No | SOC | 1167 |
| Treatment for COVID | 2 | 3 | Yes | No | Placebo | 495 |
| **2021** | | | | | | |
| ACTIV-4c | 1 | 1 | Yes | Yes | Placebo | 5320 |
| HEAL-COVID | 2 | 2 | Yes | Yes | SOC | 2631 |
| PROTECT-CH | 2 | 2 | Yes | Yes | SOC | 12000 |

For peer review only

**Table S5. Name, trial registry entry and trial registration number of late-phase MAMS platform trials.**

| Name | Trial registration number | Trial registry |
|---|---|---|
| ICON5 | NCT00011986 | Clinicaltrials.gov |
| STAMPEDE | NCT00268476 | Clinicaltrials.gov |
| ADVENT | NCT00547898 and NCT01374490 | Clinicaltrials.gov |
| AWARD-5 | NCT00734474 | Clinicaltrials.gov |
| Infantile haemangioma | NCT01056341 | Clinicaltrials.gov |
| MODIFY-I | NCT01241552 | Clinicaltrials.gov |
| DIAN-TU | NCT01760005 | Clinicaltrials.gov |
| TAILOR | ISRCTN51069819 | ISRCTN registry |
| ESETT | NCT01960075 | Clinicaltrials.gov |
| FOCUS4 | ISRCTN90061546 | ISRCTN registry |
| REECUR | ISRCTN36453794 | ISRCTN registry |
| FLAIR | ISRCTN01844152 | ISRCTN registry |
| COMPARE | NCT04116047 | Clinicaltrials.gov |
| SEPSIS-ACT | NCT02508649 | Clinicaltrials.gov |
| CIRCUITS | ISRCTN14678860 | ISRCTN registry |
| PARTNER | NCT03150576 | Clinicaltrials.gov |
| Intergroup LEAP | NCT03092674 | Clinicaltrials.gov |
| MIDFUT | ISRCTN64926597 | ISRCTN registry |
| PLATO ACT5 | ISRCTN88455282 | ISRCTN registry |
| TB-PRACTECAL | NCT02589782 | Clinicaltrials.gov |
| PALM | NCT03719586 | Clinicaltrials.gov |
| RAMPART | NCT03288532 | Clinicaltrials.gov |
| TRUNCATE-TB | NCT03474198 | Clinicaltrials.gov |
| CHRONOS-B | NCT04049747 | Clinicaltrials.gov |
| DEMEL | NCT03524937 | Clinicaltrials.gov |
| GBM-AGILE | NCT03970447 | Clinicaltrials.gov |
| MAGMA | ACTRN12618001944224 | Australian New Zealand Clinical Trials Registry |
| PROSTATEGY | NCT03879122 | Clinicaltrials.gov |
| ROSSINI2 | NCT03838575 | Clinicaltrials.gov |
| TACE-3 | ISRCTN12053408 | ISRCTN registry |
| CAN-STAMP | NCT04159155 | Clinicaltrials.gov |
| HEALEY ALS | NCT04297683 | Clinicaltrials.gov |
| IRISS | NCT04031573 | Clinicaltrials.gov |
| MND-SMART | NCT04302870 | Clinicaltrials.gov |
| MODULATE | ISRCTN16086699 | ISRCTN registry |
| OSONE | NCT03976180 | Clinicaltrials.gov |
| Precision Promise | NCT04229004 | Clinicaltrials.gov |
| RadIO | ISRCTN43698103 | ISRCTN registry |
| ACOVACT | NCT04351724 | Clinicaltrials.gov |
| ACTIV-1 | NCT04593940 | Clinicaltrials.gov |
| ACTIV-2 | NCT04518410 | Clinicaltrials.gov |
| ACTIV-3 TICO | NCT04501978 | Clinicaltrials.gov |
| ACTIV-4a | NCT04505774 | Clinicaltrials.gov |
| ACTIV-4b | NCT04498273 | Clinicaltrials.gov |
| ANTICOV | PACTR202006537901307 | Pan African Clinical Trials Registry |
| CCAP | NCT04345289 | Clinicaltrials.gov |
| COVERAGE | NCT04356495 | Clinicaltrials.gov |
| COVIDMED | NCT04328012 | Clinicaltrials.gov |
| CROWN-CORONATION | NCT04333732 | Clinicaltrials.gov |

For peer review only - http://bmjopen.bmj.com/site/about/guidelines.xhtml

| Name | Trial registration number | Trial registry |
|---|---|---|
| PRINCIPLE | ISRCTN86534580 | ISRCTN registry |
| PROTECT | NCT04338698 | Clinicaltrials.gov |
| PROTECT-Surg | NCT04386070 | Clinicaltrials.gov |
| RECOVERY | ISRCTN50189673 | ISRCTN registry |
| RECOVERY-RS | ISRCTN126912075 | ISRCTN registry |
| REVOLUTIOn | NCT04468087 | Clinicaltrials.gov |
| SOLIDARITY | ISRCTN83971151 | ISRCTN registry |
| TACTIC-E | NCT04393246 | Clinicaltrials.gov |
| TACTIC-R | NCT04390464 | Clinicaltrials.gov |
| Treatment for COVID | NCT04354428 | Clinicaltrials.gov |
| ACTIV-4c | NCT04650087 | Clinicaltrials.gov |
| PROTECT-CH | EudraCT2021-000185-15 | EU clinical trials register |
| HEAL-COVID | NCT04801940 | Clinicaltrials.gov |

For peer review only

# PRISMA 2020 Checklist

| Section and Topic | Item # | Checklist item | Location where item is reported |
|---|---|---|---|
| **TITLE** | | | |
| Title | 1 | Identify the report as a systematic review. | 1, trial-registry review and not a formal systematic review. |
| **ABSTRACT** | | | |
| Abstract | 2 | See the PRISMA 2020 for Abstracts checklist. | 2 |
| **INTRODUCTION** | | | |
| Rationale | 3 | Describe the rationale for the review in the context of existing knowledge. | 2,5-7 |
| Objectives | 4 | Provide an explicit statement of the objective(s) or question(s) the review addresses. | 7 |
| **METHODS** | | | |
| Eligibility criteria | 5 | Specify the inclusion and exclusion criteria for the review and how studies were grouped for the syntheses. | 8-9 |
| Information sources | 6 | Specify all databases, registers, websites, organisations, reference lists and other sources searched or consulted to identify studies. Specify the date when each source was last searched or consulted. | 8 |
| Search strategy | 7 | Present the full search strategies for all databases, registers and websites, including any filters and limits used. | 8 |
| Selection process | 8 | Specify the methods used to decide whether a study met the inclusion criteria of the review, including how many reviewers screened each record and each report retrieved, whether they worked independently, and if applicable, details of automation tools used in the process. | 8-9 |
| Data collection process | 9 | Specify the methods used to collect data from reports, including how many reviewers collected data from each report, whether they worked independently, any processes for obtaining or confirming data from study investigators, and if applicable, details of automation tools used in the process. | 8-9 |
| Data items | 10a | List and define all outcomes for which data were sought. Specify whether all results that were compatible with each outcome domain in each study were sought (e.g. for all measures, time points, analyses), and if not, the methods used to decide which results to collect. | 9 |
| | 10b | List and define all other variables for which data were sought (e.g. participant and intervention characteristics, funding sources). Describe any assumptions made about any missing or unclear information. | 9 |
| Study risk of bias assessment | 11 | Specify the methods used to assess risk of bias in the included studies, including details of the tool(s) used, how many reviewers assessed each study and whether they worked independently, and if applicable, details of automation tools used in the process. | 9 |
| Effect measures | 12 | Specify for each outcome the effect measure(s) (e.g. risk ratio, mean difference) used in the synthesis or presentation of results. | Not applicable. Not formal systematic review. |
| Synthesis methods | 13a | Describe the processes used to decide which studies were eligible for each synthesis (e.g. tabulating the study intervention characteristics and comparing against the planned groups for each synthesis (item #5)). | 9 |
| | 13b | Describe any methods required to prepare the data for presentation or synthesis, such as handling of missing summary statistics, or data conversions. | 9 |
| | 13c | Describe any methods used to tabulate or visually display results of individual studies and syntheses. | 9 |
| | 13d | Describe any methods used to synthesize results and provide a rationale for the choice(s). If meta-analysis was performed, describe the | 9 |

For peer review only

# PRISMA 2020 Checklist

| Section and Topic | Item # | Checklist item | Location where item is reported |
|---|---|---|---|
| | | model(s), method(s) to identify the presence and extent of statistical heterogeneity, and software package(s) used. | |
| | 13e | Describe any methods used to explore possible causes of heterogeneity among study results (e.g. subgroup analysis, meta-regression). | Not applicable. Not formal systematic review. |
| | 13f | Describe any sensitivity analyses conducted to assess robustness of the synthesized results. | Not applicable. Not formal systematic review. |
| Reporting bias assessment | 14 | Describe any methods used to assess risk of bias due to missing results in a synthesis (arising from reporting biases). | Not applicable. Not formal systematic review. |
| Certainty assessment | 15 | Describe any methods used to assess certainty (or confidence) in the body of evidence for an outcome. | Not applicable. Not formal systematic review. |
| **RESULTS** | | | |
| Study selection | 16a | Describe the results of the search and selection process, from the number of records identified in the search to the number of studies included in the review, ideally using a flow diagram. | 10 |
| | 16b | Cite studies that might appear to meet the inclusion criteria, but which were excluded, and explain why they were excluded. | Figure 1 |
| Study characteristics | 17 | Cite each included study and present its characteristics. | Figure 2 |
| Risk of bias in studies | 18 | Present assessments of risk of bias for each included study. | Not applicable. Not formal systematic review. |
| Results of individual studies | 19 | For all outcomes, present, for each study: (a) summary statistics for each group (where appropriate) and (b) an effect estimate and its precision (e.g. confidence/credible interval), ideally using structured tables or plots. | Table 1-3 |
| Results of syntheses | 20a | For each synthesis, briefly summarise the characteristics and risk of bias among contributing studies. | Not applicable. Not formal systematic review. |
| | 20b | Present results of all statistical syntheses conducted. If meta-analysis was done, present for each the summary estimate and its precision (e.g. confidence/credible interval) and measures of statistical heterogeneity. If comparing groups, describe the direction of the effect. | Not applicable. |

For peer review only

For peer review only - http://bmjopen.bmj.com/site/about/guidelines.xhtml

# PRISMA 2020 Checklist

| Section and Topic | Item # | Checklist item | Location where item is reported |
|---|---|---|---|
| | | | Not formal systematic review. |
| | 20c | Present results of all investigations of possible causes of heterogeneity among study results. | Not applicable. Not formal systematic review. |
| | 20d | Present results of all sensitivity analyses conducted to assess the robustness of the synthesized results. | Not applicable. Not formal systematic review. |
| Reporting biases | 21 | Present assessments of risk of bias due to missing results (arising from reporting biases) for each synthesis assessed. | Not applicable. Not formal systematic review. |
| Certainty of evidence | 22 | Present assessments of certainty (or confidence) in the body of evidence for each outcome assessed. | Not applicable. Not formal systematic review. |
| **DISCUSSION** | | | |
| Discussion | 23a | Provide a general interpretation of the results in the context of other evidence. | 14 |
| | 23b | Discuss any limitations of the evidence included in the review. | 17-18 |
| | 23c | Discuss any limitations of the review processes used. | 17-18 |
| | 23d | Discuss implications of the results for practice, policy, and future research. | 19-20 |
| **OTHER INFORMATION** | | | |
| Registration and protocol | 24a | Provide registration information for the review, including register name and registration number, or state that the review was not registered. | 9 |
| | 24b | Indicate where the review protocol can be accessed, or state that a protocol was not prepared. | 9 |
| | 24c | Describe and explain any amendments to information provided at registration or in the protocol. | 9 |
| Support | 25 | Describe sources of financial or non-financial support for the review, and the role of the funders or sponsors in the review. | 22 |
| Competing interests | 26 | Declare any competing interests of review authors. | 22 |
| Availability of data, code and other materials | 27 | Report which of the following are publicly available and where they can be found: template data collection forms; data extracted from included studies; data used for all analyses; analytic code; any other materials used in the review. | Not applicable. All data made available as |

For peer review only

# PRISMA 2020 Checklist

| Section and Topic | Item # | Checklist item | Location where item is reported |
|---|---|---|---|
| | | | supplementary information in this manuscript submission. |

*From:* Page MJ, McKenzie JE, Bossuyt PM, Boutron I, Hoffmann TC, Mulrow CD, et al. The PRISMA 2020 statement: an updated guideline for reporting systematic reviews. BMJ 2021;372:n71. doi: 10.1136/bmj.n71

For more information, visit: http://www.prisma-statement.org/

For peer review only
